# Effects of Antiplatelet Therapy After Stroke Caused by Intracerebral Hemorrhage: Extended Follow-up of the RESTART Randomized Clinical Trial

**DOI:** 10.1001/jamaneurol.2021.2956

**Published:** 2021-09-03

**Authors:** Rustam Al-Shahi Salman, Martin S. Dennis, Peter A. G. Sandercock, Cathie L. M. Sudlow, Joanna M. Wardlaw, William N. Whiteley, Gordon D. Murray, Jacqueline Stephen, Aryelly Rodriguez, Steff Lewis, David J. Werring, Phil M. White

**Affiliations:** 1 Centre for Clinical Brain Sciences, University of Edinburgh, Edinburgh, United Kingdom; 2 Usher Institute, University of Edinburgh, Edinburgh, United Kingdom; 3 Centre for Genomic and Experimental Medicine, University of Edinburgh, Edinburgh, United Kingdom; 4 UK Dementia Research Institute at the University of Edinburgh, University of Edinburgh, Edinburgh, United Kingdom; 5 Edinburgh Imaging, University of Edinburgh, Edinburgh, United Kingdom; 6 Stroke Research Group, Department of Brain Repair and Rehabilitation, University College London Queen Square Institute of Neurology, London, United Kingdom; 7 Department of Neuroradiology, Newcastle-upon-Tyne Hospitals National Health Service Trust, Newcastle-upon-Tyne, United Kingdom; 8 Institute of Neuroscience and Newcastle University Institute for Ageing, Newcastle University, Newcastle-upon-Tyne, United Kingdom

## Abstract

**Importance:**

The Restart or Stop Antithrombotics Randomized Trial (RESTART) found that antiplatelet therapy appeared to be safe up to 5 years after intracerebral hemorrhage (ICH) that had occurred during antithrombotic (antiplatelet or anticoagulant) therapy.

**Objectives:**

To monitor adherence, increase duration of follow-up, and improve precision of estimates of the effects of antiplatelet therapy on recurrent ICH and major vascular events.

**Design, Setting and Participants:**

From May 22, 2013, through May 31, 2018, this prospective, open, blinded end point, parallel-group randomized clinical trial studied 537 participants at 122 hospitals in the UK. Participants were individuals 18 years or older who had taken antithrombotic therapy for the prevention of occlusive vascular disease when they developed ICH, discontinued antithrombotic therapy, and survived for 24 hours. After initial follow-up ended on November 30, 2018, annual follow-up was extended until November 30, 2020, for a median of 3.0 years (interquartile range [IQR], 2.0-5.0 years) for the trial cohort.

**Interventions:**

Computerized randomization that incorporated minimization allocated participants (1:1) to start or avoid antiplatelet therapy.

**Main Outcomes and Measures:**

Participants were followed up for the primary outcome (recurrent symptomatic ICH) and secondary outcomes (all major vascular events) for up to 7 years. Data from all randomized participants were analyzed using Cox proportional hazards regression, adjusted for minimization covariates.

**Results:**

A total of 537 patients (median age, 76.0 years; IQR, 69.0-82.0 years; 360 [67.0%] male; median time after ICH onset, 76.0 days; IQR, 29.0-146.0 days) were randomly allocated to start (n = 268) or avoid (n = 269 [1 withdrew]) antiplatelet therapy. The primary outcome of recurrent ICH affected 22 of 268 participants (8.2%) allocated to antiplatelet therapy compared with 25 of 268 participants (9.3%) allocated to avoid antiplatelet therapy (adjusted hazard ratio, 0.87; 95% CI, 0.49-1.55; *P* = .64). A major vascular event affected 72 participants (26.8%) allocated to antiplatelet therapy compared with 87 participants (32.5%) allocated to avoid antiplatelet therapy (hazard ratio, 0.79; 95% CI, 0.58-1.08; *P* = .14).

**Conclusions and Relevance:**

Among patients with ICH who had previously taken antithrombotic therapy, this study found no statistically significant effect of antiplatelet therapy on recurrent ICH or all major vascular events. These findings provide physicians with some reassurance about the use of antiplatelet therapy after ICH if indicated for secondary prevention of major vascular events.

**Trial Registration:**

isrctn.org Identifier: ISRCTN71907627

## Key Points

**Question:**

What are the long-term effects of antiplatelet therapy after stroke caused by intracerebral hemorrhage in patients who were in the pilot phase of the Restart or Stop Antithrombotics Randomized Trial?

**Findings:**

In this randomized clinical trial of 537 participants with extended follow-up for a median total of 3.0 years (interquartile range, 2.0-5.0 years), the proportion of participants with intracerebral hemorrhage recurrence was 8.2% after allocation to start antiplatelet therapy and 9.3% without antiplatelet therapy, a nonsignificant difference. There were nonsignificantly fewer major vascular events among individuals in the group taking antiplatelet therapy vs the group avoiding antiplatelet therapy (26.8% vs 32.5%).

**Meaning:**

After intracerebral hemorrhage associated with antithrombotic drug use, antiplatelet therapy appears to be safe.

## Teaser

A follow-up of the Restart or Stop Antithrombotics Randomized Trial, this study further examines the long-term effects of antiplatelet therapy after stroke caused by intracerebral hemorrhage.

## Introduction

At least one-third of adults in high-income countries with stroke caused by spontaneous (nontraumatic) intracerebral hemorrhage (ICH) are already taking oral antithrombotic (antiplatelet or anticoagulant) drug therapy^1^ because of their comorbidities and other risk factors for vascular disease.^2^ Even after these risk factors are accounted for, survivors of spontaneous ICH are at higher risk of myocardial infarction and ischemic stroke than the general population,^3,4^ and their risk of all hemorrhagic or ischemic major vascular events is higher still at approximately 8% per year.^5,6,7^ Antiplatelet agents may benefit survivors of spontaneous ICH,^8,9^ but their effect on major vascular events is uncertain.^10^

A systematic review on June 11, 2021 (eTable 1 in Supplement 1), suggested that the Restart or Stop Antithrombotics Randomized Trial (RESTART) is the only published randomized clinical trial that compared the effects of starting vs avoiding antiplatelet therapy after ICH. RESTART included ICH survivors who had taken antithrombotic therapy to estimate the effects of starting vs avoiding antiplatelet therapy on recurrent symptomatic ICH and whether this risk might exceed any reduction of occlusive vascular events.^11,12,13^ After a median of 2 years of follow-up of 537 participants, RESTART’s main result excluded all but a very modest increase in the risk of recurrent ICH with antiplatelet therapy (adjusted hazard ratio [HR], 0.51; 95% CI, 0.25-1.03; *P* = .06).^14,15^ Therefore, the trial’s funder gave permission for the remainder of the funding award to be used to follow up all surviving participants willing to continue in the trial, which enabled us to extend follow-up for all surviving participants for up to 2 more years to improve the precision of estimates of effect during 3 years for most participants and provide information about effects up to 7 years for some participants.

## Methods

### Study Design

RESTART was an investigator-led, pragmatic, multicenter, prospective, open-label, blinded end point, parallel-group randomized clinical trial at 122 hospitals in the UK.^11,12,14,15^ The RESTART Steering Committee and the Academic and Clinical Central Office for Research and Development approved and published the trial protocol and the statistical analysis plan (Supplement 2).^11,12^ RESTART was approved by the Scotland A Research Ethics Committee. All data were deidentified before analysis. The study followed the Consolidated Standards of Reporting Trials (CONSORT) reporting guideline.

### Participants

We included adults (≥18 years of age) who had survived at least 24 hours after spontaneous ICH confirmed by brain imaging and were receiving antithrombotic therapy for the prevention of occlusive vascular disease at the onset of ICH, after which therapy was discontinued. Patients, or their nearest relative or representative if the patient did not have mental capacity, provided written informed consent in inpatient or outpatient hospital settings. Participants could be enrolled if they or their nearest relative, and their physician in secondary care, were uncertain about whether to start or avoid antiplatelet therapy and gave written informed consent.^12^

### Randomization and Blinding

Investigators supplied complete information about participants’ demographic characteristics, race/ethnicity (classified by investigators with participants to characterize the population), comorbidities, functional status, previous antithrombotic therapy, ICH, and their preferred antiplatelet therapy into a database via a secure web interface with in-built validation. A central computerized randomization system that incorporated a minimization algorithm randomly assigned participants (1:1) to start or avoid antiplatelet therapy. A detailed description of the minimization algorithm was published with the main results.^14^ The web interface displayed each participant’s allocation to starting or avoiding antiplatelet therapy; if the participant was allocated to start antiplatelet therapy, the system reminded investigators to prescribe the prespecified preferred antiplatelet therapy within 24 hours.

Treatment allocation was open to participants, practitioners caring for them in primary and secondary care, and local investigators. Staff conducting follow-up at the trial coordinating center were blinded to treatment allocation. Outcome event adjudicators were blinded to participant identity, treatment allocation, and drug use. When the main results were published,^14,15^ we mailed a summary of them written in plain English to surviving participants and their primary care practitioners and published them on the trial website in written and video format.^16^

### Procedures

Participants who had not already undergone magnetic resonance imaging (MRI) and who were able and willing to undergo brain MRI provided informed consent and underwent brain MRI according to the trial’s MRI protocol before randomization. After randomization, 1 of a panel of consultant neuroradiologists (P.M.W., David P. Minks, FRCR, Dipayan Mitra, MD, Priya Bhatnagar, FRCR, Johann C. du Plessis, FRCR, or Yogish Joshi, FRCR), blinded to treatment allocation, used the web-based Systematic Image Review System tool to review anonymized digital images of diagnostic brain computed tomography or MRI to confirm or refute eligibility and to support the adjudication of cerebral outcome events.

We restricted starting antiplatelet therapy to 1 or more of oral aspirin, dipyridamole, or clopidogrel, administered within 24 hours of randomization with doses determined at the discretion of the consultant responsible for the participant. The comparator was a policy of avoiding antiplatelet therapy. Participants could start or discontinue antithrombotic therapy if clinically indicated by events during follow-up. We measured adherence after randomization by recording antiplatelet therapy use before the first outcome event according to the preceding clinic or hospital discharge form or follow-up questionnaire. We collected information about blood pressure–lowering drugs and blood pressure control at discharge and during follow-up.

We followed up participants by sending a postal questionnaire to their primary care practitioners (who hold a comprehensive lifelong medical record for each patient registered with them), followed by a postal questionnaire to surviving participants (or caregivers) who had not withdrawn, to check survival, medication use, modified Rankin scale score, and the occurrence of outcomes. We sent questionnaires after randomization at 6 months or 1 year and then annually for up to 7 years. We interviewed participants who did not respond to the questionnaire by telephone.^17,18^ Extended follow-up was approved by the funder on February 25, 2019, by the research ethics committee on July 12, 2019, and by the sponsor on July 18, 2019, so that accrual of follow-up resumed on July 26, 2019, and ended on November 30, 2020.

We recorded serious adverse events (that were neither an outcome event nor an expected complication of stroke) via investigators if they occurred before hospital discharge or via primary care practitioners’ annual reports of hospital admissions. Investigators reported protocol deviations and violations to the trial coordinating center and the sponsor.

Monitoring included central statistical monitoring of trial conduct, data quality, and participant safety, supplemented by triggered onsite monitoring visits if required and detailed source data verification at the trial coordinating center. We conducted completeness, range, consistency, validation, and logic checks on all baseline and outcome data from the web-based case report forms.

### Outcomes

The primary outcome was fatal or nonfatal radiologically or pathologically proven recurrent symptomatic ICH assessed in all participants (except 1 participant who withdrew before the first follow-up). The secondary outcomes of major hemorrhagic events and major occlusive vascular events were prespecified.^11,12^ In the protocol, we had specified a composite secondary outcome of all major vascular events defined by the Antithrombotic Trialists’ Collaboration (nonfatal myocardial infarction, nonfatal stroke [ischemic, hemorrhagic, or uncertain cause], or death from a vascular cause).^8,12^ The process of outcome adjudication was described in the main report.^14,19,20^

### Statistical Analysis

We aimed to recruit 720 participants and continue their follow-up for at least 2 years to cover several combinations of published estimates of the primary outcome event rate in cohort studies (1.8%-7.4% per year)^21^ and an up to 4 times proportional increase in the absolute risk of the primary outcome with the use of antiplatelet therapy in observational studies.^22,23,24^ Throughout the recruitment period and during extended follow-up, unblinded trial statisticians supplied the independent data monitoring committee with analyses of the accumulating baseline and follow-up data at the same frequency and for the same purposes as described previously.^14^ Two statisticians (A.R. and S.L.) and the chief investigator (R.A.-S.S.) prepared an updated statistical analysis plan for the analysis of extended follow-up, which was approved by the RESTART Steering Committee before database lock. All group comparisons were made using the intention-to-treat populations. All group comparisons were made using the intention-to-treat populations.

We estimated survival in each treatment group using a Kaplan-Meier survival analysis of time to first occurrence of a primary or secondary outcome event during all available follow-up after randomization, censored at death unrelated to an outcome event or last available follow-up. We quantified completeness of follow-up as the proportion of participants with a complete follow-up questionnaire at each planned interval after randomization and as the proportion of the planned duration of follow-up that was observed.^25^ After assessing the proportional hazards assumption graphically and including a treatment by log(time) interaction, we compared survival by allocated treatment using the log-rank test. The primary method of analysis was to construct an unadjusted Cox proportional hazards regression model and a second model adjusted for all 5 covariates included in the minimization algorithm to calculate the HRs. We used the Mann-Whitney test to compare group summaries of modified Rankin scale scores by randomized group. We performed sensitivity analyses by adding symptomatic stroke of uncertain subtype or deaths of undetermined cause to the primary outcome.

We performed prespecified exploratory subgroup analyses of the primary outcome with statistical tests of interaction to estimate heterogeneity of treatment effect between the prespecified subgroups: the 5 covariates used by the minimization algorithm, antithrombotic therapy before ICH, and history of atrial fibrillation. The unblinded trial statistician (A.R.) performed statistical analyses with SAS statistical software, version 9.4 (SAS Institute Inc). A 2-sided *P* < .05 was considered to be statistically significant.

## Results

**Figure 1.**
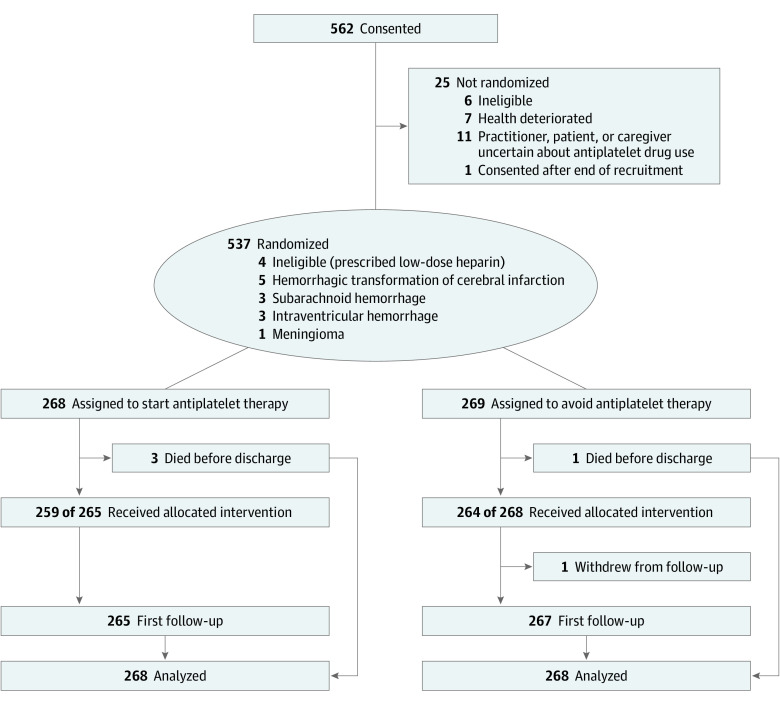
Flow Diagram Reprinted with permission from RESTART Collaboration.^14^

A total of 537 patients (median age, 76.0 years; IQR, 69.0-82.0 years; 360 [67.0%] male; median time after ICH onset, 76.0 days; IQR, 29.0-146.0 days) were randomly allocated to start (n = 268) or avoid (n = 269 [1 withdrew]) antiplatelet therapy. Between May 22, 2013, and May 31, 2018, a total of 562 participants consented to take part in the study from 104 of 122 activated hospital sites. A total of 268 participants were randomly assigned to start antiplatelet therapy and 269 to avoid antiplatelet therapy, of whom all but 1 participant was included in the outcome analyses. The flow diagram (Figure 1) and baseline characteristics (eTables 2 and 3 in Supplement 1) were unchanged from the main trial results. Extended follow-up ended on November 30, 2020. We obtained 1805 of a potential 1844 person-years of follow-up (overall completeness, 97.9%) for a median follow-up of 3.0 years (IQR, 2.0-5.0 years) per participant.

Immediate adherence to allocated treatment was good, with some decrease in adherence over time, but no clear overall change between the main results of the trial and when extended follow-up ended (eTable 4 in Supplement 1). Few participants used anticoagulant therapy during follow-up (eTable 5 in Supplement 1). Most participants took at least 1 blood pressure–lowering drug during follow-up and achieved a median systolic blood pressure of approximately 130 mm Hg, with good balance by treatment allocation (eTable 6 in Supplement 1). The proportional hazards assumption was fulfilled for analyses of primary and secondary outcomes during follow-up, apart from the composite outcome of any major hemorrhagic or occlusive event.

**Figure 2.**
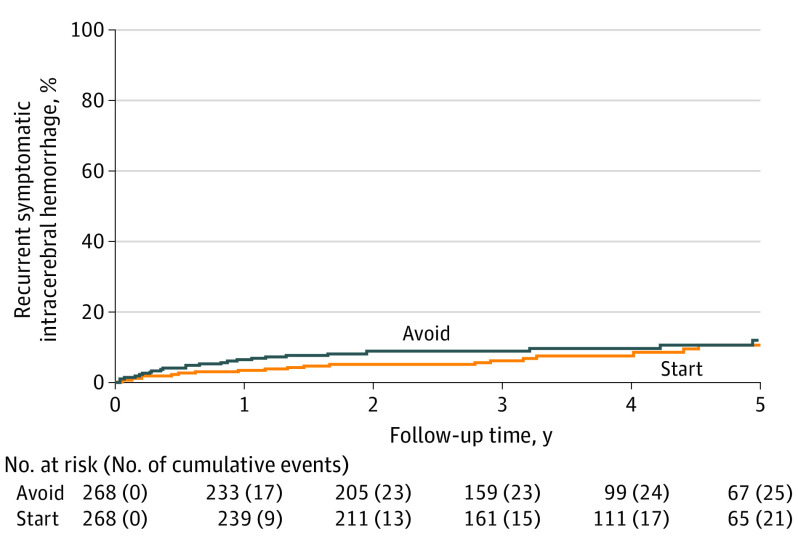
Risk of the First Occurrence of Recurrent Symptomatic Intracerebral Hemorrhage Numbers at risk refer to survivors undergoing follow-up at the start of each year according to treatment allocation. Plot was censored at 5 years. Cumulative events indicate the participants in follow-up with a first event.

For the primary outcome, 22 of 268 participants (8.2%) allocated to start antiplatelet therapy had recurrent ICH compared with 25 of 268 participants (9.3%) allocated to avoid antiplatelet therapy (adjusted HR, 0.87; 95% CI, 0.49-1.55; *P* = .64) (Table 1, Table 2, and Figure 2), without a significant risk difference in any individual year of follow-up (eTable 7 in Supplement 1). This finding was similar in unadjusted and adjusted models and in 2 sensitivity analyses that involved the addition of symptomatic stroke of uncertain subtype or death of undetermined cause (Table 2). At the time of their recurrent ICH, 3 of the 22 participants (13.6%) in the start antiplatelet therapy group were not taking an antiplatelet agent, and 6 of the 25 participants (24.0%) assigned to avoid antiplatelet therapy were taking an antiplatelet agent. There was no evidence of heterogeneity of the effects of antiplatelet therapy on the primary outcome in prespecified exploratory subgroup analyses (eFigure 1 in Supplement 1).

**Table 1.** Frequencies of the First Occurrence of Primary and Secondary Outcome Events During Follow-up

Outcome	No. (%) of participants	
	Start antiplatelet therapy (n = 268)	Avoid antiplatelet therapy (n = 268)
Recurrent symptomatic spontaneous intracerebral hemorrhage	22 (8.2)	25 (9.3)
Arterial events		
Major hemorrhagic events		
Spontaneous or traumatic intracranial extracerebral hemorrhage	6 (2.2)	4 (1.5)
Major extracranial hemorrhage	6 (2.2)	2 (0.7)
Major occlusive vascular events		
Ischemic stroke	28 (10.4)	39 (14.6)
Myocardial infarction	11 (4.1)	10 (3.7)
Peripheral arterial occlusion	8 (3.0)	3 (1.1)
Transient ischemic attack	13 (4.9)	26 (9.7)
Retinal arterial occlusion	0	0
Mesenteric ischemia	0	0
Stroke of uncertain subtype	1 (0.4)	5 (1.9)
Carotid, coronary, or peripheral arterial revascularization procedures	13 (4.9)	6 (2.2)
Venous events		
Deep vein thrombosis	8 (3.0)	2 (0.7)
Pulmonary embolism	5 (1.9)	1 (0.4)
Deaths		
Fatal outcome event	22 (8.2)	24 (9.0)
Other cardiovascular death	8 (3.0)	13 (4.9)
Sudden cardiac death	3 (1.1)	0
Noncardiovascular deaths	52 (19.4)	53 (19.8)
Deaths of undetermined cause	2 (0.7)	5 (1.9)

**Table 2.** Risks of First Occurrence of Primary and Secondary Outcome Events During Extended Follow-up

Outcome	No. (%) of participants		Unadjusted hazard ratio		Adjusted hazard ratio	
	Start antiplatelet therapy (n = 268)	Avoid antiplatelet therapy (n = 268)	Estimate (95% CI)	*P* value	Estimate (95% CI)	*P* value
Recurrent symptomatic spontaneous intracerebral hemorrhage	22 (8.2)	25 (9.3)	0.87 (0.49-1.54)	.63	0.87 (0.49-1.55)	.64
Recurrent symptomatic spontaneous intracerebral hemorrhage or symptomatic stroke of uncertain subtype	23 (8.6)	28 (10.4)	0.81 (0.47-1.41)	.46	0.81 (0.47-1.41)	.46
Recurrent symptomatic spontaneous intracerebral hemorrhage or death of undetermined cause	25 (9.3)	33 (12.3)	0.76 (0.45-1.27)	.29	0.75 (0.45-1.27)	.29
All major hemorrhagic events (all types of symptomatic spontaneous or traumatic intracranial hemorrhage or symptomatic major extracranial hemorrhage)	32 (11.9)	30 (11.2)	1.07 (0.65-1.75)	.80	1.07 (0.65-1.76)	.79
All major occlusive vascular events (ischemic stroke, myocardial infarction, mesenteric ischemia, peripheral arterial occlusion, deep vein thrombosis, pulmonary embolism, or carotid/coronary/peripheral arterial revascularization procedures)	57 (21.3)	53 (19.8)	1.08 (0.74-1.56)	.70	1.09 (0.75-1.59)	.64
Major occlusive vascular events (as proposed in the trial protocol)	65 (24.3)	71 (26.5)	0.90 (0.64-1.26)	.53	0.90 (0.64-1.26)	.55
Major vascular events (as proposed in the trial protocol: nonfatal myocardial infarction, nonfatal stroke, or death from a vascular cause [including sudden death, pulmonary embolism, hemorrhage, and death from an unknown cause])	72 (26.8)	87 (32.5)	0.79 (0.58-1.08)	.16	0.79 (0.58-1.08)	.14

**Figure 3.**
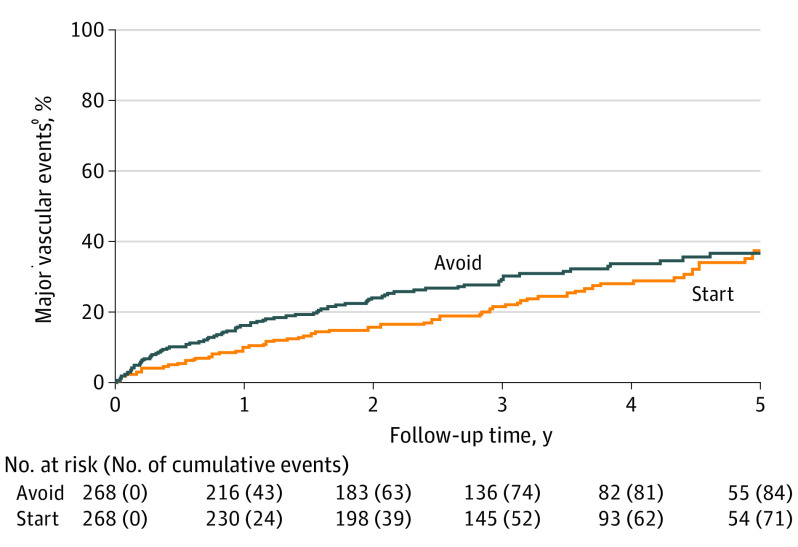
Risk of the First Occurrence of a Major Vascular Event Numbers at risk refer to survivors under follow-up at the start of each year according to treatment allocation. Plot was censored at 5 years. Cumulative events indicate the participants in follow-up with a first event.

For the composite secondary outcomes, no significant differences were found between participants assigned to start vs avoid antiplatelet therapy during the entire period of follow-up (Table 2). For the composite secondary outcome of all major vascular events (ie, nonfatal myocardial infarction, nonfatal stroke [ischemic, hemorrhagic, or uncertain cause], or death from a vascular cause) as used by the Antithrombotic Trialists Collaboration,^8,12^ which seemed to be reduced by starting antiplatelet therapy in the main results of the trial (adjusted HR, 0.65; 95% CI, 0.44-0.95; *P* = .03),^14^ no statistically significant reduction was found for the entire period of extended follow-up (Table 2). However, during the first 3 years of follow-up when almost all trial participants were followed up until they were censored or an outcome occurred, significant differences were found in the cumulative risk of all major vascular events between participants assigned to start vs avoid antiplatelet therapy after 1 year (cumulative risk difference, −7.0%; 95% CI, −12.6 to −1.4), 2 years (cumulative risk difference, −8.9%; 95% CI, −15.7 to −2.1), and 3 years (cumulative risk difference, −7.8%; 95% CI, −15.5 to −0.1) (Figure 3; eTable 7 in Supplement 1).

No differences were found in the distribution of the modified Rankin scale score during extended follow-up (eTable 8 in Supplement 1). Few serious adverse events occurred, which were neither outcomes nor expected complications of stroke (eTable 9 in Supplement 1).

## Discussion

During extended follow-up of participants in RESTART for a median of 3.0 years, survivors of an ICH that occurred while they were receiving antithrombotic therapy who were assigned to start antiplatelet therapy experienced similar numbers of ICH recurrences compared with participants assigned to avoid antiplatelet therapy. Effects on various secondary outcomes were not significant, but significantly fewer major vascular events occurred after allocation to start antiplatelet therapy after 1, 2, and 3 years.

Our findings are consistent with the expected effects of antiplatelet therapy.^8^ However, extended follow-up in RESTART still lacked the power to detect these effects with precision, despite a 70% increase in the total person-years of follow-up from 1064 to 1805, a 34% increase in primary outcomes from 35 to 47, and a 45% increase in major vascular events from 110 to 159.^14^ The change in the nonsignificant magnitude and precision of the effect of antiplatelet therapy on recurrent spontaneous ICH that we have observed after extended follow-up may reflect the small numbers of these outcomes in the main results^14^; the 95% CI of the estimate of the effect of aspirin on hemorrhagic stroke when used for secondary prevention of occlusive vascular disease (rate ratio, 1.67; 95% CI, 0.81–3.44) ^8^ includes the effect of antiplatelet therapy on recurrent spontaneous ICH in the extended follow-up of RESTART (adjusted HR, 0.87; 95% CI, 0.49-1.55). Furthermore, more than half of the reduction in major vascular events observed after starting vs avoiding antiplatelet therapy in the main results of RESTART (45 vs 65; adjusted HR, 0.65; 0.44-0.95) was attributable to the difference in recurrent spontaneous ICH (12 vs 23); the numbers of participants with recurrent spontaneous ICH after extended follow-up is similar (22 vs 25), making the observed differences in major vascular events in the first 3 years (eTable 8 in Supplement 1) less driven by differences in numbers with recurrent ICH.^14^

To our knowledge, RESTART remains the only completed randomized clinical trial that compares starting vs avoiding antiplatelet therapy after ICH.^10^ We minimized selection bias by using central, computerized random sequence generation and concealing allocation on the web application until all baseline data were entered. Event rates in RESTART were comparable to community-based studies in the UK.^5^ Blood pressure was controlled comparably between groups throughout follow-up. We minimized attrition bias by achieving greater than 97% completeness of extended follow-up. We continued to blind outcome assessors and used objective definitions of major outcomes and independent verification to reduce misclassification of hemorrhagic and occlusive vascular events and to reduce the bias that can arise in outcome assessment when treatment allocation is open.^26^

### Limitations

The sample size and number of outcomes in RESTART were too small to be definitive. Most participants were male.^27^ Although we did not blind the assigned treatment to participants and physicians, the outcomes were objective and adjudicated blinded to treatment allocation, which minimizes bias.^28^ Adherence to the allocated treatment decreased over time but was greater than 80% even after 5 years of follow-up and did not seem to be affected by the publication of the main results. Some of the secondary composite outcomes included venous thromboembolism, which may not be affected by antiplatelet therapy that principally reduces the risk of occlusive arterial events by reducing platelet activation and aggregation^29^; this effect may explain why an early effect of antiplatelet therapy was detected on major vascular events^8^ but not on other composite outcomes (Table 2). Participants were recruited from similar state-funded health care services in 4 countries of the UK, but the generalizability of our findings to other populations is unknown.

These findings alongside published observational studies^3,22,23,24,30,31,32,33,34,35^ provide reassurance about the use of long-term antiplatelet therapy after ICH associated with antithrombotic therapy. Since RESTART’s main results were published, 1 stroke guideline has recommended, “In patients with an indication for continued antiplatelet treatment, resuming antiplatelet therapy is reasonable (Evidence Level B).”^36^ However, we did not observe a change in participants’ adherence during extended follow-up after the main results were published, so patients and guidelines will require greater strength and certainty of evidence to change clinical practice.

A much larger randomized clinical trial could investigate several remaining uncertainties about antiplatelet therapy after ICH,^37,38,39,40^ including whether there is an overall reduction in major vascular events after ICH of similar magnitude to that seen in patients without ICH^8^; whether time since ICH and duration of antiplatelet therapy modify these effects^41^; whether effects vary in other subgroups (such as lobar ICH location, which is a risk factor for ICH recurrence^5,21^ [eFigures 1 and 2 in Supplement 1], or other imaging features that may modify risks of recurrent ICH or ischemic stroke^15,42^); and whether antiplatelet therapy is beneficial for ICH survivors without prior antithrombotic drug use or major vascular events.^3,5^

## Conclusions

After final, extended follow-up of the only completed randomized clinical trial of antiplatelet therapy after ICH, no statistically significant increase in the risk of recurrent ICH after restarting antiplatelet therapy was found. This finding provides physicians with some reassurance about the use of antiplatelet therapy after ICH if indicated for secondary prevention of major vascular events.

## Notes

Supplement 1. **eTable 1.** Appendix of Collaborators on Delegation Logs at Hospital Sites That Recruited at Least One Participant to RESTART **eTable 1.** Literature Search Strategies **eTable 2.** Participant Characteristics at Randomization by Treatment Allocation (as Published^1^) **eTable 3.** Antithrombotic Therapy Before Randomization (as Published^1^) **eTable 4.** Adherence to Allocated Treatment Strategy Before the First Outcome Event After Randomization **eTable 5.** Anticoagulant Therapy at Discharge and During Follow-up **eTable 6.** Blood Pressure-Lowering Drug Use at Discharge and During Follow-up, With Average Achieved Blood Pressures During Follow-up, by Treatment Allocation **eTable 7.** Cumulative Absolute Risks, and Risk Differences, of the Primary Outcome and Key Secondary Outcomes, by Treatment Allocation and by Year **eFigure 1.** Pre-specified Exploratory Sub-group Analyses of the Risk of First Recurrent Symptomatic Intracerebral Hemorrhage **eFigure 2.** Kaplan-Meier Plot of the Risk of the First Occurrence of Recurrent Symptomatic Intracerebral Hemorrhage, Stratified by Qualifying Intracerebral Hemorrhage Location **eTable 9.** Modified Rankin Scale (mRS) Scores Rated at Randomization by Collaborators and During Follow-up by Participants/Carers,^3,4^ by Treatment Allocation **eTable 10.** Number of Serious Adverse Events, Classified by MedDRA Preferred Term
eReferences

Supplement 2.
Trial Protocol and Statistical Analysis Plan

Supplement 3. **Nonauthor Collaborators.** RESTART Collaboration

Supplement 4.
Data Sharing Statement
