# Supplementary material for: Effects of Antiplatelet Therapy After Stroke Caused by Intracerebral Hemorrhage: Extended Follow-up of the RESTART Randomized Clinical Trial
Source: JAMA Neurol. 2021 Sep 3;78(10):1–8. doi: 10.1001/jamaneurol.2021.2956 (PMC8417806; doi:10.1001/jamaneurol.2021.2956)
Supplement: Supplement 1. — eTable 1. Appendix of Collaborators on Delegation Logs at Hospital Sites That Recruited at Least One Participant to RESTART eTable 1. Literature Search Strategies eTable 2. Participant Characteristics at Randomization by Treatment Allocation (as Published1) eTable 3. Antithrombotic Therapy Before Randomization (as Published1) eTable 4. Adherence to Allocated Treatment Strategy Before the First Outcome Event After Randomization eTable 5. Anticoagulant Therapy at Discharge and During Follow-up eTable 6. Blood Pressure-Lowering Drug Use at Discharge and During Follow-up, With Average Achieved Blood Pressures During Follow-up, by Treatment Allocation eTable 7. Cumulative Absolute Risks, and Risk Differences, of the Primary Outcome and Key Secondary Outcomes, by Treatment Allocation and by Year eFigure 1. Pre-specified Exploratory Sub-group Analyses of the Risk of First Recurrent Symptomatic Intracerebral Hemorrhage eFigure 2. Kaplan-Meier Plot of the Risk of the First Occurrence of Recurrent Symptomatic Intracerebral Hemorrhage, Stratified by Qualifying Intracerebral Hemorrhage Location eTable 9. Modified Rankin Scale (mRS) Scores Rated at Randomization by Collaborators and During Follow-up by Participants/Carers,3,4 by Treatment Allocation eTable 10. Number of Serious Adverse Events, Classified by MedDRA Preferred Term eReferences [file jamaneurol-e212956-s001.pdf]

Supplementary Online Content

Al-Shahi Salman R, Dennis MS, Sandercock PAG, et al; RESTART Collaboration. Effects of antiplatelet therapy after stroke caused by intracerebral hemorrhage: extended follow-up of the RESTART randomized clinical trial. *JAMA Neurol.* Published online September 3, 2021. doi:10.1001/jamaneurol.2021.2956

eTable 1. Literature Search Strategies

eTable 2. Participant Characteristics at Randomization by Treatment Allocation (as Published[1])

eTable 3. Antithrombotic Therapy Before Randomization (as Published[1])

eTable 4. Adherence to Allocated Treatment Strategy Before the First Outcome Event After Randomization

eTable 5. Anticoagulant Therapy at Discharge and During Follow-up

eTable 6. Blood Pressure-Lowering Drug Use at Discharge and During Follow-up, With Average Achieved Blood Pressures During Follow-up, by Treatment Allocation

eTable 7. Cumulative Absolute Risks, and Risk Differences, of the Primary Outcome and Key Secondary Outcomes, by Treatment Allocation and by Year

eFigure 1. Pre-specified Exploratory Sub-group Analyses of the Risk of First Recurrent Symptomatic Intracerebral Hemorrhage

eFigure 2. Kaplan-Meier Plot of the Risk of the First Occurrence of Recurrent Symptomatic Intracerebral Hemorrhage, Stratified by Qualifying Intracerebral Hemorrhage Location

eTable 8. Modified Rankin Scale (mRS) Scores Rated at Randomization by Collaborators and During Follow-up by Participants/Carers,[3,4] by Treatment Allocation

eTable 9. Number of Serious Adverse Events, Classified by MedDRA Preferred Term

References

This supplementary material has been provided by the authors to give readers additional information about their work.

© 2021 Al-Shahi Salman R et al. *JAMA Neurology*.

eTable 1. Literature search strategies

CENTRAL search strategy
#1 MESH DESCRIPTOR Basal Ganglia Hemorrhage EXPLODE ALL TREES
#2 MESH DESCRIPTOR Intracranial Hemorrhages
#3 MESH DESCRIPTOR Intracranial Hemorrhage, Hypertensive
#4 MESH DESCRIPTOR Cerebral Hemorrhage
#5 (brain* or cerebr* or cerebell* or intracerebral or intracran* or parenchymal or intraparenchymal or intraventricular or infratentorial or supratentorial or putaminal or putamen or hemispher* or stroke or apoplex*):TI
#6 (basal and gangli*):TI
#7 (posterior and fossa):TI,AB,KY
#8 (haemorrhag* or hemorrhag* or haematoma* or hematoma* or bleed*):TI
#9 #5 or #6 or #7 and #8
#10 (ICH or ICHs):TI
#11 #1 or #2 or #3 or #4 or #9 or #10
#12 MESH DESCRIPTOR Anticoagulants EXPLODE ALL TREES
#13 MESH DESCRIPTOR Pipecolic Acids EXPLODE ALL TREES WITH QUALIFIERS AE,TU
#14 MESH DESCRIPTOR Vitamin K EXPLODE ALL TREES
#15 MESH DESCRIPTOR Thrombin EXPLODE ALL TREES WITH QUALIFIERS AI
#16 MESH DESCRIPTOR Factor Xa
#17 MESH DESCRIPTOR Blood Coagulation Factors EXPLODE ALL TREES WITH QUALIFIERS AI
#18 MESH DESCRIPTOR Blood Coagulation EXPLODE ALL TREES WITH QUALIFIERS DE
#19 MESH DESCRIPTOR Antithrombins EXPLODE ALL TREES
#20 MESH DESCRIPTOR Hirudin Therapy EXPLODE ALL TREES
#21 (anticoagul* or antithromb*):TI,AB,KY
#22 (Vitamin next K next antagonist*):TI,AB,KY
#23 (VKA or VKAs):TI,AB,KY
#24 #22 or #23
#25 (direct* NEAR5 thrombin):TI,AB,KY
#26 "DTI":TI,AB,KY
#27 (factor next Xa NEAR5 inhib*):TI,AB,KY
#28 (factor next 10a NEAR5 inhib*):TI,AB,KY
#29 (fXa NEAR5 inhib*):TI,AB,KY
#30 (autoprothrombin NEAR5 inhib*):TI,AB,KY
#31 (thrombokinase NEAR5 inhib*):TI,AB,KY
#32 (acenocoumarol* or dicoumarol* or ethyl next biscoumacetate* or phenprocoumon* or warfarin* or ancrod* or citric next acid* or coumarin* or chromonar* or coumestro* or esculi* or ochratoxin* or umbelliferone* or dermatan next sulfate* or dextran* or edetic next acid* or enoxaparin* or gabexate* or heparin* or lmwh* or nadroparin* or pentosan next sulfuric next polyester* or phenindione* or protein next c or protein next s or tedelparin*):TI,AB,KY
#33 (tinzaparin or parnaparin or dalteparin or reviparin or danaparoid or lomoparan or org next 10172 or mesoglycan or polysaccharide next sulphate* or sp54 or sp-54 or md805 or md-805 or cy222 or cy-222 or cy216 or cy-216):TI,AB,KY
#34 (Marevan or Fragmin* or Fraxiparin* or Klexane):TI,AB,KY
#35 (argatroban or MD805 or MD-805 or dabigatran or ximelagatran or melagatran or efegatran or flovagatran or inogatran or napsagatran or bivalirudin or lepirudin or hirudin* or desirudin or desulfatohirudin or hirugen or hirulog or AZD0837 or bothrojaracin or odiparcil):TI,AB,KY
#36 (xabans or antistasin or apixaban or betrixaban or du next 176b or eribaxaban or fondaparinux or idraparinux or otamixaban or razaxaban or rivaroxaban or yagin or ym next 150 or ym150 or LY517717):TI,AB,KY
#37 #25 or #26 or #27 or #28 or #29 or #30 or #31 or #32 or #33 or #34 or #35 or #36
#38 MESH DESCRIPTOR Platelet Glycoprotein GPIIb-IIIa Complex EXPLODE ALL TREES WITH QUALIFIERS AI,DE
#39 MESH DESCRIPTOR Platelet Activation EXPLODE ALL TREES WITH QUALIFIERS DE
#40 MESH DESCRIPTOR Blood Platelets EXPLODE ALL TREES WITH QUALIFIERS DE
#41 (antiplatelet* or anti-platelet* or antiaggreg* or anti-aggreg* or (platelet* NEAR5 inhibit*) or (thrombocyt* NEAR5 inhibit*)):TI,AB,KY #42 (alprostadil* or aspirin* or acetylsalicylic next acid or (acetyl ADJ salicylic and acid*) or (acetyl-salicylic and acid or epoprostenol* or ketanserin* or ketorolac next tromethamine* or milrinone* or mopidamol* or procainamide* or thiophen* or trapidil* or picotamide* or ligustrazine* or levamisol* or suloctidil* or ozagrel* or oky046 or oky-046 or defibrotide* or cilostazol or satigrel or sarpolgrelate or kbt3022 or kbt-3022 or isbogrel or cv4151 or cv-4151)):TI,AB,KY
#43 ((glycoprotein next iib* near/5 inhib*) or (glycoprotein next iib* near/5 antag*) or (gp next iib* near/5 inhib*) or (gp next iib* near/5 antag*) or GR144053 or GR-144053 or triflusal):TI,AB,KY
#44 (Argatroban or Beraprost or Cicaprost or Cilostazol or Clopidogrel or Dipyridamole or Iloprost or Indobufen or Lepirudin or Pentosan next Polysulfate or Pentoxifylline or Piracetam or Prostacyclin or Sulfinpyrazone or Sulphinpyrazone or Ticlopidine or Triflusal or Abciximab or Disintegrin or Echistatin or Eptifibatide or Lamifiban or Orbofiban or Roxifiban or Sibrafiban or Tirofiban or Xemilofiban or terutroban or picotamide or prasugrel):TI,AB,KY
#45 (Dispril or Albyl* or Ticlid* or Persantin* or Plavix or ReoPro or Integrilin* or Aggrastat):TI,AB,KY
#46 MESH DESCRIPTOR Platelet Aggregation Inhibitors EXPLODE ALL TREES
#47 #38 or #39 or #40 or #41 or #42 or #43 or #44 or #45 or #46
#48 #37 or #47
#49 #48 and #11

© 2021 Al-Shahi Salman R et al. *JAMA Neurology*.

MEDLINE search strategy
1. exp basal ganglia haemorrhage/ or intracranial hemorrhages/ or cerebral haemorrhage/ or intracranial haemorrhage, hypertensive/
2. ((brain$ or cerebr$ or cerebell$ or intracerebral or intracran$ or parenchymal or intraparenchymal or intraventricular or infratentorial or supratentorial or basal gangli$ or putaminal or putamen or posterior fossa or hemispher$) adj5 (h?emorrhag$ or h?ematoma$ or bleed$)).tw.
3. ((h?emorrhag$ or bleed$) adj5 (stroke or apoplex$)).tw.
4. (ICH or ICHs).tw.
5. 1 or 2 or 3 or 4
6. exp anticoagulants/
7. exp Vitamin K/ai or thrombin/ai or factor Xa/ai or exp Blood coagulation factors/ai
8. exp antithrombins/ or hirudin therapy/
9. (anticoagul$ or antithromb$).tw.
10. (Vitamin K antagonist$ or VKA or VKAs).tw.
11. (direct$ adj3 thrombin adj3 inhib$).tw.
12. DTI$1.tw.
13. ((factor Xa or factor 10a or fXa or autoprothrombin c or thrombokinase) adj3 inhib$).tw.
14. (activated adj3 (factor X or factor 10) adj3 inhib$).tw.
15. (acenocoumarol$ or dicoumarol$ or ethyl biscoumacetate$ or phenprocoumon$ or warfarin$ or ancrod$ or citric acid$ or coumarin$ or chromonar$ or coumestro$ or esculi$ or ochratoxin$ or umbelliferone$ or dermatan sulfate$ or dextran$ or edetic acid$ or enoxaparin$ or gabexate$ or heparin$ or lmwh$ or nadroparin$ or pentosan sulfuric polyester$ or phenindione$ or protein c or protein s or tedelparin$).tw,nm.
16. (tinzaparin or parnaparin or dalteparin or reviparin or danaparoid or lomoparan or org 10172 or mesoglycan or polysaccharide sulphate$ or sp54 or sp-54 or md805 or md-805 or cy222 or cy-222 or cy216 or cy-216).tw,nm.
17. (Marevan or Fragmin$ or Fraxiparin$ or Klexane).tw,nm.
18. (argatroban or MD805 or MD-805 or dabigatran or ximelagatran or melagatran or efegatran or flovagatran or inogatran or napsagatran or bivalirudin or lepirudin or hirudin$ or desirudin or desulfatohirudin or hirugen or hirulog or AZD0837 or bothrojaracin or odiparcil).tw,nm.
19. (xabans or antistasin or apixaban or betrixaban or du 176b or eribaxaban or fondaparinux or idraparinux or otamixaban or razaxaban or rivaroxaban or yagin or ym 150 or ym150 or LY517717).tw,nm.
20. exp platelet aggregation inhibitors/ or exp platelet glycoprotein gpiib-iiia complex/ai
21. (antiplatelet$ or anti-platelet$ or antiaggreg$ or anti-aggreg$ or (platelet$ adj3 inhibit$) or (thrombocyt$ adj3 inhibit$)).tw.
22. (alprostadil$ or aspirin$ or acetylsalicylic acid or acetyl salicylic acid$ or acetyl?salicylic acid or epoprostenol$ or ketanserin$ or ketorolac tromethamine$ or milrinone$ or mopidamol$ or procainamide$ or thiophen$ or trapidil$ or picotamide$ or ligustrazine$ or levamisol$ or suloctidil$ or ozagrel$ or oky046 or oky-046 or defibrotide$ or cilostazol or satigrel or sarpolgrelate or kbt3022 or kbt-3022 or isbogrel or cv4151 or cv-4151 or ((glycoprotein iib$ or gp iib$) adj5 (antagonist$ or inhibitor$)) or GR144053 or GR-144053 or triflusal).tw,nm.
23. (Beraprost or Cicaprost or Cilostazol or Clopidogrel or Dipyridamole or Iloprost or Indobufen or Lepirudin or Pentosan Polysulfate or Pentoxifylline or Piracetam or Prostacyclin or Sulfinpyrazone or Sulphinpyrazone or Ticlopidine or Triflusal or Abciximab or Disintegrin or Echistatin or Eptifibatide or Lamifiban or Orbofiban or Roxifiban or Sibrafiban or Tirofiban or Xemilofiban or terutroban or picotamide or prasugrel).tw,nm.
24. (Dispril or Albyl$ or Ticlid$ or Persantin$ or Plavix or ReoPro or Integrilin$ or Aggrastat).tw,nm.
25. or/6-24
26. Randomized Controlled Trials as Topic/
27. random allocation/
28. Controlled Clinical Trials as Topic/
29. control groups/
30. clinical trials as topic/ or clinical trials, phase i as topic/ or clinical trials, phase ii as topic/ or clinical trials, phase iii as topic/ or clinical trials, phase iv as topic/
31. double-blind method/
32. single-blind method/
33. Placebos/
34. placebo effect/
35. randomised controlled trial.pt.
36. controlled clinical trial.pt.
37. (clinical trial or clinical trial phase i or clinical trial phase ii or clinical trial phase iii or clinical trial phase iv).pt.
38. (random$ or RCT or RCTs).tw.
39. (controlled adj5 (trial$ or stud$)).tw.
40. (clinical$ adj5 trial$).tw.
41. ((control or treatment or experiment$ or intervention) adj5 (group$ or subject$ or patient$)).tw.
42. (quasi-random$ or quasi random$ or pseudo-random$ or pseudo random$).tw.
43. ((control or experiment$ or conservative) adj5 (treatment or therapy or procedure or manage$)).tw.
44. ((singl$ or doubl$ or tripl$ or trebl$) adj5 (blind$ or mask$)).tw.
45. (placebo$ or sham).tw.
46. trial.ti.
47. (assign$ or allocat$).tw.
48. or/26-47
49. 5 and 25 and 48
50. exp animals/ not humans/
51. 49 not 50

© 2021 Al-Shahi Salman R et al. *JAMA Neurology*.

EMBASE (Ovid) search strategy

1. anticoagulant agent/ or antivitamin k/ or exp blood clotting inhibitor/ or exp coumarin anticoagulant/ or defibrotide/ or dextran sulfate/ or fluindione/ or glycosaminoglycan polysulfate/ or exp heparin derivative/ or lupus anticoagulant/ or phenindione/
2. (anticoagul$ or antithromb$).tw.
3. (Vitamin K antagonist$ or VKA or VKAs).tw.
4. (direct$ adj5 thrombin adj5 inhib$).tw.
5. DTI$1.tw.
6. ((factor Xa or factor 10a or fXa or autoprothrombin c or thrombokinase) adj5 inhib$).tw.
7. (activated adj5 (factor X or factor 10) adj5 inhib$).tw.
8. (acenocoumarol$ or dicoumarol$ or ethyl biscoumacetate$ or phenprocoumon$ or warfarin$ or ancrod$ or citric acid$ or coumarin$ or chromonar$ or coumestro$ or esculi$ or ochratoxin$ or umbelliferone$ or dermatan sulfate$ or dextran$ or edetic acid$ or enoxaparin$ or gabexate$ or heparin$ or lmwh$ or nadroparin$ or pentosane sulfuric polyester$ or phenindione$ or protein c or protein s or tedelparin$).tw.
9. (tinzaparin or parnaparin or dalteparin or reviparin or danaparoid or lomoparan or org 10172 or mesoglycan or polysaccharide sulphate$ or sp54 or sp-54 or md805 or md-805 or cy222 or cy-222 or cy216 or cy-216).tw.
10. (Marevan or Fragmin$ or Fraxiparin$ or Klexane).tw.
11. (argatroban or MD805 or MD-805 or dabigatran or ximelagatran or melagatran or efegatran or flovagatran or inogatran or napsagatran or bivalirudin or lepirudin or hirudin$ or desirudin or desulfatohirudin or hirugen or hirulog or AZD0837 or bothrojaracin or odiparcil).tw.
12. (xabans or antistasin or apixaban or betrixaban or du 176b or eribaxaban or fondaparinux or idraparinux or otamixaban or razaxaban or rivaroxaban or yagin or ym 150 or ym150 or LY517717).tw.
13. or/1-12
14. exp antithrombocytic agent/
15. fibrinogen receptor/dt [Drug Therapy]
16. (antiplatelet$ or anti-platelet$ or antiaggreg$ or anti-aggreg$ or (platelet$ adj5 inhibit$) or (thrombocyt$ adj5 inhibit$)).tw.
17. (alprostadil$ or aspirin$ or acetylsalicylic acid or acetyl salicylic acid$ or acetyl?salicylic acid or epoprostenol$ or ketanserin$ or ketorolac tromethamine$ or milrinone$ or mopidamol$ or procainamide$ or thiophen$ or trapidil$ or picotamide$ or ligustrazine$ or levamisol$ or suloctidil$ or ozagrel$ or oky046 or oky-046 or defibrotide$ or cilostazol or satigrel or sarpolgrelate or kbt3022 or kbt-3022 or isbogrel or cv4151 or cv-4151 or ((glycoprotein iib$ or gp iib$) adj5 (antagonist$ or inhibitor$)) or GR144053 or GR-144053 or triflusal).tw.
18. (Argatroban or Beraprost or Cicaprost or Cilostazol or Clopidogrel or Dipyridamole or Iloprost or Indobufen or Lepirudin or Pentosan Polysulfate or Pentoxifylline or Piracetam or Prostacyclin or Sulfinpyrazone or Sulphinpyrazone or Ticlopidine or Triflusal or Abciximab or Disintegrin or Echistatin or Eptifibatide or Lamifiban or Orbofiban or Roxifiban or Sibrafiban or Tirofiban or Xemilofiban or terutroban or picotamide or prasugrel).tw.
19. (Dispril or Albyl$ or Ticlid$ or Persantin$ or Plavix or ReoPro or Integrilin$ or Aggrastat).tw.
20. or/14-19
21. 13 or 20
22. *basal ganglion hemorrhage/ or *brain hemorrhage/ or *brain ventricle hemorrhage/ or *cerebellum hemorrhage/
23. ((brain$ or cerebr$ or cerebell$ or intracerebral or intracran$ or parenchymal or intraparenchymal or intraventricular or infratentorial or supratentorial or basal gangli$ or putaminal or putamen or posterior fossa or hemispher$ or stroke or apoplex$) adj5 (h?emorrhag$ or h?ematoma$ or bleed$)).ti.
24. 22 or 23 or (ICH or ICHs).ti.
25. randomized controlled trial/ or "randomized controlled trial (topic)"/
26. Randomization/
27. Controlled Study/
28. control group/
29. clinical trial/ or phase 1 clinical trial/ or phase 2 clinical trial/ or phase 3 clinical trial/ or phase 4 clinical trial/ or controlled clinical trial/
30. Double Blind Procedure/
31. Single Blind Procedure/ or triple blind procedure/
32. placebo/
33. drug comparison/ or drug dose comparison/
34. "types of study"/
35. random$.tw.
36. (controlled adj5 (trial$ or stud$)).tw.
37. (clinical$ adj5 trial$).tw.
38. ((control or treatment or experiment$ or intervention or surgical) adj5 (group$ or subject$ or patient$)).tw.
39. (quasi-random$ or quasi random$ or pseudo-random$ or pseudo random$).tw.
40. ((singl$ or doubl$ or tripl$ or trebl$) adj5 (blind$ or mask$)).tw.
41. placebo$.tw.
42. controls.tw.
43. or/25-42
44. meta analysis/ or "meta analysis (topic)"/ or "systematic review"/ or "systematic review (topic)"/
45. meta analy$.tw.
46. metaanaly$.tw.
47. (systematic adj (review$1 or overview$1)).tw.
48. literature/

© 2021 Al-Shahi Salman R et al. *JAMA Neurology*.

49. (cochrane or embase or psychlit or psyclit or psychinfo or psycinfo or cinahl or cinhal or science citation index or bids or medline or pubmed).ab.
50. (reference list$ or bibliograph$ or hand-search$ or relevant journals or manual search).ab.
51. (selection criteria or data extraction).ab.
52. review.pt. or literature/ or review/
53. 51 and 52
54. 44 or 45 or 46 or 47 or 48 or 49 or 50 or 53
55. (letter or editorial).pt.
56. 54 not 55
57. 43 or 56
58. 21 and 24 and 57
59. limit 58 to human

Trials register search strategies

US National Institutes of Health Ongoing Trials Register ClinicalTrials.gov (www.clinicaltrials.gov)
AREA[StudyType] EXPAND[Term] COVER[FullMatch] "Interventional" AND AREA[ConditionSearch] intracerebral haemorrhage

World Health Organization (WHO) International Clinical Trials Registry Platform (www.who.int/ictrp/en/)
Basic search: intracerebral haemorrhage OR intracerebral hemorrhage OR ICH
Phases are: ALL

© 2021 Al-Shahi Salman R et al. *JAMA Neurology*.

eTable 2. Participant characteristics at randomization by treatment allocation (as published[1])

	**Start antiplatelet therapy (n=268)**		**Avoid antiplatelet therapy (n=269)**	
**Sex**				
Male	173	(65%)	187	(70%)
Female	95	(35%)	82	(30%)
**Age**[§]				
Median, years	77	(69–82)	76	(69–82)
<70 years	73	(27%)	73	(27%)
≥70 years	195	(73%)	196	(73%)
**Ethnicity**				
White	251	(94%)	242	(90%)
Asian	12	(4%)	18	(7%)
Black	4	(1%)	5	(2%)
Other	1	(<1%)	4	(1%)
**Indication for antithrombotic therapy before intracerebral hemorrhage ***				
At least one occlusive vascular disease				
With atrial fibrillation	42	(16%)	50	(19%)
Without atrial fibrillation	194	(72%)	189	(70%)
No occlusive vascular diseases				
With atrial fibrillation	19	(7%)	23	(9%)
Without atrial fibrillation	13	(5%)	7	(3%)
**Occlusive vascular diseases before intracerebral hemorrhage**				
Ischemic heart disease	133	(50%)	110	(41%)
Ischemic stroke	75	(28%)	88	(33%)
Transient ischemic attack	57	(21%)	76	(28%)
Atrial fibrillation/flutter	42	(16%)	50	(19%)
Peripheral arterial disease	22	(8%)	14	(5%)
Valvular heart disease	11	(4%)	18	(7%)
Symptomatic venous thromboembolism	9	(3%)	10	(4%)
Stroke of uncertain pathological type	2	(<1%)	3	(1%)
Retinal artery occlusion	3	(<1%)	5	(2%)
Mesenteric ischemia	1	(<1%)	1	(<1%)
**Past history of hemorrhage before intracerebral hemorrhage**				
Intracranial or extracranial hemorrhage	22	(8%)	25	(9%)
Intracerebral hemorrhage	8	(3%)	11	(4%)
Gastrointestinal hemorrhage	7	(3%)	6	(2%)
Other type of intracranial hemorrhage	5	(2%)	6	(2%)
Other type of extracranial hemorrhage	3	(1%)	2	(1%)
**Other relevant co-morbidities before intracerebral hemorrhage**				
High blood pressure	194	(72%)	207	(77%)
Diabetes mellitus	57	(21%)	70	(26%)
Congestive cardiac failure	12	(5%)	8	(3%)
Renal failure on dialysis	3	(1%)	3	(1%)
**Functional status**[2]				
Able to lift both arms off the bed	242	(90%)	244	(91%)

© 2021 Al-Shahi Salman R et al. *JAMA Neurology*.

	Start antiplatelet therapy (n=268)		Avoid antiplatelet therapy (n=269)	
Able to walk without the help of another person	199	(74%)	196	(73%)
Able to talk and not confused	234	(87%)	238	(89%)
**Location of intracerebral hemorrhage§**				
Lobar supratentorial	166	(62%)	166	(62%)
Non-lobar	102	(38%)	103	(38%)
**Time since intracerebral hemorrhage symptom onset §**				
Median, days	80	(30–149)	71	(29–144)
1–6 days	10	(4%)	11	(4%)
7–30 days	59	(22%)	59	(22%)
>30 days	199	(74%)	199	(74%)
**Probability of good six-month outcome § [2]**				
<0·15	48	(18%)	51	(19%)
≥0·15	220	(82%)	218	(81%)
**Context of enrolment**				
Hospital inpatient	87	(32%)	96	(36%)
Hospital outpatient	181	(68%)	173	(64%)
Participant consented	212	(79%)	213	(79%)
Proxy consented	56	(21%)	56	(21%)

Data are n (%) or median (inter-quartile range). * Some participants had more than one co-morbidity § Variables used in the minimization algorithm

© 2021 Al-Shahi Salman R et al. *JAMA Neurology*.

eTable 3. Antithrombotic therapy before randomization (as published[1])

	Start antiplatelet therapy (n=268)		Avoid antiplatelet therapy (n=269)	
**Antithrombotic therapy used before intracerebral hemorrhage**				
Antiplatelet therapy				
Aspirin monotherapy	132	(49%)	137	(51%)
Clopidogrel monotherapy	70	(26%)	63	(23%)
Aspirin and Clopidogrel	9	(3%)	5	(2%)
Aspirin and Dipyridamole	6	(2%)	5	(2%)
Other	4	(1%)	2	(1%)
Anticoagulant therapy				
Vitamin K antagonist	30	(11%)	41	(15%)
Non-vitamin K antagonist	8	(3%)	11	(4%)
Antiplatelet and anticoagulant therapy	9	(3%)	5	(2%)
**Preferred antiplatelet therapy that would be prescribed if allocated to start antiplatelet therapy ***				
Aspirin monotherapy	149	(56%)	150	(56%)
Clopidogrel monotherapy	117	(44%)	112	(42%)
Aspirin and Clopidogrel	1	(<1%)	5	(2%)
Dipyridamole monotherapy	1	(<1%)	1	(<1%)
Aspirin, Clopidogrel and Dipyridamole	0	(0%)	1	(<1%)

* Variable used in the minimization algorithm

© 2021 Al-Shahi Salman R et al. *JAMA Neurology*.

eTable 4. Adherence to allocated treatment strategy before the first outcome event after randomization

Follow-up time	Results of main follow-up		Results after extended follow-up	
	Start antiplatelet therapy (n=268)	Avoid antiplatelet therapy (n=269)	Start antiplatelet therapy (n=268)	Avoid antiplatelet therapy (n=269)
Hospital/clinic discharge	259/265 (98%)	266/266 (99%)	259/265 (98%)	264/266 (99%)
First year	193/218 (89%)	205/211 (97%)	194/217 (89%)	204/210 (97%)
Second year	104/122 (85%)	105/113 (93%)	158/182 (87%)	154/169 (91%)
Third year	59/71 (83%)	61/69 (88%)	108/129 (84%)	110/126 (87%)
Fourth year	21/26 (81%)	20/24 (83%)	64/81 (79%)	61/77 (79%)
Fifth year	2/2 (100%)	3/4 (75%)	33/40 (83%)	44/51 (86%)
Sixth year	-	-	7/12 (58%)	12/17 (71%)
Seventh year	-	-	1/2 (50%)	3/3 (100%)

Denominators indicate the number of participants surviving at each follow-up timepoint without a preceding outcome event

© 2021 Al-Shahi Salman R et al. *JAMA Neurology*.

eTable 5. Anticoagulant therapy at discharge and during follow-up

	Start antiplatelet therapy (n=268)		Avoid antiplatelet therapy (n=269)	
Hospital/clinic discharge	1/268	(<1%)	1/269	(<1%)
First annual follow-up	8/265	(3%)	6/267	(2%)
Second annual follow-up	9/240	(4%)	7/239	(3%)
Third annual follow-up	14/191	(7%)	6/191	(3%)
Fourth annual follow-up	6/126	(5%)	4/131	(3%)
Fifth annual follow-up	8/74	(11%)	8/81	(9%)
Sixth annual follow-up	3/24	(13%)	2/31	(6%)
Seventh annual follow-up	0/3	(0%)	0/6	(0%)

Denominators indicate the number of participants surviving at each follow-up timepoint

© 2021 Al-Shahi Salman R et al. *JAMA Neurology*.

eTable 6. Blood pressure-lowering drug use at discharge and during follow-up, with average achieved blood pressures during follow-up, by treatment allocation

	Start antiplatelet therapy		Avoid antiplatelet therapy	
**Hospital/clinic discharge**	**n=268**		**n=269**	
None	53	(20%)	41	(15%)
One	82	(31%)	92	(34%)
Two	92	(34%)	84	(31%)
Three	30	(11%)	36	(14%)
Four or more	11	(4%)	16	(6%)
**First annual follow-up**	**n=265**		**n=267**	
Median systolic blood pressure, mmHg	132	(120-140)	130	(121-140)
Median diastolic blood pressure, mmHg	74	(70-80)	74	(69-80)
None	46	(17%)	33	(12%)
One	51	(19%)	76	(29%)
Two	86	(33%)	77	(29%)
Three	55	(21%)	51	(19%)
Four or more	27	(10%)	30	(11%)
**Second annual follow-up**	**n=240**		**n=239**	
Median systolic blood pressure, mmHg	130	(120-139)	130	(120-139)
Median diastolic blood pressure, mmHg	72	(67-80)	72	(68-80)
None	48	(20%)	36	(15%)
One	53	(22%)	55	(23%)
Two	65	(27%)	81	(34%)
Three	45	(19%)	42	(18%)
Four or more	29	(12%)	25	(11%)
**Third annual follow-up**	**n=191**		**n=191**	
Median systolic blood pressure, mmHg	130	(120-140)	130	(120-140)
Median diastolic blood pressure, mmHg	75	(68-82)	70	(68-80)
None	43	(23%)	28	(15%)
One	51	(27%)	46	(24%)
Two	46	(24%)	60	(31%)
Three	36	(19%)	36	(19%)
Four or more	15	(8%)	21	(11%)
**Fourth annual follow-up**	**n=126**		**n=131**	
Median systolic blood pressure, mmHg	130	(120-140)	130	(121-140)
Median diastolic blood pressure, mmHg	77	(68-80)	74	(68-80)
None	25	(20%)	25	(19%)
One	35	(28%)	27	(21%)
Two	36	(29%)	34	(26%)
Three	23	(18%)	29	(22%)
Four or more	7	(5%)	16	(12%)
**Fifth annual follow-up**	**n=74**		**n=81**	
Median systolic blood pressure, mmHg	135	(123-140)	131	(125-136)
Median diastolic blood pressure, mmHg	74	(70-82)	70	(65-77)
None	12	(16%)	17	(21%)

© 2021 Al-Shahi Salman R et al. *JAMA Neurology*.

	Start antiplatelet therapy		Avoid antiplatelet therapy	
One	22	(30%)	15	(18%)
Two	19	(26%)	21	(26%)
Three	13	(17%)	16	(20%)
Four or more	8	(11%)	12	(15%)
**Sixth annual follow-up**	**n=24**		**n=31**	
Median systolic blood pressure, mmHg	136	(128-145)	130	(126-138)
Median diastolic blood pressure, mmHg	70	(69-82)	76	(65-81)
None	4	(17%)	5	(16%)
One	4	(17%)	9	(29%)
Two	11	(45%)	6	(19%)
Three	4	(17%)	7	(23%)
Four or more	1	(4%)	4	(13%)
**Seventh annual follow-up**	**n=3**		**n=6**	
Median systolic blood pressure, mmHg	140	(122-146)	142	(130-171)
Median diastolic blood pressure, mmHg	75	(64-82)	76	(62-82)
None	2	(67%)	1	(17%)
One	0	(0%)	2	(33%)
Two	1	(33%)	3	(50%)
Three	0	(0%)	0	(0%)
Four or more	0	(0%)	0	(0%)

Data are n (%) or median (inter-quartile range).

© 2021 Al-Shahi Salman R et al. *JAMA Neurology*.

eTable 7. Cumulative absolute risks, and risk differences, of the primary outcome and key secondary outcomes, by treatment allocation and by year

	Start antiplatelet therapy (n=268)	Avoid antiplatelet therapy (n=268)	Risk difference
**Recurrent symptomatic spontaneous intracerebral hemorrhage (primary outcome)**			
Year 1	3·5 (1·8 to 6·5)	6·5 (4·1 to 10·2)	-3·0 (-6·7 to 0·7)
Year 2	5·1 (3·0 to 8·6)	8·9 (6·0 to 13·2)	-3·9 (-8·3 to 0·6)
Year 3	6·2 (3·8 to 10·1)	8·9 (6·0 to 13·2)	-2·8 (-7·4 to 1·9)
Year 4	7·5 (4·7 to 12·0)	9·6 (6·5 to 14·0)	-2·1 (-7·2 to 3·0)
Year 5	12·0 (7·6 to 18·8)	10·6 (7·2 to 15·6)	1·4 (-5·5 to 8·3)
Year 6	13·4 (8·5 to 20·8)	10·6 (7·2 to 15·6)	2·8 (-4·6 to 10·1)
Year 7	13·4 (8·5 to 20·8)	10·6 (7·2 to 15·6)	2·8 (-4·6 to 10·1)
**All major hemorrhagic events (all types of symptomatic spontaneous or traumatic intracranial hemorrhage, or symptomatic major extracranial hemorrhage)**			
Year 1	5·4 (3·2 to 8·9)	7·2 (4·7 to 11·1)	-1·8 (-6·0 to 2·3)
Year 2	8·2 (5·4 to 12·3)	9·7 (6·6 to 14·0)	-1·4 (-6·4 to 3·5)
Year 3	9·3 (6·3 to 13·7)	10·7 (7·4 to 15·2)	-1·4 (-6·7 to 3·9)
Year 4	10·6 (7·2 to 15·4)	11·3 (7·9 to 16·1)	-0·7 (-6·4 to 5·0)
Year 5	17·4 (11·9 to 25·0)	13·6 (9·4 to 19·5)	3·8 (-4·4 to 12·0)
Year 6	18·7 (12·9 to 26·7)	13·6 (9·4 to 19·5)	5·1 (-3·4 to 13·6)
Year 7	18·7 (12·9 to 26·7)	13·6 (9·4 to 19·5)	5·1 (-3·4 to 13·6)
**All major occlusive vascular events (symptomatic ischemic stroke, myocardial infarction, mesenteric ischemia, peripheral arterial occlusion, deep vein thrombosis, pulmonary embolism, or carotid/coronary/peripheral arterial revascularization procedures)**			
Year 1	7·8 (5·1 to 11·9)	9·6 (6·6 to 13·9)	-1·8 (-6·7 to 3·1)
Year 2	13·2 (9·6 to 18·1)	13·8 (10·1 to 18·7)	-0·6 (-6·5 to 5·4)
Year 3	18·2 (13·8 to 23·8)	17·9 (13·6 to 23·5)	0·3 (-6·7 to 7·3)
Year 4	25·4 (19·8 to 32·3)	22·4 (17·2 to 28·9)	3·0 (-5·5 to 11·6)
Year 5	30·6 (23·7 to 39·0)	24·7 (18·9 to 31·9)	6·0 (-4·0 to 16·0)
Year 6	30·6 (23·7 to 39·0)	32·5 (22·2 to 45·9)	-1·8 (-15·9 to 12·3)
Year 7	30·6 (23·7 to 39·0)	32·5 (22·2 to 45·9)	-1·8 (-15·9 to 12·3)
**All major hemorrhagic or occlusive vascular events**			
Year 1	12·7 (9·2 to 17·4)	16·2 (12·3 to 21·3)	-3·5 (-9·5 to 2·5)
Year 2	20·4 (15·9 to 25·9)	22·3 (17·7 to 27·8)	-1·9 (-9·0 to 5·2)
Year 3	25·3 (20·2 to 31·3)	27·2 (22·1 to 33·3)	-1·9 (-9·8 to 5·9)
Year 4	32·7 (27·6 to 39·6)	30·6 (25·0 to 37·2)	2·0 (-6·9 to 10·9)
Year 5	43·8 (35·9 to 52·5)	33·8 (27·5 to 41·0)	10·0 (-0·8 to 20·8)
Year 6	45·0 (40·0 to 53·8)	41·0 (30·7 to 53·2)	4·0 (-10·2 to 18·2)
Year 7	45·0 (40·0 to 53·8)	41·0 (30·7 to 53·2)	4·0 (-10·2 to 18·2)
**All major occlusive vascular events (protocol-defined)**			
Year 1	10·1 (7·0 to 14·6)	14·3 (10·6 to 19·2)	-4·2 (-9·8 to 1·5)
Year 2	16·8 (12·7 to 22·1)	20·2 (15·7 to 25·7)	-3·3 (-10·1 to 3·5)
Year 3	21·8 (17·1 to 27·7)	24·7 (19·7 to 30·8)	-2·9 (-10·6 to 4·8)
Year 4	28·6 (22·7 to 35·6)	29·2 (23·4 to 35·9)	-0·6 (-9·6 to 8·4)
Year 5	33·9 (26·8 to 42·4)	33·8 (27·0 to 41·6)	0·2 (-10·5 to 10·9)
Year 6	33·9 (26·8 to 42·4)	38·2 (28·4 to 49·9)	-4·3 (-17·6 to 9·1)
Year 7	33·9 (26·8 to 42·4)	38·2 (28·4 to 49·9)	-4·3 (-17·6 to 9·1)

© 2021 Al-Shahi Salman R et al. *JAMA Neurology*.

	Start antiplatelet therapy (n=268)		Avoid antiplatelet therapy (n=268)		Risk difference	
**All major vascular events (protocol-defined)**						
Year 1	9·2	(6·2 to 13·4)	16·2	(12·3 to 21·2)	-7·0	(-12·6 to -1·4)
Year 2	15·1	(11·3 to 20·1)	24·0	(19·3 to 29·7)	-8·9	(-15·7 to -2·1)
Year 3	21·4	(16·7 to 27·2)	29·2	(24·0 to 35·3)	-7·8	(-15·5 to -0·1)
Year 4	28·0	(22·3 to 34·7)	33·7	(27·9 to 40·3)	-5·7	(-14·5 to 3·1)
Year 5	37·4	(30·1 to 45·8)	36·6	(30·3 to 43·8)	0·7	(-9·7 to 11·2)
Year 6	38·5	(31·0 to 47·1)	43·4	(33·4 to 55·1)	-4·9	(-18·5 to 8·7)
Year 7	38·5	(31·0 to 47·1)	47·5	(35·7 to 60·8)	-8·9	(-24·0 to 6·1)

© 2021 Al-Shahi Salman R et al. *JAMA Neurology*.

eFigure 1. Pre-specified exploratory sub-group analyses of the risk of first recurrent symptomatic intracerebral hemorrhage

Subgroup	Start Antiplatelets Events/N (%)	Avoid Antiplatelets Events/N (%)	HR (95% CI)	P Value*
**Intracerebral haemorrhage location**				0.08
Lobar	17/166 (10.2)	13/166 (7.8)	1.30 (0.63 to 2.68)	
Non-Lobar	5/102 (4.9)	12/102 (11.8)	0.41 (0.14 to 1.17)	
**Time since ICH symptom onset**				0.33
≤ median time from symptom onset	10/129 (7.8)	16/140 (11.4)	0.67 (0.30 to 1.47)	
> median time from symptom onset	12/139 (8.6)	9/128 (7.0)	1.20 (0.51 to 2.86)	
**Antiplatelet drug(s) that the patient's clinician would start**				0.65
Aspirin	12/149 (8.1)	15/149 (10.1)	0.78 (0.36 to 1.66)	
Other	10/119 (8.4)	10/119 (8.4)	1.02 (0.42 to 2.45)	
**Participant's age at randomisation**				0.23
<70 years	3/73 (4.1)	7/73 (9.6)	0.42 (0.11 to 1.61)	
≥70 years	19/195 (9.7)	18/195 (9.2)	1.05 (0.55 to 2.00)	
**Predicted probability of good outcome at 6 months**				0.31
<0.15	4/48 (8.3)	8/51 (15.7)	0.51 (0.15 to 1.69)	
≥0.15	18/220 (8.2)	17/217 (7.8)	1.04 (0.54 to 2.02)	
**History of atrial fibrillation**				0.29
No	13/207 (6.3)	18/195 (9.2)	0.70 (0.34 to 1.43)	
Yes	9/61 (14.8)	7/73 (9.6)	1.36 (0.50 to 3.67)	
**Type of antithrombotic drug regime before ICH**				0.65
Anticoagulant with/without antiplatelet	6/47 (12.8)	6/57 (10.5)	1.11 (0.35 to 3.47)	
Antiplatelet alone	16/221 (7.2)	19/211 (9.0)	0.81 (0.42 to 1.58)	
**Overall**				
Start vs Avoid	22/268 (8.2)	25/268 (9.3)	0.87 (0.49 to 1.55)	

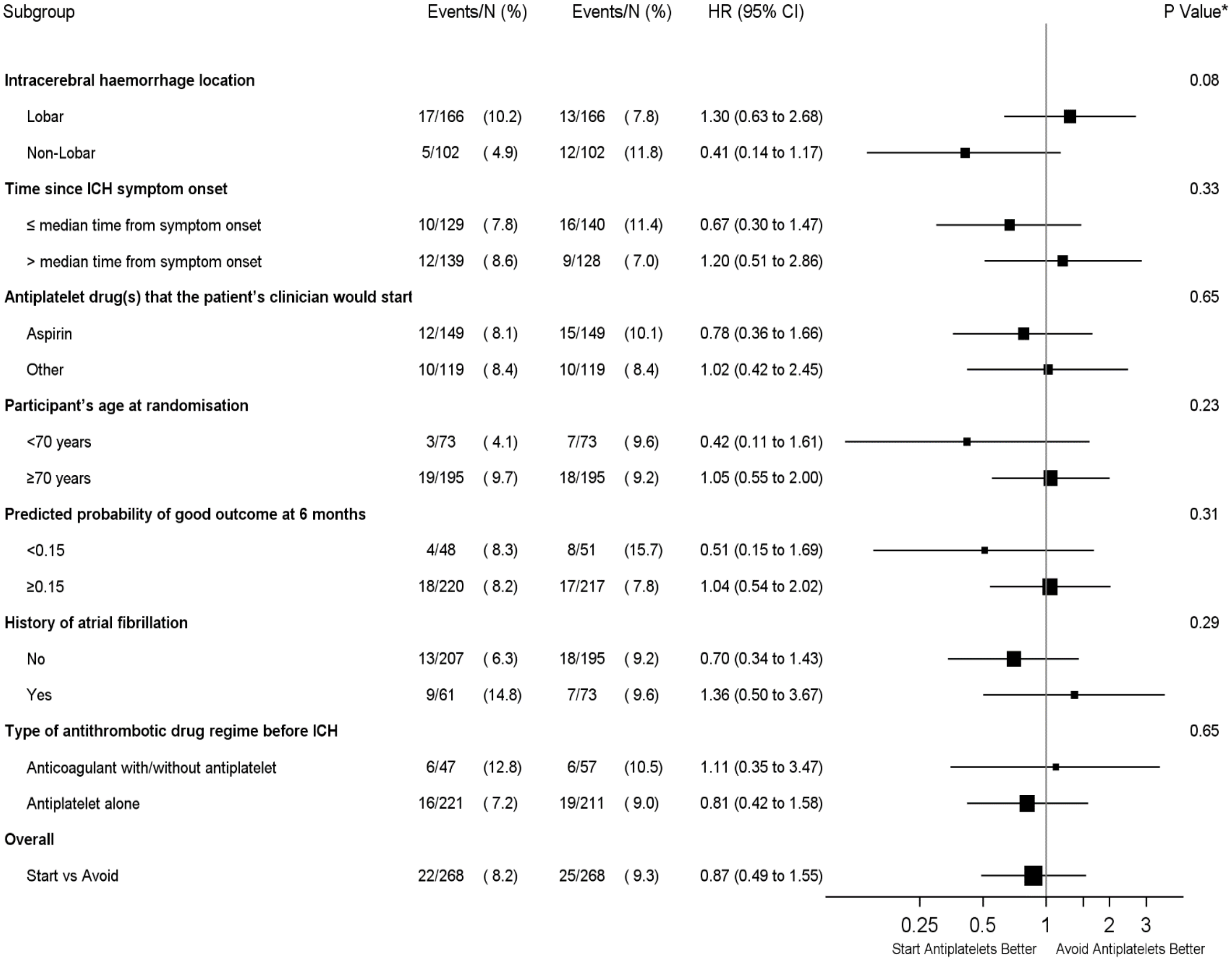

* P-Value is the test of interaction between treatment and each subgroup

© 2021 Al-Shahi Salman R et al. *JAMA Neurology*.

eFigure 2. Kaplan-Meier plot of the risk of the first occurrence of recurrent symptomatic intracerebral hemorrhage, stratified by qualifying intracerebral hemorrhage location

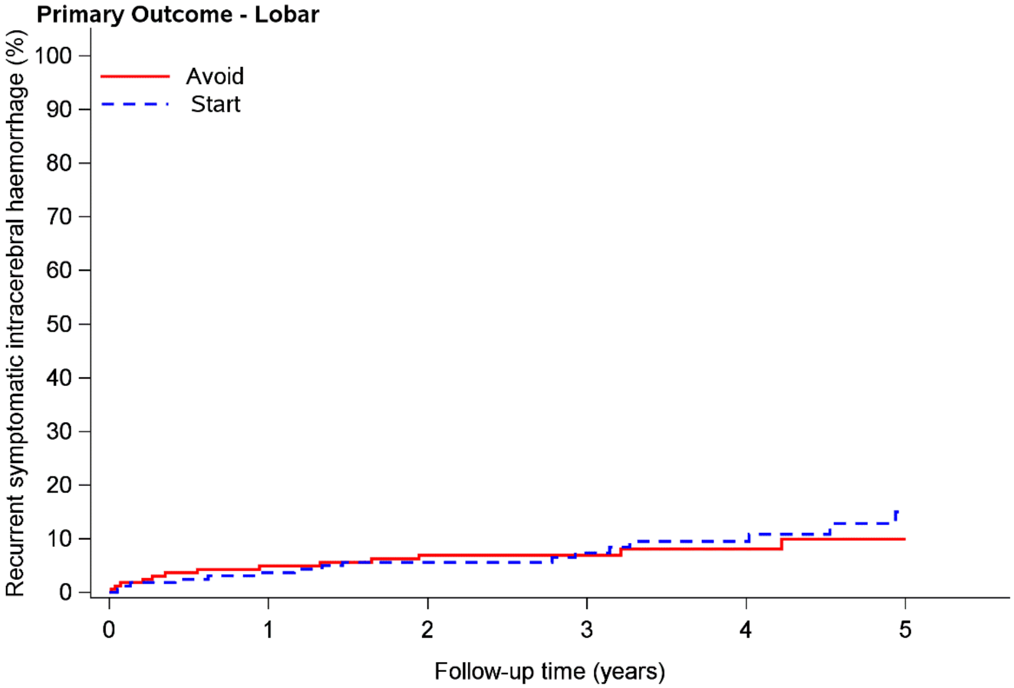

Patients-at-Risk (No. Cumulative Events)

Avoid	166 (0)	147 (8)	128 (11)	98 (11)	57 (12)	34 (13)
Start	166 (0)	151 (6)	136 (9)	101 (11)	70 (13)	39 (16)

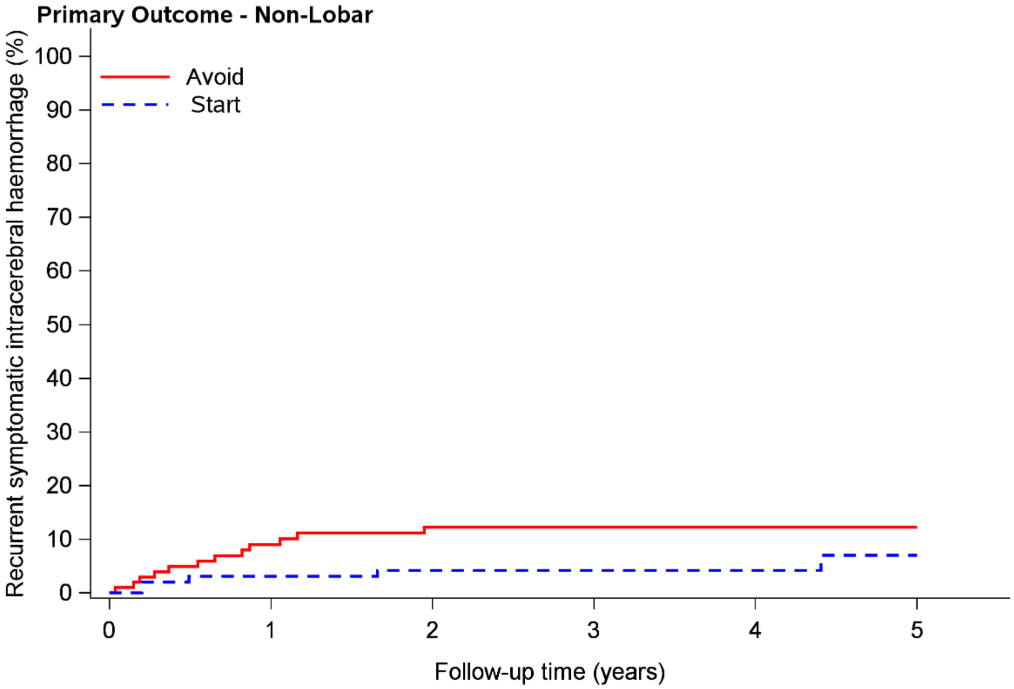

Patients-at-Risk (No. Cumulative Events)

Avoid	102 (0)	86 (9)	77 (12)	61 (12)	42 (12)	33 (12)
Start	102 (0)	88 (3)	75 (4)	60 (4)	41 (4)	26 (5)

© 2021 Al-Shahi Salman R et al. *JAMA Neurology*.

eTable 8. Modified Rankin Scale (mRS) scores rated at randomization by collaborators and during follow-up by participants/carers,[3,4] by treatment allocation

	Start antiplatelet therapy		Avoid antiplatelet therapy	
**At randomization**	**n=268**		**n=269**	
0 (no symptoms)	31	(12%)	26	(10%)
1 (no significant disability)	66	(25%)	59	(22%)
2 (slight disability)	47	(17%)	62	(23%)
3 (moderate disability)	55	(21%)	53	(20%)
4 (moderately severe disability)	57	(21%)	50	(18%)
5 (severe disability)	12	(4%)	19	(7%)
**First annual follow-up (p=0·934)**	**n=268**		**n=268**	
0 (no symptoms)	42	(16%)	43	(16%)
1 (no significant disability)	48	(18%)	42	(16%)
2 (slight disability)	26	(10%)	32	(12%)
3 (moderate disability)	85	(32%)	86	(32%)
4 (moderately severe disability)	23	(9%)	13	(5%)
5 (severe disability)	19	(7%)	27	(10%)
6 (dead)	23	(9%)	23	(9%)
**Second follow-up (p=0·863)**	**n=268**		**n=268**	
0 (no symptoms)	36	(15%)	40	(16%)
1 (no significant disability)	34	(14%)	35	(14%)
2 (slight disability)	23	(9%)	23	(9%)
3 (moderate disability)	76	(31%)	72	(29%)
4 (moderately severe disability)	14	(6%)	11	(5%)
5 (severe disability)	20	(8%)	23	(9%)
6 (dead)	41	(17%)	43	(17%)
**Third annual follow-up (p=0·243)**	**n=232**		**n=233**	
0 (no symptoms)	26	(12%)	31	(15%)
1 (no significant disability)	16	(8%)	25	(12%)
2 (slight disability)	16	(8%)	21	(10%)
3 (moderate disability)	68	(32%)	56	(26%)
4 (moderately severe disability)	11	(5%)	7	(3%)
5 (severe disability)	17	(8%)	16	(8%)
6 (dead)	58	(27%)	57	(27%)
**Fourth annual follow-up (p=0·953)**	**n=178**		**n=180**	
0 (no symptoms)	10	(7%)	11	(7%)
1 (no significant disability)	13	(8%)	17	(11%)
2 (slight disability)	6	(4%)	13	(8%)
3 (moderate disability)	49	(32%)	36	(23%)
4 (moderately severe disability)	10	(7%)	2	(1%)
5 (severe disability)	9	(6%)	14	(9%)
6 (dead)	57	(37%)	63	(40%)

© 2021 Al-Shahi Salman R et al. *JAMA Neurology*.

	Start antiplatelet therapy		Avoid antiplatelet therapy	
**Fifth annual follow-up (p=0·416)**	**n=121**		**n=123**	
0 (no symptoms)	9	(8%)	8	(7%)
1 (no significant disability)	7	(7%)	9	(8%)
2 (slight disability)	5	(5%)	5	(5%)
3 (moderate disability)	22	(20%)	25	(23%)
4 (moderately severe disability)	1	(1%)	3	(3%)
5 (severe disability)	12	(11%)	16	(15%)
6 (dead)	52	(48%)	44	(40%)
**Sixth annual follow-up (p=0·107)**	**n=51**		**n=51**	
0 (no symptoms)	0	(0%)	3	(7%)
1 (no significant disability)	5	(12%)	8	(18%)
2 (slight disability)	0	(0%)	0	(0%)
3 (moderate disability)	7	(16%)	7	(16%)
4 (moderately severe disability)	1	(2%)	0	(0%)
5 (severe disability)	4	(9%)	6	(14%)
6 (dead)	26	(61%)	20	(46%)
**Seventh annual follow-up (p=0·864)**	**n=8**		**n=8**	
0 (no symptoms)	0	(0%)	0	(0%)
1 (no significant disability)	0	(0%)	0	(0%)
2 (slight disability)	1	(17%)	0	(0%)
3 (moderate disability)	0	(0%)	1	(14%)
4 (moderately severe disability)	0	(0%)	0	(0%)
5 (severe disability)	1	(17%)	1	(14%)
6 (dead)	4	(67%)	5	(71%)

The analysis for each year is restricted to participants who were randomized at least the same number of years before the end of recruitment, to avoid including early deaths in the relevant follow-up year when the corresponding surviving recruits would not have had the potential to be assessed.

© 2021 Al-Shahi Salman R et al. *JAMA Neurology*.

eTable 9. Number of serious adverse events, classified by MedDRA preferred term

MedDRA system organ class	Start antiplatelet therapy (n=268)	Avoid antiplatelet therapy (n=269)
Prostate cancer	0	1
Postictal paralysis	0	1
Colitis	0	1
Hydrocholecystitis	0	1
Pelvic fracture	0	1
COVID-19 pneumonia	0	2
Inguinal hernia repair	0	1
Femur fracture	1	0
Cardiac failure congestive	1	0
Strangulated hernia repair	1	0
Overdose	1	0
Fall	3	0
Syncope	1	0
Alcoholic seizure	1	0
Respiratory tract infection	2	0
Acute kidney injury	1	0
Appendicectomy	1	0

References

1. RESTART Collaboration. Effects of antiplatelet therapy after stroke due to intracerebral haemorrhage (RESTART): a randomised, open-label trial. *Lancet* 2019; **393**(10191): 2613-23.
2. Counsell C, Dennis M, McDowall M, Warlow C. Predicting outcome after acute and subacute stroke: development and validation of new prognostic models. *Stroke* 2002; **33**(4): 1041-7.
3. Bruno A, Akinwuntan AE, Lin C, et al. Simplified modified rankin scale questionnaire: reproducibility over the telephone and validation with quality of life. *Stroke* 2011; **42**(8): 2276-9.
4. Bruno A, Shah N, Lin C, et al. Improving modified Rankin Scale assessment with a simplified questionnaire. *Stroke* 2010; **41**(5): 1048-50.

© 2021 Al-Shahi Salman R et al. *JAMA Neurology*.
