# Supplementary material for: Effects of Antiplatelet Therapy After Stroke Caused by Intracerebral Hemorrhage: Extended Follow-up of the RESTART Randomized Clinical Trial
Source: JAMA Neurol. 2021 Sep 3;78(10):1–8. doi: 10.1001/jamaneurol.2021.2956 (PMC8417806; doi:10.1001/jamaneurol.2021.2956)
Supplement: Supplement 2. — Trial Protocol and Statistical Analysis Plan [file jamaneurol-e212956-s002.pdf]

REstart or STop Antithrombotics Randomised Trial

Study Protocol*

**Co-sponsors**	University of Edinburgh & NHS Lothian ACCORD<br>The Queen's Medical Research Institute<br>47 Little France Crescent<br>Edinburgh. EH16 4TJ
**Funder**	British Heart Foundation
**Funding Reference Number**	SP/12/2/29422
**Chief Investigator**	Professor Rustam Al-Shahi Salman
**EudraCT Number**	2012-003190-26 (protocol code RESTART13)
**CTA Number**	01384/0228/001-0001
**REC Reference**	12/SS/0138
**ISRCTN Number**	ISRCTN71907627 (assigned 25/04/2013)
**Version Number and Date**	Version 8.0 19 Sept 2017

* This protocol has been reported in compliance with the SPIRIT 2013 reporting guidelines (Chan AW *et al.* SPIRIT 2013 statement: defining standard protocol items for clinical trials. *Ann Intern Med* 2013;158(3):200-7)

TRIAL PERSONNEL

Trial Co-ordinating Centre

Neurosciences Trials Unit

Centre for Clinical Brain Sciences, First floor, Chancellor's Building, 49 Little France Crescent, Edinburgh. EH16 4SB.

Tel: 0131 242 7994

Fax: 0131 242 7995

Email: RESTART.trial@ed.ac.uk

Web: www.RESTARTtrial.org

Trial Manager

Ms Karen Innes

Centre for Clinical Brain Sciences, First floor, Chancellor's Building, 49 Little France Crescent, Edinburgh. EH16 4SB.

Tel: 0131 465 9610

Fax: 0131 332 5150

Email: Karen.Innes@ed.ac.uk

Chief Investigator

Prof Rustam Al-Shahi Salman

Centre for Clinical Brain Sciences, First floor, Chancellor's Building, 49 Little France Crescent, Edinburgh. EH16 4SB.

Tel: 0131 465 9602

Fax: 0131 537 2944

Email: Rustam.Al-Shahi@ed.ac.uk

Co-sponsor Representative

Dr Fiach O'Mahony
Clinical Research Facilitator
University of Edinburgh
Research Governance
Queens Medical Research Institute
47 Little France Crescent
Edinburgh
EH16 4TJ

Tel: 0131 2429418
Fiach.O'Mahony@ed.ac.uk

Trial Statistician

Professor Gordon D Murray

Centre for Population Health Sciences, University of Edinburgh, Medical School, Teviot Place, Edinburgh. EH8 9AG

Tel: 0131 650 3233

Fax: 0131 650 3224

Email: Gordon.Murray@ed.ac.uk

Statistical Analysis Centre

Edinburgh Clinical Trials Unit (ECTU)

University of Edinburgh, Outpatients Building, Floor Two, Room D36, Western General Hospital, Edinburgh. EH4 2XU

Tel: 0131 537 3814

Fax: 0131 537 3851

Email: Jacqueline.Stephen@ed.ac.uk

Trial Steering Committee Chairperson

Prof Colin N Baigent

Clinical Trial Service Unit, Richard Doll Building, Old Road Campus, Roosevelt Drive, Oxford. OX3 7LF

Tel: 01865 743743

Fax: 01865 743985

Email: Colin.Baigent@ctsu.ox.ac.uk

Data Monitoring Committee Chairperson

Dr John M Bamford

Regional Neurosciences Centre, Department of Neurology, F Floor Martin Wing, Leeds General Infirmary, Great George Street, Leeds. LS1 3EX

Tel: 0113 392 8118

Fax: 0113 392 8070

Email: J.M.Bamford@leeds.ac.uk

PROTOCOL APPROVAL

REstart or STop Antithrombotics Randomised Trial (RESTART)

EudraCT number 2012-003190-26

Signatures

Rustam Al-Shahi Salman<br>**Chief Investigator**	Rustam Al-Shahi Salman<br>Signature	14 Nov 2017<br>Date
Gordon D Murray<br>**Trial Statistician**	Gordon D Murray<br>Signature	9/11/2017<br>Date
Karen Innes<br>**Trial Manager**	Karen Innes<br>Signature	13/11/2017<br>Date
Fiach O'Mahony<br>**Sponsor(s) Representative**	[illegible]<br>Signature	14 Nov 2017<br>Date
**Principal Investigator (PLEASE PRINT)**	Signature	Date

CONTENTS

LIST OF ABBREVIATIONS 7
SUMMARIES 8
1 INTRODUCTION 10
1.1 BACKGROUND 10
1.1.1 Spontaneous intracerebral haemorrhage (ICH) is a devastating disease 10
1.1.2 Patients with spontaneous ICH are affected by vaso-occlusive disease 10
1.1.3 Antiplatelet drugs are effective in the secondary prevention of vaso-occlusive disease 10
1.2 RATIONALE FOR STUDY 11
1.2.1 It is unclear whether antiplatelet drugs modify the risks of recurrent ICH and vaso-occlusive events after spontaneous ICH 11
1.2.2 As a result, it is unclear whether antiplatelet drugs should be started for continued secondary prevention of vaso-occlusive events after spontaneous ICH 11
1.2.3 REstart or STop Antithrombotics Randomised Trial (RESTART) 12
2 STUDY OBJECTIVES 12
2.1 OBJECTIVES 12
2.1.1 Primary objective 12
2.1.2 Secondary objectives 12
3 TRIAL DESIGN 13
3.1 TRIAL FLOWCHART 14
4 STUDY POPULATION 15
4.1 NUMBER OF PARTICIPANTS 15
4.2 INCLUSION CRITERIA 15
4.3 EXCLUSION CRITERIA 15
4.4 CO-ENROLMENT 15
5 PARTICIPANT SELECTION AND ENROLMENT 16
5.1 IDENTIFYING PARTICIPANTS 16
5.1.1 Research staff 16
5.1.2 Timing 16
5.2 CONSENTING PARTICIPANTS 16
5.2.1 Responsibilities 16
5.2.2 Process 17
5.2.3 Documentation 17
5.2.4 Consenting patients who lack capacity to consent themselves 17
5.2.5 New safety information 18
5.3 SCREENING FOR ELIGIBILITY 18
5.4 INELIGIBLE AND NON-RECRUITED PATIENTS 18
6 RANDOMISATION 19
6.1 RANDOMISATION PROCEDURE 19
6.2 RANDOMISATION ALGORITHM 19
6.3 AFTER RANDOMISATION 19
6.4 TREATMENT ALLOCATION 19
6.5 BLINDING 19

7 PREMATURE WITHDRAWAL OF PARTICIPANTS 20
8 CROSSOVERS 20
9 INVESTIGATIONAL MEDICINAL PRODUCT 20
9.1 INTERVENTION 20
9.1.1 COMPARATOR 20
9.2 PARTICIPANT ADHERENCE 20
9.3 OVERDOSE 21
9.4 OTHER MEDICATIONS 21
9.4.1 Non-Investigational Medicinal Products 21
9.4.2 Permitted medications 21
9.4.3 Prohibited medications 21
9.5 OUTCOME MEASURES 22
9.5.1 Primary outcome 22
9.5.2 Secondary outcomes 22
10 STUDY ASSESSMENTS AND DATA COLLECTION 23
10.1 STUDY ASSESSMENT SCHEDULE 23
10.1.1 Randomisation 23
10.1.2 Discharge 24
10.1.3 Long term follow-up 24
10.1.4 Outcome event adjudication 25
10.1.5 Long-term follow-up after the study period 25
10.2 SAFETY ASSESSMENTS 27
10.2.1 Types of safety assessment 27
11 STATISTICS AND DATA ANALYSIS 27
11.1 SAMPLE SIZE CALCULATION 27
11.2 PROPOSED ANALYSES 28
11.2.1 Primary analysis 28
11.2.2 Secondary analyses 28
11.2.3 Sub-group analyses 29
11.2.4 Brain MRI sub-study 29
12 ADVERSE EVENTS 29
12.1 DEFINITIONS 30
12.1.1 Adverse event 30
12.1.2 Adverse reaction 30
12.1.3 Serious adverse event 30
12.1.4 Suspected Unexpected Serious Adverse Reaction 30
12.2 ASSESSMENT OF ADVERSE EVENTS 30
12.2.1 Assessment of Seriousness 30
12.2.2 Assessment of Causality 30
12.2.3 Assessment of Severity 31
12.2.4 Assessment of Expectedness 31
12.2.5 Expected adverse reactions to aspirin, dipyridamole and clopidogrel 31
12.2.6 Expected non-fatal complications of ICH 31
12.3 DETECTING ADVERSE EVENTS 32
12.4 REPORTING ADVERSE EVENTS 32
12.5 ADVERSE EVENT REPORTING IN RESTART 33
12.5.1 Investigators should report outcomes to the Trial Co-ordinating Centre 33

12.5.2 Investigators must report SAEs and SUSARs to the Trial sponsor 33
12.5.3 Investigators should not report other events to the Trial Co-ordinating Centre or sponsor 34
12.6 REPORTING OF SAES AND SUSARS 34
12.7 REGULATORY REPORTING REQUIREMENTS 35
13 PREGNANCY 35
14 TRIAL MANAGEMENT AND OVERSIGHT ARRANGEMENTS 35
14.1 ROLE OF FUNDER AND SPONSOR 35
14.2 TRIAL MANAGEMENT GROUP 35
14.3 TRIAL CO-ORDINATING CENTRE 35
14.4 TRIAL STEERING COMMITTEE 36
14.5 DATA MONITORING COMMITTEE 36
14.6 INSPECTION OF RECORDS 36
14.7 RISK ASSESSMENT 36
14.8 STUDY MONITORING AND AUDIT 36
14.8.1 Archiving of site data 37
14.8.2 Archiving of central data 37
15 GOOD CLINICAL PRACTICE 37
15.1 ETHICAL CONDUCT 37
15.2 REGULATORY COMPLIANCE 37
15.3 PRINCIPAL INVESTIGATOR RESPONSIBILITIES 37
15.3.1 Confirming participant eligibility and informed consent 37
15.3.2 Study Site Staff 38
15.3.3 Data Recording 38
15.3.4 Investigator Documentation 38
15.3.5 GCP Training 38
15.3.6 Confidentiality 38
15.3.7 Data Protection 38
15.3.8 Follow-up 38
16 STUDY CONDUCT RESPONSIBILITIES 39
16.1 PROTOCOL AMENDMENTS 39
16.2 PROTOCOL VIOLATIONS AND DEVIATIONS 39
16.3 SERIOUS BREACH REQUIREMENTS 40
16.4 END OF STUDY 40
16.5 CONTINUATION OF DRUG FOLLOWING THE END OF STUDY 40
16.6 INSURANCE AND INDEMNITY 41
17 REPORTING, PUBLICATIONS AND NOTIFICATION OF RESULTS 41
17.1 AUTHORSHIP POLICY 41
17.2 PUBLICATION 41

APPENDIX 1: COMMITTEES 43
APPENDIX 2: REFERENCES 45

LIST OF ABBREVIATIONS

**ACCORD**	Academic and Clinical Central Office for Research & Development
**AE**	Adverse event
**AR**	Adverse Reaction
**BP**	Blood pressure
**CI**	Chief Investigator
**CPRD**	Clinical Practice Research Datalink
**CRF**	Case Report Form
**CRN**	Clinical Research Network
**CTIMP**	Clinical Trial of an Investigational Medicinal Product
**DMC**	Data Monitoring Committee
**GCP**	Good Clinical Practice
**GP**	General practitioner
**HSCIC**	Health and Social Care Information centre
**ICF**	Informed consent form
**ICH**	Intracerebral haemorrhage
**IMP**	Investigational Medicinal Product
**ISF**	Investigator Site File
**MRI**	Magnetic Resonance Imaging
**NHS**	National Health Service
**PI**	Principal Investigator (at a local site)
**PIL**	Patient Information Leaflet
**RCT**	Randomised controlled trial
**REC**	Research Ethics Committee
**R&D**	Research and Development department
**SAE**	Serious Adverse Event
**SAR**	Serious Adverse Reaction
**SPC**	Summary of product characteristics
**SUSAR**	Suspected Unexpected Serious Adverse Reaction
**TCC**	Trial Co-ordinating Centre
**TMF**	Trial Master File
**TSC**	Trial Steering Committee

SUMMARIES

Professional summary

**Primary research question**	For adults surviving spontaneous (non-traumatic) intracerebral haemorrhage (ICH) who had taken an antithrombotic (i.e. anticoagulant or antiplatelet) drug for the prevention of vaso-occlusive disease before the ICH, does a policy of starting antiplatelet drugs result in a beneficial net reduction of all serious vascular events compared with a policy of avoiding antiplatelet drugs?
**Trial design**	Investigator-led, multicentre, randomised, open, assessor-blind, parallel group, clinical trial of investigational medicinal product (CTIMP) prescribing strategies.
**Setting**	UK National Health Service (NHS) secondary care (inpatient and outpatient services in stroke, neurology and neurosurgery) and primary care.
**Eligibility criteria**	**Inclusion:** Patient age ≥18 years. Spontaneous primary or secondary ICH. Patient had taken antithrombotic drug(s) for the prevention of vaso-occlusive disease before ICH onset. Randomisation more than 24 hours after ICH onset. Patient and their doctor are uncertain about whether to start or avoid antiplatelet drugs. Patient is registered with a general practitioner (GP). Brain imaging that first diagnosed the ICH is available. Participant or representative consent.<br><br>**Exclusion:** ICH due to preceding trauma or haemorrhagic transformation of ischaemic stroke. Patient is taking an anticoagulant drug following ICH. Patient is pregnant, breastfeeding, or of childbearing age and not taking contraception. Patient and carer unable to understand spoken or written English.<br><br>**Brain magnetic resonance imaging (MRI) sub-study:** MRI done after ICH but before randomisation**.** No claustrophobia. MRI not contraindicated.
**Randomisation**	Central, web-based randomisation system using a minimisation algorithm, with 1:1 treatment allocation to which central research staff are masked.
**Interventions**	Start antiplatelet drug(s) (one or more of aspirin, clopidogrel, or dipyridamole, chosen by patient's physician pre-randomisation) *vs.* avoid antiplatelet drug(s).
**Outcome measures**	**Primary outcome:** recurrent symptomatic ICH<br><br>**Secondary outcomes:** symptomatic haemorrhagic events; symptomatic vaso-occlusive events; symptomatic stroke of uncertain type; other fatal events; modified Rankin Scale score; adherence to antiplatelet drug(s).
**Follow up**	*Central:* annual postal or telephone questionnaires to participants and their GPs. *Local:* medical records and any brain imaging relating to outcomes. *Administrative data:* Flagging and the GP Clinical Practice Research Datalink (CPRD).
**Power**	Given that the annual recurrence rate of ICH may be 1.8-7.4% and there may be a 1-4-fold relative increase in this risk on antiplatelet drugs, this trial will have 90% power to detect a doubling of an annual ICH rate of 4.5% or 93% power to detect a quadrupling of an annual rate of 1% over two years at the 5% level. This trial will also provide adequately precise estimates of the rates of all serious vascular events to inform the design of a trial with the power to assess net clinical benefit.
**Statistical methods**	Hazard ratio after randomisation, adjusted for baseline covariates included in the minimisation algorithm.
**Sample size**	Recruitment began on 22 May 2013 and the target sample size is at least 720 participants in the main trial (at least 550 in the MRI sub-study).

Lay summary

More than one third of the adults with a stroke due to bleeding into the brain – known as brain haemorrhage – are taking drugs to prevent clotting when they have a brain haemorrhage.

These patients had previously suffered illnesses like angina, heart attack, or stroke due to blood vessel blockage, which is why they are treated with drugs to prevent further clots occurring. These drugs are usually stopped when the brain haemorrhage occurs.

But when patients recover from brain haemorrhage, they and their doctors are often uncertain about whether to restart these drugs to prevent further clots occurring, or whether to avoid them in case they increase the risk of brain haemorrhage happening again.

In this preliminary study of 720 such people who survive a brain haemorrhage, we will study the potentially beneficial effects of three antiplatelet drugs (one or more of aspirin, clopidogrel, or dipyridamole, chosen by the patient's physician) on the risks of heart attack, stroke and other clotting problems as well as their effect on the risk of a brain haemorrhage happening again.

This information will help us to decide whether antiplatelet drugs are a promising treatment. If they are, we will recruit a much larger number of patients so that we can determine really reliably whether the beneficial effects of antiplatelet drugs on the risk of clotting outweigh any risks of a repeat brain haemorrhage for such people.

1 INTRODUCTION

1.1 BACKGROUND

1.1.1 Spontaneous intracerebral haemorrhage (ICH) is a devastating disease

Intracranial haemorrhage occurs in any of the compartments within the cranium (figure).[1] Blood may be restricted to one compartment, but sometimes it may extend from one compartment to another. 'Traumatic' intracranial haemorrhage occurs after head injury, whilst '**spontaneous**' intracranial haemorrhage occurs without preceding head injury (although head injury may be a consequence of it). A patient's history, examination findings, and radiographic appearances (of the intracranial haemorrhage, the rest of the brain, the skull, and scalp soft tissue) are usually used to distinguish traumatic from spontaneous intracranial haemorrhage.

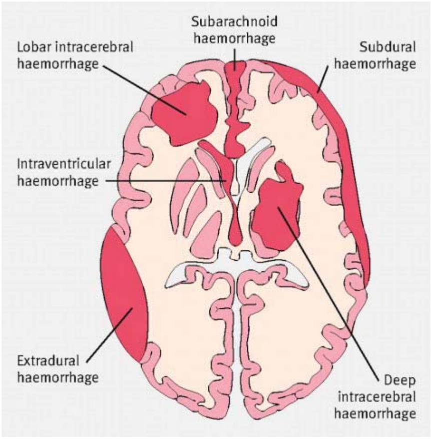

Intracranial haemorrhage types[1]

Intracerebral haemorrhage (ICH) only refers to bleeding within the brain substance (figure). Traditionally only spontaneous ICH and spontaneous subarachnoid haemorrhage (figure) are classified as 'haemorrhagic stroke'.

This trial is restricted to adults with stroke due to spontaneous ICH, which affects ~10,000 adults in the UK each year, and is more common than the other types of intracranial haemorrhage. Within one month of ICH ~40% of patients die and more than half the survivors are dependant.[2]

1.1.2 Patients with spontaneous ICH are affected by vaso-occlusive disease

Patients with spontaneous ICH have past histories of hypertension (47-66%), smoking (41%), and diabetes mellitus (11-14%).[3,4] These risk factors also contribute to the occurrence of other diseases prior to ICH, such as ischaemic stroke (14-23%), ischaemic heart disease (8-21%), and atrial fibrillation (11-14%).[3,4] These risk factors and diseases may also cause vaso-occlusive events after ICH, which – overall – appear to occur with a similar frequency to recurrent ICH (see table below), although the annual risk of recurrent ICH after lobar ICH appears higher (~14%).[5,6] However, little is known about these risks in the specific sub-group of people with ICH and a past history of vaso-occlusive disease(s), although it is likely that their future risks of vaso-occlusive events are higher than this.

Annual absolute risk estimates of vascular events for all patients with ICH who survive for at least one month[5,7-22]

Vascular death	Myocardial infarction	Ischaemic stroke	ICH
2% to 3%	0.2% to 1%	1.3% to 3%	1.8% to 7.4%

1.1.3 Antiplatelet drugs are effective in the secondary prevention of vaso-occlusive disease

Among individuals at high risk because of a prior vaso-occlusive event, aspirin provides statistically and clinically significant absolute reductions in all serious vascular events from 8.2% to 6.7% per year, in all stroke from 2.5% to 2.1% per year, and in coronary events from 5.3% to 4.3% per year, despite a non-significant increase in the risk of intracranial haemorrhage.[23] Antiplatelet drugs also seem to be beneficial for preventing vaso-occlusive events in patients with atrial fibrillation and no past history of

vaso-occlusive events, without a detectable increase the risk of extracranial or intracranial haemorrhage.[24] However, patients with spontaneous ICH were not included in the trials contributing to these analyses,[24,25] but it is likely that the benefits of secondary prevention with antiplatelet drugs would apply after ICH (although whether they are outweighed by the risk of recurrent ICH is unknown).

1.2 RATIONALE FOR STUDY

1.2.1 It is unclear whether antiplatelet drugs modify the risks of recurrent ICH and vaso-occlusive events after spontaneous ICH

For patients who develop spontaneous ICH while taking antithrombotic drugs for the prevention of vaso-occlusive disease (for example, patients taking antiplatelet drugs after myocardial infarction or patients taking an anticoagulant for lone atrial fibrillation), it has long been unclear whether survivors of ICH should start antiplatelet drugs for continued secondary prevention of vaso-occlusive disease or avoid antiplatelet drugs in case it increases the risk of extracranial and intracranial haemorrhage.[26] In a systematic review of Medline, Embase, the Cochrane Stroke Group Trials Register and the Stroke Center Trials Registry in August 2011, the Chief Investigator found no randomised controlled trials (RCTs) addressing this dilemma. There are three observational studies addressing the safety of long-term antiplatelet drugs for secondary prevention after spontaneous ICH.[27-29] One study of 417 survivors of spontaneous ICH in any brain location in Scotland found neither an increase in the subsequent risk of recurrent ICH associated with aspirin nor a beneficial reduction of vaso-occlusive events.[29] Another study of 440 survivors of first spontaneous ICH in any brain location in China did not find an increase in the subsequent risk of recurrent ICH associated with aspirin, but did find its prescription was associated with a beneficial reduction in the composite endpoint of recurrent ICH, ischaemic stroke or acute coronary syndrome during follow-up in the sub-group of patients with indications for aspirin.[28] A third study of 104 survivors of lobar ICH found an increase in the subsequent risk of recurrent ICH associated with aspirin in multivariable analyses.[27] This study also assessed associations with brain microbleeds on magnetic resonance imaging (MRI) – which appear to be a biomarker of microangiopathies[30,31] and an increased risk of recurrent ICH[30,32] – and found the hazard associated with aspirin increased with increasing numbers of brain microbleeds.[27] The sample sizes of these studies were not large and allocation to aspirin was not randomised, so chance, bias and confounding might have influenced their results.

1.2.2 As a result, it is unclear whether antiplatelet drugs should be started for continued secondary prevention of vaso-occlusive events after spontaneous ICH

Because of the lack of RCTs and the findings of the small observational studies discussed in section 1.2.1 above, guidelines have endorsed starting or avoiding antiplatelet drugs in their recommendations about the management of ICH survivors who had been on antithrombotic drugs for prevention of vaso-occlusive diseases prior to ICH.[33-35] Consequently, either starting or avoiding antiplatelet drugs for the prevention of vaso-occlusive disease after spontaneous ICH **are forms of standard clinical care**.

We confirmed clinical equipoise in October 2011 by sending a survey to UK collaborators in the Third International Stroke Trial (IST3) and the Clots in Legs Or sTockings after Stroke (CLOTS) trial, as well as to all UK Stroke Research Network regional leads: 136 consultant stroke physicians and neurologists at 97 different hospitals in the UK indicated that, because of their individual uncertainty, they would randomise patients in a RCT that compared policies of starting antiplatelet drugs versus avoiding antiplatelet drugs for spontaneous ICH survivors who had been on antithrombotic drugs until the time of ICH. Importantly, between two-thirds and three-quarters of respondents were uncertain about

management decisions after spontaneous ICH for any sort of antiplatelet drug, any location of ICH in the brain, and any pre-ICH co-morbidities that had been indications for using antiplatelet drugs.

1.2.3 REstart or STop Antithrombotics Randomised Trial (RESTART)

Because the benefits of antiplatelet drugs for the prevention of vaso-occlusive disease are likely to continue to apply after ICH (1.1.3), because the effect of antiplatelet drugs on the risk of recurrent ICH is unknown (1.2.1), and because starting antiplatelet drugs after ICH occurs in standard clinical practice (1.2.2), it is reasonable to consider starting antiplatelet drugs. However, because the published observational studies have not shown dramatic effects[36] of antiplatelet drugs on the risk of recurrent ICH (see 1.2.1), a RCT like RESTART is needed to address the uncertainty about whether to start or avoid antiplatelet drugs in ICH survivors. A similarly-designed RCT has proven feasible and acceptable for patients who had been taking low dose aspirin before peptic ulcer bleeding.[37]

2 STUDY OBJECTIVES

2.1 OBJECTIVES

2.1.1 Primary objective

Ultimately, we want to determine in a phase III RCT whether antiplatelet drugs are *beneficial* for patients after spontaneous ICH because the gains from prevention of vaso-occlusive disease outweigh the risks of intracranial and extracranial haemorrhage. The **primary objective** of the pilot phase is to estimate the relative and absolute effects of antiplatelet drugs on the risk of recurrent symptomatic ICH associated with a policy of starting antiplatelet drugs after the acute phase of spontaneous ICH.

Based on the likely frequency of vaso-occlusive events and the likely size of the reduction in their frequency associated with antiplatelet drugs in ICH survivors with established vaso-occlusive disease, a doubling of the risk of recurrent symptomatic ICH associated with antiplatelet drugs after spontaneous ICH might outweigh the benefits of secondary prevention; this magnitude of risk would be consistent with observational studies after ICH[25,27-29] and the known risks of antiplatelet drugs.[38,39] Based on this outcome, together with the overall event rates for all vaso-occlusive outcomes (including myocardial infarction, ischaemic stroke and vascular death) as this RCT proceeds, the Trial Steering Committee (TSC) will decide whether to proceed with a much larger trial designed to determine whether the benefits of antiplatelet drugs exceed the haemorrhagic risks among survivors of ICH.

2.1.2 Secondary objectives

Ultimately, we aim to determine whether the presence of brain microbleeds on MRI modifies the effect of antiplatelet drugs on the risk of recurrent ICH, In the pilot phase we will see whether ICH recurrence is more common among those with more microbleeds, and the extent to which this is so, allowing us to better formulate a hypothesis for a larger main study.

3 TRIAL DESIGN

The REstart or STop Antithrombotics Randomised Trial (RESTART) is an investigator-led, multicentre, randomised, open, assessor-blind, parallel group clinical trial of investigational medicinal product (**CTIMP**) prescribing strategies in standard care at multiple hospitals in the National Health Service (NHS) in the United Kingdom (UK).

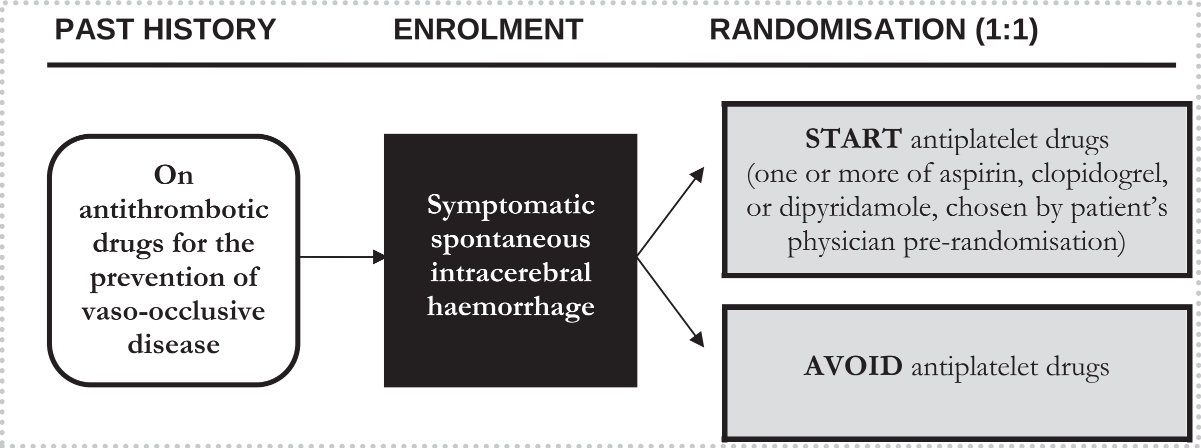

The **participant** eligibility criteria specifically identify adults with symptomatic spontaneous ICH who had taken antithrombotic drugs for the prevention of vaso-occlusive disease before the ICH. Randomisation occurs if a participant and their doctor are uncertain about whether to start or avoid antiplatelet drugs (one or more of aspirin, clopidogrel, or dipyridamole) at least 24 hours after ICH symptom onset. The **intervention** is a pragmatic policy of starting antiplatelet drugs (one or more of aspirin, clopidogrel, or dipyridamole, chosen by patient's physician before randomisation). The **comparator** is a policy of avoiding antiplatelet drugs. The primary **outcome** is fatal or non-fatal radiographically- or pathologically-proven recurrent symptomatic ICH, and secondary outcomes include all serious vascular events as well as annual ratings of participant function.

The TSC will review the target sample size of 720 participants and adjust it if necessary based on accruing data on: the number of primary outcome events, completeness of follow up, and the enrolment into specific pre-specified subgroups. The TSC will remain blinded to outcome according to treatment allocation, unless the Data Monitoring Committee (DMC) recommends stopping the trial. Information about the overall rates of all serious vascular events (for both arms combined) will be used together with reasonable assumptions about the effects of antiplatelet drugs on such events in order to assess the plausibility of a net benefit emerging in a larger main study.

We will perform a **brain MRI sub-study to test for an interaction between the presence of brain microbleeds** (which are diagnostic and prognostic radiographic biomarkers) and the effects of antiplatelet drugs and explore whether there is a trend in the risk of recurrent ICH with increasing numbers of microbleeds.

We will also study trial methodology by performing an opt-in, cluster-randomised, stepped wedge trial at a sub-group of RESTART sites, to assess the effects of an intervention to manage the performance of sites to help them fulfil the recruitment targets they set at their site initiation visit.

3.1 TRIAL FLOWCHART

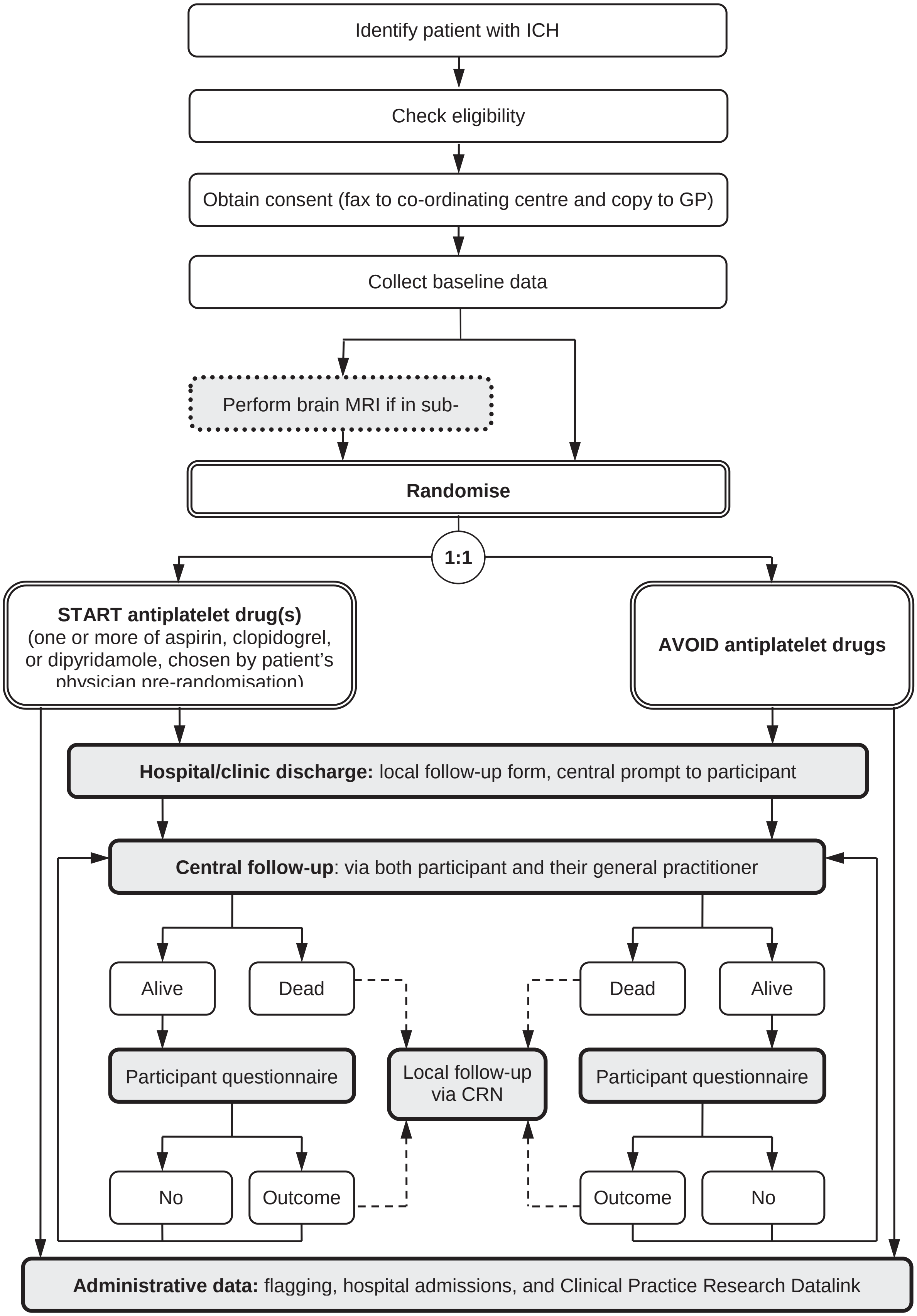

4 STUDY POPULATION

4.1 NUMBER OF PARTICIPANTS

We aim to enrol at least 720 participants in RESTART, at least 550 of whom we aim to recruit to the MRI sub-study.

4.2 INCLUSION CRITERIA

- Patient age ≥18 years.
- Spontaneous ICH
  - not attributable to preceding head injury, on the basis of:
    - a history from the patient/witness of spontaneous symptom onset without *preceding* head trauma (head trauma occurring *subsequent* to ICH symptom onset is permissible)
    - brain imaging appearances consistent with spontaneous ICH (which may be accompanied by the brain/bone/soft tissue appearances of trauma occurring subsequently)
  - either 'secondary' to an underlying structural cause (e.g. aneurysm, tumour, arteriovenous malformation, or intracranial venous thrombosis), or 'primary' (if the investigator either does not suspect an underlying structural cause, or it is not detected by further radiographic investigation).[1,40]
- Patient had taken an antithrombotic (i.e. anticoagulant or antiplatelet) drug for the prevention of vaso-occlusive disease for any length of time before the onset of the qualifying ICH.
- Patient is at least 24 hours after ICH symptom onset.
- Patient and their doctor are both uncertain about whether to start or avoid antiplatelet drugs.
- Patient is registered with a general practitioner (GP).
- Brain imaging study that first diagnosed the qualifying ICH is available.
- Consent to randomisation from the patient (or personal / legal / professional representative if the patient does not have mental capacity).
- *If eligible for the brain MRI sub-study*, the MRI must be performed after the ICH but before randomisation.

4.3 EXCLUSION CRITERIA

- ICH due to head injury, in the opinion of the investigator.
- ICH due to haemorrhagic transformation of an ischaemic stroke, in the opinion of the investigator.
- Patient is taking an anticoagulant drug following ICH
- Patient is pregnant, breastfeeding, or of childbearing age and not taking contraception.
- Patient and carer unable to understand spoken or written English (local translator is not available).
- *Patients are ineligible for the brain MRI sub-study* if they are claustrophobic or they have a contraindication to MRI.

4.4 CO-ENROLMENT

Inclusion in another research study, including another RCT but not including a Phase I or first-time-into-human study, does not preclude participation in RESTART as long as: participants are not overburdened; their inclusion would not confound RESTART's results or complicate attribution of serious adverse events and outcomes; and co-enrolment has been agreed with the Chief Investigators of all studies involved in co-enrolment. Arrangements for co-enrolment with another CTIMP will be bound by a written agreement between the RESTART Chief Investigator and Co-Sponsors and the Chief Investigator and Sponsor(s) of the other CTIMP(s). This agreement will include: safety reporting

measures if required; a minimum wash-out period between last dose in one study and first dose in another; a statement to indicate that the chairs of the TSC/DMC from each study that they have no objections to the proposals for co-enrolment; and a statement that arrangements for attribution of liability for co-enrolled participants have been put in place. Research staff should obtain permission to enrol patients who are participants in other CTIMPs from the RESTART Chief Investigator via the TCC or by email and a record of co-enrolled participants should be maintained. Participants in TICH2 (www.tich-2.org) may be co-enrolled in RESTART if at least 21 days have elapsed after enrolment in TICH2 and the terms of the co-enrolment agreement are upheld.

5 PARTICIPANT SELECTION AND ENROLMENT

5.1 IDENTIFYING PARTICIPANTS

5.1.1 Research staff

Research staff who may identify potential participants for RESTART should be part of, or affiliated to, the clinical team responsible for the patient's care, including **doctors** (stroke physicians, geriatricians, neurologists, and neurosurgeons) and **clinical or research nurses**. Research staff will be based at NHS hospital sites where local resources would facilitate patient identification and eligibility assessment. Potentially eligible patients may be identified in the everyday clinical practice of research staff, or referred to them for assessment of eligibility having been identified elsewhere (e.g. by clinical staff who are not research staff in secondary or primary care). A list of participating sites may be obtained from the TCC. At local sites, in this protocol we refer to:

- **principal investigators** (the lead doctor at a research site)
- **delegated physicians** (doctors with delegated responsibilities for e.g. eligibility assessment and adverse event reporting)
- **local coordinator** (the lead member of research staff at a research site)
- **local research staff** (who support the principal investigator and delegated physicians)

5.1.2 Timing

These research staff should identify patients at least 24 hours after ICH symptom onset and **at a time when uncertainty arises** about whether to avoid or start antiplatelet drugs. There is **no specific time window** for identifying participants, so they may be recruited during their hospital admission for the qualifying ICH or at a later stage in an outpatient clinic. Research staff should approach the patient, or their personal / legal / professional representative (if the patient lacks mental capacity to consent for themselves), to ascertain their interest in participating in RESTART and permission to pass their details on to any research staff involved.

5.2 CONSENTING PARTICIPANTS

5.2.1 Responsibilities

The Principal Investigator (PI), or physician with delegated responsibility, is responsible for confirming eligibility, ensuring informed consent is obtained and that the informed consent form (ICF) is completed, signed and dated by all parties and faxed to the RESTART trial office prior to randomisation and any protocol-specific procedures being carried out. Local research staff should follow the laws that govern consent procedures in their jurisdiction, and in particular those governing incapacitated adults and their involvement in research (see 5.2.4).

5.2.2 Process

Patients or their representatives must receive adequate oral and written information, so research staff should provide the appropriate Participant Information Leaflet (PIL) and ICF. The oral explanation to the patient should be performed by the PI or delegated physician or research staff member, and must cover all the elements specified in the PIL and ICF. The patient must be given every opportunity to clarify any points they do not understand and, if necessary, ask for more information. The patient will be told that they may withdraw their consent to participate at any time without loss of benefits to which they otherwise would be entitled. The patient or their representative will be given as much time as they need to consider giving consent for the trial. Brain MRI must be performed before randomisation.

5.2.3 Documentation

The PI, delegated physician or research staff member and the participant should sign and date the ICF to confirm that consent has been obtained. Written informed consent should always be sought from the participant where possible. If this is not possible because the participant cannot write, the randomising clinician or nurse can gain witnessed verbal consent. The participant or their personal legal representative should receive a copy of the ICF, a copy should be filed in the patient's medical records, a copy should be faxed to the trial co-ordinating centre (TCC) and the original ICF should be filed in the investigator site file (ISF) along with the PIL used and the randomisation form. Full details of the consent process should also be recorded in the participant's medical records for any future source data verification, using the RESTART sticker provided in the trial manual, which mentions the date of consent, that the participant received the PIL, who obtained consent and signed and dated confirmation that the patient was eligible for enrolment.

5.2.3.1 Principal Investigator responsibilities to the participant

The participant should receive a pack including a PIL, a copy of the completed ICF, and a participant diary and fridge magnet which has contact details for the TCC and prompts the recording and reporting of adverse events etc.

5.2.3.2 Principal Investigator responsibilities to the trial

A copy of the ICF should be sent to the TCC via fax or secure email, ideally before randomisation or soon afterwards, as this has to be provided to the GP along with the notification that the patient has been recruited into the trial. The trial management system will prompt local research staff to do so via email &/or fax if the ICF has not been received.

5.2.4 Consenting patients who lack capacity to consent themselves

If a patient lacks capacity to consent for themselves then a personal / legal / professional representative may provide consent on the patient's behalf. The table overleaf specifies the hierarchy that should be applied according to location in the UK.

Hierarchy of informed consent for an adult with mental incapacity	
England, Wales and Northern Ireland	*Scotland*
1. Personal legal representative<br>A person not connected with the conduct of the trial who is:<br>(a) suitable to act as the legal representative by virtue of their relationship with the adult<br>*and*<br>(b) available and willing to do so.<br><br>*2. Nominated legal representative*<br>Doctor primarily responsible for the incapacitated adult's treatment or a person nominated by their healthcare provider.	*1. Personal legal representative*<br>Any guardian or welfare attorney who has power to consent to the adult's participation in research. If there is no such person, then …<br>*2. Nearest relative*<br>Adult's nearest relative as defined in section 254 of the Mental Health (Care and Treatment) (Scotland) Act 2003. If it is not reasonably practicable to contact 1 or 2, then…<br>*3. Doctor primarily responsible for the incapacitated adult's treatment or a person nominated by their healthcare provider.*

5.2.4.1 Re-consenting patients who regain capacity

If a participant regains capacity during any hospital stay, the participant should be informed about their enrolment in the trial and fully informed consent should be obtained from the participant. If the participant regains mental capacity after hospital discharge but during the follow up we will not attempt to re-consent at that stage because the nature of the follow up will make it impractical to know whether the participant has regained capacity. The participant who does regain capacity may withdraw their consent to participate at any time, by calling the trial office, without loss of benefits to which they otherwise would be entitled (see section 7).

5.2.5 New safety information

If the DMC decides that any new safety information results in significant changes in the risk-benefit analysis, the PIL and ICF will be reviewed and updated accordingly. All participants who are enrolled in RESTART will be informed of the updated information and given a revised copy of the PIL/ICF in order to confirm their wish to continue in the trial.

5.3 SCREENING FOR ELIGIBILITY

Members of the clinical team, including research nurses may screen records of hospital admissions (e.g. stored in the electronic patient records held by their hospital or national stroke audits) and outpatient attendances at neurovascular clinics of patients they currently or previously cared for, to determine if patients meet the inclusion criteria. Early screening may permit ample time for the patients and/or their families to consider the trial materials, ask questions and still be recruited prior to hospital discharge.

5.4 INELIGIBLE AND NON-RECRUITED PATIENTS

Screening logs are not part of the RESTART data collection process. Although complete screening logs may provide information about the generalisability of a trial's results, we have already assessed the characteristics of eligible patients on a community basis. Furthermore, the completion of study specific screening logs involves substantial effort for participating sites, and may divert time from the key tasks of treating and recruiting patients to protocol.

6 RANDOMISATION

6.1 RANDOMISATION PROCEDURE

Having obtained consent, the researcher collects the baseline data necessary to complete a randomisation form, including confirmation that the imaging that diagnosed the qualifying ICH is available and the availability of the brain MRI if the patient is in the sub-study. The researcher then enters the participant's baseline data (including the indication for pre-ICH antithrombotic drugs and the reason for uncertainty about whether or not to start antiplatelet drugs) into a computerised central randomisation service by means of a secure 24/7 web interface or a telephone call to the trial office during office hours (if the web interface is not operational). After the computer program has checked these baseline data for completeness and consistency it allocates that patient a unique study identification number and informs the researcher about the participant's allocation to starting or avoiding antiplatelet drugs.

6.2 RANDOMISATION ALGORITHM

To avoid predictable alternation of treatment allocation (and potential consequent loss of allocation concealment) the minimisation algorithm will randomly allocate the first participant with a probability of 0.5 to one arm of the trial. But the randomisation algorithm for each subsequent participant involves adaptive stratification (i.e. minimisation) and allocates them with a probability of 0.8 to the group which minimises differences between the two arms of the trial with respect to five variables collected by research staff at baseline:

- Qualifying ICH location (lobar *versus* non-lobar, based on local investigator's interpretation of scan)
- Time since ICH symptom onset (0-6 days, 7-30 days > 30 days)
- Antiplatelet drug(s) that the patient's physician would start if allocated (aspirin alone *versus* other antiplatelet regimen [including combination treatment])
- Participant age at randomisation (<70 years *versus* 70 years or older)
- Predicted six month outcome (predicted probability of good outcome <0.15 *versus* ≥0.15)[41]

6.3 AFTER RANDOMISATION

The completed randomisation form should be filed in the ISF and a copy filed in the participant's medical records. Following randomisation, the TCC will generate and send a letter to inform the participant's GP about their patient's enrolment in the trial, including a copy of the consent form, and the follow-up schedule.

6.4 TREATMENT ALLOCATION

The local researcher will be provided with a participant's treatment allocation on the web or by telephone if the web interface is not operational. The randomisation system will automatically generate an email/fax to the local research team to confirm the treatment allocation (to start or avoid antiplatelet drugs) and if the participant is allocated to start antiplatelet drugs this communication will remind the local researcher about the antiplatelet regimen they specified at the time of randomisation. The local research staff may access treatment allocations through a secure website.

6.5 BLINDING

Treatment allocation in RESTART is not blinded, and therefore it is open to participants, the clinicians caring for them in secondary and primary care, and local research staff. However, the outcome event

adjudication committee and central research staff carrying out telephone follow-up will be blinded to participants' treatment allocation when assessing clinical and radiographic information about outcome events.

7 PREMATURE WITHDRAWAL OF PARTICIPANTS

Participants, or their personal / legal / professional representative, may choose to withdraw from all or part of the trial. If this happens, we will request clarification of which parts of RESTART they are withdrawing from (e,g, participant annual questionnaires, or all follow-up) and we will record the reason for any such withdrawal. We will retain the data collected on the participant up to the point of withdrawal. We will continue to collect outcome data from GPs, registries and other secondary data sources unless the participant withdraws from the trial's methods of passive follow-up.

8 CROSSOVERS

Participants, their personal / legal / professional representative, or their doctor may decide that the participant will stop taking antiplatelet drugs (if allocated to starting them) or start antiplatelet drugs (if allocated to avoiding them). If this happens prior to the occurrence of the primary outcome, the patient will continue to be followed up as per protocol and their data included in the primary analyses. The date of deviation from the allocated treatment and the reason for it will be recorded in the participant's Case Report Form (CRF). If treatment is stopped as a result of a SAE or SUSAR, the event will be reported as per protocol. Such cases are not regarded as premature withdrawals.

9 INVESTIGATIONAL MEDICINAL PRODUCT

9.1 INTERVENTION

RESTART randomises participants to policies of starting or avoiding antiplatelet drugs. The **investigational medicinal products (IMPs) are restricted to the use of one or more of aspirin or dipyridamole** (to which prior ICH is not a contraindication in the Summary of Product Characteristics [SPC]) **or clopidogrel** (to which only 'active pathological bleeding' [judged to occur within the first 24 hours of ICH symptom onset[42]] is a contraindication). The specific antiplatelet drug(s) and their doses will be determined at the discretion of the consultant responsible for the participant, recorded in the CRF, and should be prescribed to start within 24 hours of randomisation. Since participants randomised to starting antiplatelet drug(s) will take licensed antiplatelet drug(s), exemptions from the Good Manufacturing Practices labelling requirements will apply.

9.1.1 COMPARATOR

The comparator is a policy of avoiding antiplatelet drugs. There is no placebo.

9.2 PARTICIPANT ADHERENCE

Regardless of treatment allocation, after randomisation the antiplatelet drug(s) prescribed, and their dose, will be recorded on the hospital/clinic discharge form. Monitoring of adherence for the remainder of the study period will rely on self reporting by the patient or their proxy and on annual questionnaires completed by participants' GPs (accompanied by electronic summaries of prescriptions in primary care).

To increase the likelihood that participants will receive antiplatelet drugs if allocated to a policy of starting them, we will:

- Encourage the randomising clinician to emphasise the importance of adhering to the allocated treatment policy.
- Write to the GP shortly after enrolment to alert them to the participant's inclusion in the trial and ask them to inform us of any changes to the participant's antiplatelet drugs.
- Write to the participant at home (when the hospital discharge form is received) reminding them of the purpose of RESTART and the importance of adhering to their treatment allocation. We will provide them with the means to feedback (by post, telephone, email or web) any concerns, which we would respond to (and include their GPs in our correspondence).

Given the complexities of conducting a trial in this target population where adherence cannot be fully monitored once the patient is discharged from hospital, we fully anticipate that data concerning adherence will be incomplete. In the event that the trial fails to show a difference in outcomes between the policies of starting or avoiding antiplatelet drugs, the data will provide a guide to whether poor adherence might contribute to the lack of effect. Providing we strive to attain those levels of adherence that would be achieved if antiplatelet drugs were known to be effective in this patient group, the results of the trial will be externally valid.

9.3 OVERDOSE

There is a small risk that the participant, or someone close to them, may intentionally or accidentally take an overdose of antiplatelet drugs, but since RESTART is an open trial the ingestion of an overdose of antiplatelet drugs could be recognised and managed conventionally.

9.4 OTHER MEDICATIONS

9.4.1 Non-Investigational Medicinal Products

Aside from assigning policies of starting or avoiding the class of investigational medicinal product (antiplatelet drugs), RESTART does not require the use of non-investigational medicinal products.

9.4.2 Permitted medications

Participants' usual medications are permitted. When we inform each participant's GP of the patient's participation in RESTART, we will make reference to SIGN guidelines recommending antihypertensive drugs for secondary prevention after stroke.[35] RESTART cannot be prescriptive about the use of such treatment, in case the trial contradicts a participant's clinician who has not recommended antihypertensive drugs for other clinical reasons. For findings to be generalisable to clinical practice, we will not aim to optimise blood pressure control as a co-intervention in this trial. However, we do intend to collect data during follow-up on antihypertensive drug prescriptions and blood pressure measurements made by GPs and nurses in the practice electronic patient record.

9.4.3 Prohibited medications

There are no restrictions on medications that participants may take, other than those that are contraindicated because they may interact with the prescribed antiplatelet drug(s) (see SmPC booklet). If the prescription of a contraindicated medication is required in a participant taking antiplatelet drugs (for example, if an outcome event occurs which requires treatment with an anticoagulant), investigators may discontinue antiplatelet drugs until the contraindicated medication is discontinued, when the antiplatelet prescribing strategy allocated in RESTART may be resumed.

9.5 OUTCOME MEASURES

9.5.1 Primary outcome

- **Fatal or non-fatal radiographically- or pathologically-proven recurrent symptomatic ICH.** We define ICH as the abrupt onset of headache, altered level of consciousness, or focal neurological deficit, anatomically referable to a focal collection of blood predominantly located within the brain parenchyma (diagnosed on brain imaging or at autopsy), which was not attributable to prior trauma or haemorrhagic transformation of an ischaemic stroke. This also applies when neurological deterioration occurs with radiographic or pathological evidence of ICH volume growth early after the qualifying ICH (due to either haematoma expansion or re-bleeding).

9.5.2 Secondary outcomes

- **Fatal (i.e. followed by death within 30 days) or non-fatal (i.e. not followed by death within 30 days) serious vascular events:**
    - Symptomatic haemorrhagic events
        - Symptomatic spontaneous or traumatic extradural haemorrhage, subdural haemorrhage, subarachnoid haemorrhage, or intraventricular haemorrhage (not accompanying spontaneous ICH)
        - Symptomatic major extracranial haemorrhage, sub-divided by site (requiring transfusion or endoscopic treatment or surgery, or resulting in death within 30 days)
    - Symptomatic vaso-occlusive events
        - transient ischaemic attack
        - ischaemic stroke
        - acute coronary syndrome
        - peripheral arterial occlusion
        - mesenteric ischaemia
        - retinal arterial occlusion
        - deep vein thrombosis
        - pulmonary embolism
        - Revascularisation procedures (carotid, coronary, or peripheral arterial)
        - Cardiac death with symptoms suggestive of myocardial ischaemia (type 3),[43] or evidence of arrhythmia
    - Symptomatic stroke of uncertain sub-type
        - Non-fatal stroke, with brain imaging performed too late to distinguish ICH from cerebral infarction
        - Rapidly fatal stroke, but without radiographic or pathological confirmation

- **Other fatal events**
    - Deaths without a clear cause and without further investigation
    - Deaths from any other cause

- **Annual ratings of participant function completed by participant or their carer:**
    - Simplified modified Rankin Scale postal questionnaire[44,45]
    - Structured telephone interview with non-responders to the postal questionnaire[46]

10 STUDY ASSESSMENTS AND DATA COLLECTION

10.1 STUDY ASSESSMENT SCHEDULE

The PI, delegated physicians and researcher staff at each site will collect the local data listed in the schedule of study assessments below. The Chief Investigator and the research team in the TCC will collect the central data (see schedule below).

10.1.1 Randomisation

10.1.1.1 Clinical

After establishing eligibility and completing the consent procedures (see 5.2.3), local research staff will complete the randomisation form ready for transcription to the RESTART website at the time of randomisation. Research staff will record the participant's study number and treatment allocation provided on the randomisation form, as well as information about the participant's co-morbidities and qualifying ICH. If a participant is allocated to a policy of starting antiplatelet drugs, research staff will prescribe the antiplatelet regimen they mentioned intending to prescribe on the randomisation form for the standard duration allowed by their hospital pharmacy and communicate the need for ongoing prescription to the GP.

10.1.1.2 Radiographic

Researchers will be required to verify at the time of randomisation that the **brain imaging that diagnosed the qualifying ICH** is available. Brain imaging that diagnosed the ICH will be sent to the TCC in DICOM format. Following receipt, the RESTART imaging manager will check each imaging study to ensure that it relates to the appropriate participant at the appropriate time, that it is the appropriate modality, and that all the required images and sequences have been sent. After quality assurance and obtaining required imaging, these images will be uploaded to an electronic archive and allocated to one of a panel of consultant neuroradiologists via the in-house web-based systematic image review system (www.neuroimage.co.uk/sirsinfo/). The panel member will confirm the diagnosis of ICH, and record imaging data on ICH location (lobar versus deep versus infratentorial), other radiographic evidence of small vessel disease and leukoaraiosis. These data will be stored as part of each participant's electronic CRF in the trial database.

If a participant consents to the **MRI sub-study**, at the time of randomisation the researcher will need to confirm that the MRI has been performed. We require gradient recalled echo (GRE) T2*, T1, T2, FLAIR and DWI sequences to be performed, with susceptibility-weighted imaging if it is available. Imaging obtained for the MRI sub-study will be sent to the TCC in DICOM format and checked by the RESTART imaging manager.

If a participant **has already undergone brain MRI** as part of their routine clinical care with the required sequences prior to randomisation, RESTART will request the MRI and the participant will not need to enter the MRI sub-study.

After quality assurance and obtaining required imaging, these images will be uploaded to an electronic archive and allocated via the in-house web-based systematic image review system to a member of the relevant imaging review panel (Appendix 2) who will record imaging data on ICH location, brain microbleeds using a validated rating scale,[47] other radiographic evidence of small vessel disease, and leukoaraiosis. These data will be stored as part of each participant's electronic CRF.

Researchers will be reimbursed in accordance with the details outlined in the Clinical Study Site Agreement for brain MRI if it is received at the TCC and found to adhere to the MRI protocol by the imaging manager, and the patient is randomised in RESTART.

10.1.2 Discharge

When discharged from the hospital or neurovascular clinic, research staff will complete the RESTART discharge form recording the names of all current medications (and the dosage of antiplatelet drugs prescribed), confirming adherence to the allocated treatment policy, mentioning any outcomes occurring between randomisation and discharge, and providing updated contact details for the participant relating to their discharge destination. When the TCC receives the RESTART discharge form, the participant will be sent a letter from the TCC confirming their treatment allocation.

10.1.3 Long term follow-up

10.1.3.1 Central follow-up

About two weeks before the anniversary of a participant's randomisation, the TCC will send each participant's GP (or hospital consultant, if a discharge form has not been received) a postal questionnaire; if no response is received this will be followed by a telephone reminder four weeks later. In these questionnaires GPs will be asked (and if appropriate paid a fee) to provide details of vital status, the occurrence of primary and secondary outcome events, hospital admissions, recent BP measurements, a list of current medications (specifically enquiring about antiplatelet and antihypertensive drugs and doses), and up-to-date contact details for the participant. We will attempt to use the Clinical Practice Research Datalink (CPRD) and other health informatics developments to extract BP readings from RESTART participants' GP electronic records, but at the very least we will ask GPs, with assistance from the Primary Care Research Networks, to provide BP measurements over the preceding year. If BP measurements have not been taken, we will encourage GPs to monitor BP and remind them to refer to SIGN guidelines.

If the participant is still alive and appropriate for questionnaire follow-up, the participant will be sent a postal questionnaire (if no response is received this will be followed by a telephone reminder four weeks later). Participants will also be given the option of completing the follow-up questionnaire on-line (via a secure web interface) which will provide online help and data validation. Participants will be asked to provide details of the occurrence of primary and secondary outcome events (including modified Rankin Scale score), hospital admissions, recent BP measurements, and a list of medications (specifically enquiring about antiplatelet and antihypertensive drugs and doses). If the participant has incapacity or cannot speak English, their carer will be asked to complete and return the forms. If the follow up information cannot be obtained by either postal or telephone questionnaire the local research team will be asked to arrange a face-to-face follow up. A telephone helpline will be available for participants, carers or GPs to report or discuss outcome events. Experience in previous trials indicates that failure to complete a postal questionnaire usually indicates a failure of receipt or inadvertent non-completion rather than a wish not to participate further in the trial. Central follow-up (telephone or postal) has been found to be cost-effective and efficient.

10.1.3.2 Local follow-up

If a GP or hospital research staff member (5.1.1) reports an outcome event, the TCC will collect the following information, in liaison with the GP and local research staff when required.

- **Non-fatal events:** Medical records of emergency GP assessment, medical and nursing records of A&E department assessment, inpatient medical and nursing records, and brain imaging.
- **Fatal events:** Medical records of emergency GP assessment, medical and nursing records of A&E department assessment, inpatient medical and nursing records, telephone interview with nearest relative (if they agree, and none of the above information can be obtained), brain imaging, death certificate, and Coroner (or Procurator Fiscal) autopsy report. If a participant dies, the clinician can conveniently inform the trial office by completing an on line form or a postal form. Ascertaining the precise date of death will be very important for survival analyses.

10.1.4 Outcome event adjudication

Outcome events that will be adjudicated in RESTART include:

- primary and secondary outcomes (all of which relate to the safety of antiplatelet drugs)
- Serious Adverse Events (SAEs) and Suspected Unexpected Serious Adverse Reactions (SUSARs), which will be reported to the sponsor (see 12.6 and 12.7).

Once all relevant clinical, radiographic or pathological information about an outcome event, SAE, or SUSAR have been obtained, we will obtain a report on any relevant brain imaging from a member of the imaging panel, and then send all the relevant clinical/pathological details with a report of any relevant brain imaging to a member of the clinical event adjudication panel.

10.1.5 Long-term follow-up after the study period

Participants will be followed-up once per year, and long-term follow-up of the trial participants may occur by deterministic linkage with patients' primary care and hospital discharge data and flagging with HSCIC to determine long-term survival. These linkages would be established during the first year after a participant's randomisation, since they may also contribute to the identification of outcomes during follow-up.

	Baseline				Followup
**Assessment**	Pre-randomisation	Randomise	Hospital/clinic discharge	30 days	Annually
Local					
Screen of eligibility	•				
Verify brain imaging that diagnosed qualifying ICH is available	•				
Give PIL to patient and/or carer	•				
Obtain consent	•				
Collect baseline data	•				
Book and obtain MRI prior to randomisation date (if in MRI sub-study	•				
Fax consent form to trial co-ordinating centre	•				
Randomise		•			
Record study number and allocation		•			
Complete enrolment sticker in notes		•			
Prescribe antiplatelet drugs (if allocated to starting) and inform GP		•---			
Send brain imaging that diagnosed qualifying ICH to the trial co-ordinating centre		•			
Send MRI to trial co-ordinating centre (if in MRI sub-study)		•			
Complete discharge form ASAP after death in hospital or discharge			•		
Adverse events			•		
All medications			•		
Adherence			•		
Updated contact details			•		
Clinical information & imaging for outcomes			•		•
Central					
Email/fax notification of treatment allocation to hospital	•				
Write to GP about participation, treatment allocation, & BP control	•				
Prompt to participants about treatment allocation (on receipt of discharge form)			•		
Confirm receipt of brain imaging ± MRI				•	
Confirm receipt of discharge form				•	
GP postal questionnaire ± reminder					•
Outcome events					•
BP measurements					•
All medications					•
Patient postal questionnaire ± reminder					•
Outcome events					•
modified Rankin scale					•
Adherence					•
Newsletters to living participants					•

10.2 SAFETY ASSESSMENTS

The expected adverse reactions to antiplatelet drugs are well known (see 12.2.5), as are the expected non-fatal complications of ICH (see 12.2.6),[48] so safety assessments in RESTART are focussed on detecting: primary and secondary outcomes (all of which relate to the safety of antiplatelet drugs in this patient group) and any additional SAEs and SUSARs that may occur (see section 12).

10.2.1 Types of safety assessment

The PIL given to the participant and/or their carer will mention the known adverse reactions to antiplatelet drugs and the common complications of ICH.

Participants will have a hospital discharge form completed by the researcher at the time of discharge from the hospital or outpatient clinic. The data collected will be entered on a secure web based form or faxed to the TCC to ensure that we are alerted to any important outcome events or adverse reactions. If no discharge form is received 30 days after randomisation, then we will write to the PI to ascertain the occurrence of any outcomes, SAEs or SUSARs.

On receipt of the discharge form, the TCC will write to the participant to remind them about treatment allocation, adherence, and reporting any adverse events. Participants will receive a diary in which they are encouraged to record the date and nature of any adverse events.

All surviving participants will be followed up once per year after randomisation, whether they adhered to their allocated treatment or not. At each follow up the participant and their GP will be asked about adverse events. In order to detect adverse events between the scheduled follow ups a system will be in place to allow the patients, their carers or their GPs to report any adverse reactions to us via:

- Post – freepost envelope and form
- Helpline – telephone phone number which will allow the patients or their doctors to contact the TCC
- Web – secure website where they can contact the TCC

11 STATISTICS AND DATA ANALYSIS

11.1 SAMPLE SIZE CALCULATION

There is considerable uncertainty about the absolute risks of recurrent symptomatic ICH among survivors who were taking antiplatelet drugs at the time their index event, but a review of the literature suggests that the event rate lies in the range of about 1.8 to 7.4% per annum (see section 1.1.2). Similarly, information about the relative increase in the risk of recurrent ICH on antiplatelet drugs is scarce, but estimated relative risks from non-randomised studies have ranged from no excess (RR=1) to a 4-fold excess (RR=4).[27-29] This pilot study of 720 patients will have excellent power (after all participants have been followed for at least two years) to detect a doubling of the rate of ICH if the true rate is 4.5% per annum,[27,29,38,39] but there would be 93% power at the 5% significance level to detect a 4-fold increase in risk of recurrent ICH if the annual risk is only 1%. In both these scenarios the absolute excess risk of recurrent ICH may be higher than any plausible benefit of treatment, in which case it may be inappropriate to consider a larger trial designed to demonstrate net benefit. Previous meta-analyses of RCTs of antiplatelet drugs have provided reliable information on the relative effects of antiplatelet regimens in the sorts of people with vaso-occlusive disease who will have been recruited into RESTART,[38] so information about the overall rates of serious vascular events (for both arms combined) will be used together with reasonable assumptions about the effects of antiplatelet drugs on such events in order to assess the plausibility of a net benefit emerging in a larger main study.

The TSC will review the target sample size and adjust this based on accruing data on: the number of primary outcome events, completeness of follow up, and the enrolment into specific pre-specified subgroups (e.g. lobar ICH location).

Contraindications to MRI, claustrophobia, non-attendances, and scheduling constraints lead to an attrition of ~25% of patients, so we would hope to obtain **brain MRI on ~550 patients** before randomisation. To maximise our chances of recruiting the target number of patients with brain MRI, we will target the set up of sites where brain MRI is available in the first 3-6 months of the start-up phase of RESTART.

11.2 PROPOSED ANALYSES

Our provisional analysis plan is described below, but we intend to publish a final Statistical Analysis Plan before the database is locked for analysis and the results are known. In order to preserve fully the huge benefit of randomisation, we will include all randomised participants in the analysis (irrespective of whether they adhere to the allocated treatment), all retained in the group to which they were allocated (i.e. "as-randomised"). This will comprise a Kaplan Meier survival analysis of time to first outcome event after randomisation. Follow-up will be censored at death (unrelated to an outcome event), last available follow-up, or voluntary withdrawal from the trial. We will compare the survival function in the two trial arms using a Cox proportional hazards regression model, adjusting for all the covariates included in the minimisation algorithm, and presenting the result as an estimated adjusted hazard ratio with its corresponding 95% CI. We will also report the unadjusted estimate of the hazard ratio and its corresponding 95% CI, together with the result of the logrank test.

11.2.1 Primary analysis

This will be restricted to the primary outcome of first fatal or non-fatal radiographically- or pathologically-proven recurrent symptomatic ICH (see 9.5.1).

11.2.2 Secondary analyses

11.2.2.1 Other possible manifestations of recurrent spontaneous ICH

We will conduct two exploratory sensitivity analyses of our primary analysis, by adding the following secondary outcomes (see 9.5.2) to the primary outcome in the following order, to account for the possibility that some fatalities and non-fatal neurological events without adequate investigation may be recurrent ICH:

- Fatal or non-fatal symptomatic stroke consistent with the clinical manifestations of ICH, but without radiographic or pathological confirmation (or with brain imaging performed too late to distinguish ICH from ischaemic stroke).
- Deaths without a clear cause and without further investigation.

11.2.2.2 Other forms of symptomatic spontaneous or traumatic intracranial haemorrhage

- Fatal or non-fatal radiographically- or pathologically-proven spontaneous or traumatic extradural haemorrhage, subdural haemorrhage, subarachnoid haemorrhage, or intraventricular haemorrhage (not accompanying spontaneous ICH).

11.2.2.3 Fatal or non-fatal extracranial haemorrhage

- At any site (but this will mainly be upper or lower gastrointestinal bleeding), requiring transfusion / endoscopic treatment / surgery, or resulting in death.

11.2.2.4 Fatal or non-fatal vaso-occlusive events

- Transient ischaemic attack, ischaemic stroke, unstable angina, myocardial infarction, peripheral arterial occlusion, or retinal arterial occlusion.
- Symptomatic deep vein thrombosis, pulmonary embolism.
- Carotid, coronary, or peripheral arterial revascularisation procedures.

11.2.2.5 Composite outcome of serious vascular events

- Non-fatal myocardial infarction
- Non-fatal stroke
- Death from a vascular cause (including sudden death, pulmonary embolism, haemorrhage, and death from an unknown cause)

11.2.3 Sub-group analyses

We will perform the following sub-group analyses of the primary outcome, and test for sub-group interactions if appropriate:

- Qualifying ICH location (lobar *versus* non-lobar, based on local investigator's interpretation of scan)
- Time since ICH symptom onset (0-6 days, 7-30 days > 30 days)
- Antiplatelet drug(s) that the patient's physician would start if allocated (aspirin alone *versus* other antiplatelet regimen [including combination treatment])
- Participant age at randomisation (<70 years *versus* 70 years or older)
- Predicted six month outcome (predicted probability of good outcome <0.15 *versus* ≥0.15)[41]
- Pre-ICH antithrombotic drug regimen (antiplatelet *versus* anticoagulant)

11.2.4 Brain MRI sub-study

Brain microbleeds on MRI appear to be a biomarker of bleeding-prone microangiopathies (such as arteriolosclerosis and cerebral amyloid angiopathy),[30] they predict recurrent ICH,[30,32] and lobar microbleeds also appeared to modify the effect of aspirin on recurrent ICH risk in one observational study.[27] Furthermore, a collaborative meta-analysis of case-case comparisons has found a preponderance of brain microbleeds amongst patients who had an ICH whilst taking antiplatelet drugs compared to those who were not on any antithrombotics when they had an ICH.[49] So we intend to perform a brain MRI sub-study in ~550 of the 720 participants to better understand these micro-angiopathies by exploring whether the presence, number, or location of brain microbleeds modifies the effect of antiplatelet drugs on the primary outcome.

12 ADVERSE EVENTS

The Principal Investigator is responsible* for the detection and documentation of events meeting the criteria below, which may also be carried out by another suitably qualified physician in the research team at that site, who has up-to-date GCP training and who has been delegated this role (delegated physician).

12.1 DEFINITIONS

12.1.1 Adverse event

An adverse event (AE) is any untoward medical occurrence in a clinical trial participant which does not necessarily have a causal relationship with an IMP.

12.1.2 Adverse reaction

An adverse reaction (AR) is any untoward or unintended response to an IMP which is related to any dose administered to that participant.

12.1.3 Serious adverse event

A **serious adverse event** (SAE) or **serious adverse reaction** (SAR) is any AE or AR that at any dose:

- results in death
- is life threatening* (i.e. the participant was at risk of death at the time of the event; it does not refer to an event which hypothetically might have caused death if it were more severe)
- requires in-patient hospitalisation^ or prolongation of existing hospitalisation
- results in persistent or significant disability or incapacity
- results in a congenital anomaly or birth defect
- results in any other significant medical event not meeting the criteria above (e.g. may jeopardise the participant or may require intervention to prevent one of the other listed criteria).

* Life-threatening in the definition of an SAE or SAR refers to an event where the participant was at risk of death at the time of the event. It does not refer to an event which hypothetically might have caused death if it were more severe.

^ Any hospitalisation that was planned prior to randomisation will not meet SAE criteria. Any hospitalisation that is planned post randomisation will meet the SAE criteria.

12.1.4 Suspected Unexpected Serious Adverse Reaction

A Suspected Unexpected Serious Adverse Reaction (SUSAR) is any AR that is classed as serious and is suspected to be caused by the IMP that is not consistent with the information about the IMP in the SPC.

12.2 ASSESSMENT OF ADVERSE EVENTS

Each AE must be assessed for seriousness, causality, severity and expectedness by the PI or delegated physician.

12.2.1 Assessment of Seriousness

The PI (or delegated physician) will make an assessment of seriousness as defined in Section 12.1.3.

12.2.2 Assessment of Causality

The PI (or delegated physician) will make an assessment of whether the AE/SAE is likely to be related to antiplatelet drugs according to the following definitions:
Unrelated: where an event is not considered to be related to the IMP.

Possibly Related: The nature of the event, the underlying medical condition, concomitant medication or temporal relationship make it possible that the AE has a causal relationship to the study drug.

Alternative causes such as natural history of the underlying disease (see 12.2.6), other risk factors and the temporal relationship of the event to the treatment should be considered and investigated.

Where there are two assessments of an AE, for example, the PI and the CI, the assessment made by the PI cannot be downgraded, but the CI can upgrade an event. In the case of a difference of opinion, both assessments are recorded and the 'worst case' assessment is used for reporting purposes.

12.2.3 Assessment of Severity

The PI (or delegated physician) will make an assessment of severity for each SAE/SUSAR and record this on the CRF or AE form according to one of the following categories:

Mild: an event that is easily tolerated by the participant, causing minimal discomfort and not interfering with every day activities.
Moderate: an event that is sufficiently discomforting to interfere with normal everyday activities.
Severe: an event that prevents normal everyday activities. The term 'severe' should not be confused with 'serious' which is a regulatory definition based on participant/event outcome or action criteria. For example, a headache may be severe but not serious, while a minor stroke is serious but may not be severe.

12.2.4 Assessment of Expectedness

If an event is judged to be an AR/SAR, the evaluation of expectedness will be made based on knowledge of the reaction and the relevant product information (see 12.2.5 and SmPC booklet). The event may be classed as either:

Expected: the AR is consistent with the known toxicity of antiplatelet drugs (see 12.2.5 and SmPC booklet).
Unexpected: the AR is not consistent with the known toxicity of antiplatelet drugs.

12.2.5 Expected adverse reactions to aspirin, dipyridamole and clopidogrel

These lists of known adverse reactions are derived from antiplatelet drugs' SPCs (see SmPC booklet)., and are organised by organ class and known frequency. Note that some ARs are outcomes in RESTART and should be recorded and reported as outcomes.

12.2.6 Expected non-fatal complications of ICH

Patients with ICH experience a variety of events as a result of the natural history of the ICH or expected complications of it, including:

- An ICH-specific complication such as haematoma growth and/or extension into the ventricles, or perihaematomal oedema, with consequent symptomatic deterioration[48]
- Hydrocephalus

- Epileptic seizure(s)
- Fever
- Hyperglycaemia
- Raised blood pressure
- Chest infections
- Urinary tract infections
- Other infections including those of soft tissues
- Renal dysfunction
- Painful shoulder syndromes
- Pressure sores
- Spasticity or contractures
- Depression, emotionalism
- Any other expected complications of stroke

12.3 DETECTING ADVERSE EVENTS

All outcomes and AEs will be sought from the time a participant signs the consent form to take part in the study until death or the completion of trial follow-up, using follow-up methods (10.1.3) and safety assessments (10.2) as described above.

12.4 REPORTING ADVERSE EVENTS

If a PI or delegated physician becomes aware of an adverse event, it is their responsibility to review all documentation (e.g. hospital notes, laboratory and diagnostic reports) related to the event, and then decide whether it is an outcome in the trial (see 9.5), an expected non-fatal complication of ICH (see 12.2.6), a known adverse reaction to antiplatelet drugs (see 12.2.5 and SmPC booklet), a SAE, or a SUSAR (see flowchart overleaf).

Information to be collected includes dose, type of event, onset date, PI assessment of severity and causality, date of resolution as well as treatment required, investigations needed and outcome. If the adverse event is detected by central means of follow-up, the TCC will initiate the collection of this information, but enlist the help of local personnel in the NIHR Clinical Research Network (CRN) to acquire the relevant clinical and imaging information.

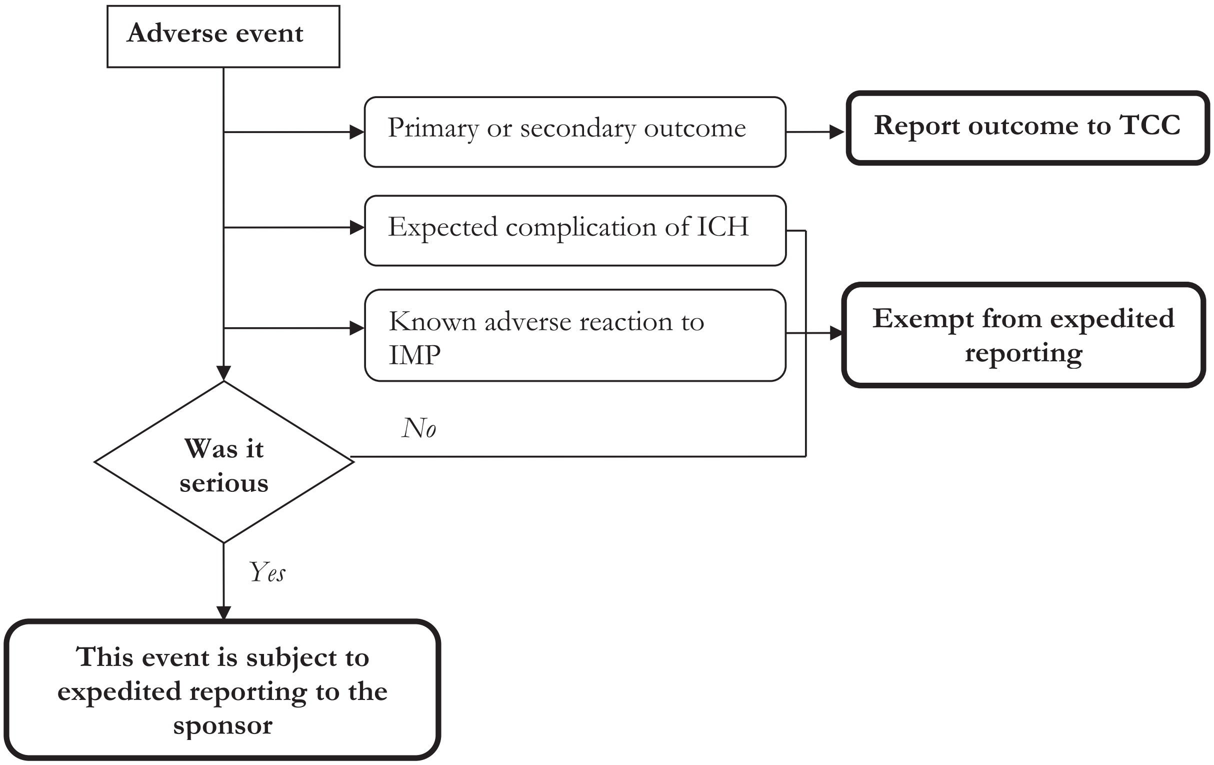

12.5 ADVERSE EVENT REPORTING IN RESTART

The expected adverse reactions to the antiplatelet drugs that may be used in RESTART (aspirin, clopidogrel, or dipyridamole) are well known (see 12.2.5), as are the expected non-fatal complications of ICH (see 12.2.6),[48] so safety assessments in RESTART are focussed on detecting: primary and secondary outcomes (all of which relate to the safety of antiplatelet drugs in this patient group) and any SAEs and SUSARs that may occur. These data will be presented to the DMC and reported in the Development Safety Update Report. Our central follow-up mechanisms will also systematically collect information on hospital admissions and new medications, using multiple overlapping sources of ascertainment, which will provide an additional alerting system.

12.5.1 Investigators <u>should</u> report outcomes to the Trial Co-ordinating Centre

Primary (9.5.1) and secondary (9.5.2) outcomes must be reported on the outcome event form to the TCC, at the time of hospital/clinic discharge, or during long-term follow-up if a member of the local research team becomes aware of an event during follow-up.

12.5.2 Investigators <u>must</u> report SAEs and SUSARs to the Trial sponsor

The following must be reported to the ACCORD office within 24 hours of the CI or PI becoming aware of the event (see 12.6 below):

- SUSARs
- SAEs which are neither outcomes (see 9.5), nor known adverse reactions to antiplatelet drugs (see 12.2.5 and SPC booklet), nor expected non-fatal complications of ICH (see 12.2.6).

12.5.3 Investigators should not report other events to the Trial Co-ordinating Centre or sponsor

PIs need not report to the TCC or sponsor any non-fatal AEs that are neither primary/secondary trial outcomes nor SAEs nor SUSARs, and which are expected complications of ICH (see 12.2.6).[48]

12.6 REPORTING OF SAES AND SUSARS

The Investigator is responsible for reporting SAEs to ACCORD within 24 hours of becoming aware of the event.

Initial reporting of reportable adverse events should be done using the electronic form on the RESTART trial website. If the event is serious you should report it to ACCORD within 24 hours using the sponsor **CR0005** form.

SAE, SAR and SUSAR reports will either be emailed as a .pdf file to Safety@ACCORD.scot; delivered in person to a member of the Pharmacovigilance team or faxed to ACCORD on +44 (0)131 242 9447 using Template report CR005-T01 (SAE Report Form (CTIMP)) or template report CR005-T04 (Parent-Child (SAE) Form (CTIMP)) and the Cover Sheet and Return Receipt (CR005-F01). Reports will be complete as far as possible and will be signed and dated by the Investigator.

The Research Governance Coordinator, or designee, will complete and return the Cover Sheet and Return Receipt (CR005-F01) or send an email to confirm receipt of the SAE, SAR or SUSAR report within 1 working day. If this email/fax is not received within 1 working day of sending the report to ACCORD, the Investigator must telephone ACCORD on +44 (0)131 242 3330to check that the report has been received by ACCORD.

Once an SAE, SAR or SUSAR report is received by ACCORD it will be entered onto the ACCORD PhV database by the Research Governance Coordinator, or designee.

All SAE, SAR and SUSAR reports emailed or faxed to ACCORD and any follow-up information and correspondence will be kept by the Investigator in the Investigator Site File (ISF) and by the Sponsor in the Trial Master File (TMF).

Follow-up
After recording and reporting safety events, it is the responsibility of the Investigator to follow-up the affected participant(s) until resolution of the event or death of the participant(s).

If the outcome of an initial report of an event is one of the following outcome options:

- Condition still present and unchanged
- Condition deteriorated
- Condition improving

Then the Investigator must follow-up with the participant(s). Unless otherwise defined in the protocol, a safety report will not be considered complete until the outcome is:

- Completely recovered (including date of recovery)
- Recovered with sequelae (including date of recovery)
- Death (including date of death).

12.7 REGULATORY REPORTING REQUIREMENTS

The ACCORD Research Governance & QA Office is responsible for pharmacovigilance reporting on behalf of the co-sponsors (Edinburgh University and NHS Lothian). The ACCORD Research Governance & QA Office has a legal responsibility to notify the regulatory competent authority and relevant ethics committee (Research Ethics Committee (REC) that approved the trial). Fatal or life threatening SUSARs will be reported no later than 7 calendar days and all other SUSARs will be reported no later than 15 calendar days after ACCORD is first aware of the reaction. ACCORD will inform PI at participating sites of all SUSARs and any other arising safety information. A Development Safety Update Report will be submitted to the regulatory competent authority and main REC annually listing all SAEs and SUSARs.

13 PREGNANCY

Pregnancy is not considered an adverse event; however, the PI will collect pregnancy information for any female participants or female partners of male participants who become pregnant while participating in the study. The PI will record the information on a Pregnancy Notification Form and submit this to the ACCORD office within 14 days of being made aware of the pregnancy. All pregnant female participants and partners of male participants will be followed up until following the outcome of the pregnancy.

14 TRIAL MANAGEMENT AND OVERSIGHT ARRANGEMENTS

14.1 ROLE OF FUNDER AND SPONSOR

The study funder (British Heart Foundation) and co-sponsors (University of Edinburgh and NHS Lothian) did not influence trial design. The sponsor ensures that data collection, management and study monitoring are conducted appropriately (see below). Neither the funder nor the sponsor will have ultimate authority over writing of the report or the decision to submit the report for publication.

14.2 TRIAL MANAGEMENT GROUP

The trial will be coordinated by a Trial Management Group (Appendix 2), consisting of the Chief Investigator, Chief Imaging Investigator, the Trial Manager and Imaging Manager, Trial Statistician and other project staff. The Trial Manager will oversee the study and will be accountable to the Chief Investigator. This group will be based in the University of Edinburgh Neurosciences Trials Unit, affiliated to our UKCRC-registered Edinburgh Clinical Trials Unit (ECTU; www.clinicaltrials.ed.ac.uk). This group will meet at least monthly.

14.3 TRIAL CO-ORDINATING CENTRE

The TCC is responsible for all aspects of the management of RESTART and is based at the Neurosciences Trials Unit, Edinburgh Clinical Trials Unit, at the University of Edinburgh. Responsibilities include: regulatory submissions and compliance; financial management; monitoring of sites; training; patient information and communication; outcome assessment; data collection systems and data management; statistical analysis; reports and publications and archiving of the TMF in accordance with funder and sponsor requirements. Documentation of these study procedures is kept at the TCC.

14.4 TRIAL STEERING COMMITTEE

A TSC oversees the conduct and progress of the trial (Appendix 2). A statement of their competing interests is available on request.

14.5 DATA MONITORING COMMITTEE

A DMC (Appendix 2), which is independent of the sponsor, oversees the safety of participants in the trial, according to the terms of reference in the DMC Charter, which is available from the TCC.[50] During the period of recruitment into the study, interim analyses of the baseline and follow up data will be supplied, in strict confidence, to the chairman of the DMC, along with any other analyses that the committee may request. In the light of these analyses, the DMC will advise the chairman of the TSC if, in their view, the randomised comparisons have provided both (i) 'proof beyond reasonable doubt' that for all, or some, the treatment is clearly indicated or clearly contra-indicated and (ii) evidence that might reasonably be expected to materially influence future patient management. Appropriate criteria of proof beyond reasonable doubt cannot be specified precisely, but the DMC will work on the principle that a difference of at least 3 standard errors in an interim analysis of a major outcome event (e.g. the primary outcome) may be needed to justify halting, or modifying, a study before the planned completed recruitment. This criterion has the practical advantage that the exact number of interim analyses would be of little importance, and so no fixed schedule is proposed. Following a report from the DMC, the TSC will decide whether to modify entry to the study (or seek extra data). Unless this happens however, the TSC, the collaborators and central administrative staff will remain ignorant of the interim results.

14.6 INSPECTION OF RECORDS

Research staff and institutions involved in the study will permit trial related monitoring and audits on behalf of the sponsor, REC review, and regulatory inspection(s). In the event of an audit or monitoring, the PI agrees to allow the representatives of the sponsor direct access to all study records and source documentation. In the event of regulatory inspection, the PI agrees to allow inspectors direct access to all study records and source documentation.

14.7 RISK ASSESSMENT

An independent risk assessment will be performed by an ACCORD Clinical Trials Monitor to determine if monitoring is required and if so, at what level, and a risk-adapted monitoring plan. An independent risk assessment will also be carried out by the ACCORD Quality Assurance Group to determine if an audit should be performed before/during/after the study and if so, at what locations and at what frequency.

14.8 STUDY MONITORING AND AUDIT

RESTART is a large, pragmatic, trial randomising participants between *policies* of starting or avoiding antiplatelet drugs (which have well established safety profiles). The trial will routinely collect data on outcomes, SAEs, and SUSARs, and these will be reviewed by the independent DMC. The trial procedures are based on routine clinical procedures and include (1) prescription of antiplatelet drugs in routine clinical practice for standard indications; (2) collecting routine clinical information from the medical records; and (3) informed consent. There are no complex procedures or interventions for the participants or research staff in this trial. Clinical management for underlying conditions will remain as per each hospital's standard protocol. Based on these factors, the probability of harm or injury (physical, psychological, social or economic) occurring as a result of participation in this research study is considered to be low in each of these categories. The RESTART internal monitoring procedure to

assure appropriate conduct of the trial will use a combination of central data monitoring and remote self monitoring unless issues are identified that can only be addressed by site monitoring in accordance with the Monitoring Plan agreed by the sponsor. This will be regularly reviewed during the course of the trial.

14.8.1 Archiving of site data

All trial related and source documents should be archived for five years in accordance with the Sponsor's archiving policy unless an alternative longer archiving period is specified by the sponsor or the funder The costs for this must be discussed and agreed locally by each research and development (R&D) department as part of the R&D approval process.

14.8.2 Archiving of central data

All trial related documents will be archived for five years in accordance with the Sponsor's archiving policy unless an alternative longer archiving period is specified by the sponsor or the funder. The TSC will have access to the final trial data set, and will consider applications to access the dataset by investigators or others.

15 GOOD CLINICAL PRACTICE

15.1 ETHICAL CONDUCT

The study will be conducted in accordance with the principles of GCP. A favourable ethical opinion will be obtained from the appropriate REC and local R&D approval will be obtained prior to starting the study.

15.2 REGULATORY COMPLIANCE

The study will not start until a Clinical Trial Authorisation (CTA) is obtained from the appropriate Regulatory Authority. The protocol and study conduct will comply with the Medicines for Human Use (Clinical Trials) Regulations 2004, and any relevant amendments.

15.3 PRINCIPAL INVESTIGATOR RESPONSIBILITIES

The PI is responsible for the overall conduct of the study at the site and ensuring any person delegated responsibilities is fully informed, understands and is fully compliant with the protocol and any protocol amendments. In accordance with the principles of GCP, the following areas listed in this section are also the responsibility of the PI. Responsibilities may be delegated to an appropriately trained member of study site staff. Responsibilities must not be delegated or duties undertaken until a CV, proof of current GCP certification and any other relevant training certificates have been collected and reviewed by the PI and details of the person and their responsibilities clearly documented on the Delegation Log and signed by the PI and those people delegated responsibilities.

15.3.1 Confirming participant eligibility and informed consent

Although a research nurse may be delegated the responsibility for identifying suitable patients, obtaining consent (see section 5.2) and randomising the patient, the PI or physician sub investigator

must confirm in writing in the medical records that the patient fulfils the eligibility criteria and must prescribe antiplatelet drugs (if the participant is randomised to a policy of starting them). The PI is responsible for ensuring informed consent is obtained before any protocol specific procedures are carried out (detailed in section 5.2).

15.3.2 Study Site Staff

The research staff must be familiar with antiplatelet drugs, the protocol and the study requirements. It is the PI's responsibility to ensure that all staff assisting with the study are adequately informed about antiplatelet drugs, the protocol and their trial related duties.

15.3.3 Data Recording

The PI is responsible for the quality of the data recorded in the CRF at each Investigator Site.

15.3.4 Investigator Documentation

Prior to beginning the study, each PI will be asked to provide particular essential documents to the TCC (on behalf of the sponsor), including but not limited to:

- An original signed PI's Declaration (as part of the Clinical Study Site Agreement);
- Curriculum vitae (CV) signed and dated by the PI indicating that it is accurate and current.

The TCC will ensure all other documents required by GCP are retained in a TMF and that appropriate documentation is available in local ISFs.

15.3.5 GCP Training

All study staff must hold evidence of appropriate GCP training or undergo GCP training. This should be updated every two years throughout the trial or in accordance with local R&D procedures if more frequent.

15.3.6 Confidentiality

All evaluation forms, reports, and other records must be identified in a manner designed to maintain participant confidentiality. All records must be kept in a secure storage area with limited access. Clinical information will not be released without the written permission of the participant. The PI and study site staff involved with this study may not disclose or use for any purpose other than performance of the study, any data, record, or other unpublished, confidential information disclosed to those individuals for the purpose of the study. Prior written agreement from the sponsor or its designee must be obtained for the disclosure of any said confidential information to other parties.

15.3.7 Data Protection

All PIs and study site staff involved with this study must comply with the requirements of the Data Protection Act 1998 with regard to the collection, storage, processing and disclosure of personal information and will uphold the Act's core principles. Access to collated participant data will be restricted to those clinicians treating the participants. Computers used to collate the data will have limited access measures via user names and passwords. Published results will not contain any personal data that could allow identification of individual participants.

15.3.8 Follow-up

The PI is responsible for follow up of participants recruited as inpatients until hospital discharge or death (whichever occurs first) or, for participants recruited as outpatients, until the patient has been prescribed antiplatelet drug(s) (if allocated to starting them). In exceptional circumstances, where central follow-up has failed, the TCC may ask the PI to collect annual follow up data. If an outcome event occurs, the PI and NIHR CRN staff will be responsible for assisting the TCC staff in obtaining clinical, imaging or pathological information required for outcome event adjudication.

16 STUDY CONDUCT RESPONSIBILITIES

16.1 PROTOCOL AMENDMENTS

Any changes in research activity, except those necessary to remove an apparent, immediate hazard to the participant in the case of an urgent safety measure, must be reviewed and approved by the CI. Amendments to the protocol must be submitted in writing to the appropriate REC, Regulatory Authority and local R&D for approval prior to participants being enrolled into an amended protocol. The TCC will be responsible for disseminating information about protocol amendments to investigators, ethics committees, other regulators, trial participants, and trial registries.

16.2 PROTOCOL VIOLATIONS AND DEVIATIONS

The Chief Investigator (CI)/Principal Investigator (PI) should not implement any deviation from, or changes to the protocol without prior review of an amendment, and documented approval/favourable opinion from the Sponsor/Research Ethics Committee and the Competent Authority and R&D (if applicable), except where necessary to eliminate an immediate hazard(s) to trial subjects.

A departure from the approved clinical trial protocol or from Good Clinical Practice (GCP) must be identified and recorded as a deviation or a violation.

The PI, and clinical team on site, are responsible for identifying deviations or violations as they occur.

Responsibility for completing the forms and maintaining the log may be delegated to suitably qualified named persons on the Study Delegation Log.

The PI is responsible for review of deviations and violations at their site.

Reporting: Deviations
Initial reporting of deviations should be done using the electronic form on the RESTART trial website. Enter the following details, Patient ID, Event Date, Description of the deviation, Corrective actions (what was done to try to rectify this), Preventative actions (what was done to prevent a reoccurrence), Impact statements (effects on safety and/or study outcomes)*, Your contact details

Print a copy of the deviation report and file in the Investigator Site file

> * By answering yes to either of the impact statements you are identifying the event as a protocol violation which must be reported to the ACCORD office within 3 days of becoming aware of the violation.

Reporting: Violations

The latest version of the ACCORD violation form go to CRO10 at http://www.accord.ed.ac.uk/research-access/resources-researchers/sop

Violation forms will be transmitted via email to QA@accord.scot Only forms in a pdf format will be accepted by ACCORD via email. Forms may also be sent by fax to ACCORD on **+44 (0)131 242 9447** or may be submitted by hand to the office. Where missing information has not been sent to ACCORD after an initial report, ACCORD will contact the Investigator and request the missing information. The Investigator must respond to these requests in a timely manner.

A quarterly report of all deviations reported to the RESTART trials office will be emailed to the sponsor. On receipt of the deviation logs, the sponsor's Senior Clinical Trials Monitor, or designee, will assess the deviations to ensure the correct assessment has been made. Once confirmed as complete, the deviation logs will be filed in the Trial Master File (TMF).

16.3 SERIOUS BREACH REQUIREMENTS

A serious breach is a breach which is likely to effect to a significant degree:
(a) the safety or physical or mental integrity of the participants of the trial; or
b) the scientific value of the trial.

If a potential serious breach is identified by the Chief investigator, PI or delegates, the co-sponsors (accord.seriousbreach@ed.ac.uk) must be notified within 24 hours. It is the responsibility of the co-sponsors to assess the impact of the breach on the scientific value of the trial, to determine whether the incident constitutes a serious breach and take the appropriate action. Not every violation from the protocol needs to be reported to the regulatory authority as a serious breach. If the sponsor(s) deem the incident to be a violation that does not constitute a serious breach from the protocol when identified, corrective and preventative actions will be taken where appropriate and they will be recorded in file notes, held within the TMF and ISF.

16.4 END OF STUDY

The end of study is defined as the last follow-up received. The CI and/or the TSC have the right at any time to terminate the study for clinical or administrative reasons.

The end of the study will be reported to the REC and Regulatory Authority within 90 days, or 15 days if the study is terminated prematurely. The Investigaotors will inform participants of the premature study closure and ensure that the appropriate follow up is arranged for all participants involved. End of study notification will be reported to the co-sponsors via email to resgov@ed.ac.uk.

In accordance with ACCORD SOP CR011, a Clinical Study Report (CSR) will be provided to the Sponsor (QA@accord.scot) and REC within 1 year of the end of the study.

Upon completion of the study, the Investigator will upload clinical trial results onto the EudraCT database on behalf of the Sponsor.

The Investigator will submit a short confirmatory e-mail to the MHRA (CT.Submission@mhra.gsi.gov.uk) once the result-related information has been uploaded to EudraCT, with 'End of trial: result-related information: EudraCT 2012-003190-26 as the subject line. The Sponsor(s) will be copied in this e-mail (QA@accord.scot).

16.5 CONTINUATION OF DRUG FOLLOWING THE END OF STUDY

Antiplatelet drugs may be continued indefinitely after the end of the trial period. There are no provisions for ancillary or post-trial care for participants.

16.6 INSURANCE AND INDEMNITY

The co-sponsors are responsible for ensuring proper provision has been made for insurance or indemnity to cover their liability and the liability of the CI and staff.

The following arrangements are in place to fulfil the co-sponsors' responsibilities:

- The Protocol has been designed by the CI and researchers employed by the University and collaborators. The University has insurance in place (which includes no-fault compensation) for negligent harm caused by poor protocol design by the CI and researchers employed by the University.
- Sites participating in the study will be liable for clinical negligence and other negligent harm to individuals taking part in the study and covered by the duty of care owed to them by the sites concerned. The co-sponsors require individual sites participating in the study to arrange for their own insurance or indemnity in respect of these liabilities.
- Sites which are part of the United Kingdom's Nation Health Service will have the benefit of NHS Indemnity.
- Sites out with the United Kingdom will be responsible for arranging their own indemnity or insurance for their participation in the study, as well as for compliance with local law applicable to their participation in the study.

17 REPORTING, PUBLICATIONS AND NOTIFICATION OF RESULTS

17.1 AUTHORSHIP POLICY

Ownership of the data arising from this study resides with the TSC. On completion of the trial, the data will be analysed and tabulated, and a clinical study report will be prepared in accordance with GCP guidelines. The success of this trial depends entirely on the collaboration of a large number of doctors, nurses, other health professionals, patients and relatives. Those included in the Delegation Logs will be included in any listing of collaborators. For this reason the credit for the main results will be given, not exclusively to the central trial coordinators, but to all wholehearted collaborators in the study. The primary trial publication will be drafted by a writing committee whose membership has been approved by the TSC. The manuscript must be approved by the TSC before submission for publication.

17.2 PUBLICATION

The clinical study report will be used for publication and presentation at scientific meetings. The CI has the right to publish orally or in writing the results of the study. The results will also be disseminated to participants and the public via the trial website and social media. Summaries of results will also be made available to PIs for dissemination within their clinics (where appropriate and according to their discretion).

APPENDIX 1: COMMITTEES

TRIAL STEERING COMMITTEE

Prof Colin N. Baigent (independent chairperson)
Professor of epidemiology, University of Oxford
Prof Rustam Al-Shahi Salman (chief investigator)
MRC senior clinical fellow, University of Edinburgh & honorary consultant neurologist, NHS Lothian
Dr Shannon Amoils (funding representative)
Senior Research Adviser, British Heart Foundation
Bernadette Gallagher (co-sponsor representative)
ACCORD, University of Edinburgh
Prof Martin S. Dennis
Professor of stroke medicine, University of Edinburgh
Prof Gordon D. Murray
Professor of medical statistics, University of Edinburgh
Prof David E. Newby
BHF Professor of cardiology, University of Edinburgh
Prof Peter A.G. Sandercock
Professor of medical neurology, University of Edinburgh
Prof Cathie L.M. Sudlow
Professor of neurology and clinical epidemiology, University of Edinburgh
Dr Daniel Lasserson
Senior Clinical Researcher, Nuffield department of primary care health sciences, University of Oxford
Prof Phil M. White (chief imaging investigator)
Professor of interventional and diagnostic neuroradiology, University of Newcastle
Dr William N. Whiteley
MRC clinician scientist, University of Edinburgh & honorary consultant neurologist, NHS Lothian
Dr David Werring
Reader in clinical neurology, University College London
Ms Karen Innes (trial manager)
FOCUS and RESTART trial manager, University of Edinburgh

TRIAL MANAGEMENT GROUP

Prof Rustam Al-Shahi Salman (chief investigator)
MRC senior clinical fellow, University of Edinburgh & honorary consultant neurologist, NHS Lothian
Ms Karen Innes (trial manager)
FOCUS and RESTART trial manager, University of Edinburgh
Ms Lynn Forrester (imaging manager)
Imaging manager, University of Edinburgh
Ms Carol Williams (trial site coordinator)
Neurosciences Trials Unit, ECTU, University of Edinburgh
Mr Jonathan Drever (data manager)
Neurosciences Trials Unit, ECTU, University of Edinburgh
Mr Connor McGill (programmer)
Neurosciences Trials Unit, ECTU, University of Edinburgh
Ms Ruth Fraser (trial administrator)
Neurosciences Trials Unit, ECTU, University of Edinburgh
Ms Pauli Walker (trial administrator)
Neurosciences Trials Unit, ECTU, University of Edinburgh

DATA MONITORING COMMITTEE

Dr John Bamford (chairperson)
Consultant neurologist and Clinical Associate Professor, Leeds General Infirmary, Leeds
Prof Jane Armitage
Professor of Clinical Trials and Epidemiology, CTSU, University of Oxford
Dr Jonathan Emberson
Senior statistician, CTSU, University of Oxford
Professor Gabriel Rinkel
Professor of neurology, University Medical Center, Utrecht, The Netherlands
Professor Gordon Lowe
Honorary senior research fellow, Institute of Cardiovascular and Medical Sciences, University of Glasgow

IMAGING REVIEW PANELS

Computed tomography
Dr Amrish Mehta (Charing Cross Hospital, London)
Dr Yogish Yoshi (University Hospital of Wales, Cardiff)
Dr Johann Du Plessis (Southern General Hospital, Glasgow)

Magnetic resonance imaging
Prof Phil White (Royal Victoria Infirmary, Newcastle)
Dr Dipayan Mitra (Royal Victoria Infirmary, Newcastle)
Dr Priya Bhatnagar (Royal Victoria Infirmary, Newcastle)
Dr Elena Adela Cora (Royal Victoria Infirmary, Newcastle)

APPENDIX 2: REFERENCES

(1) Al-Shahi Salman R, Labovitz DL, Stapf C. Spontaneous intracerebral haemorrhage. BMJ 2009; 339:b2586.

(2) van Asch CJ, Luitse MJ, Rinkel GJ, van der Tweel I, Algra A, Klijn CJ. Incidence, case fatality, and functional outcome of intracerebral haemorrhage over time, according to age, sex, and ethnic origin: a systematic review and meta-analysis. Lancet Neurol 2010; 9(2):167-176.

(3) Andersen KK, Olsen TS, Dehlendorff C, Kammersgaard LP. Hemorrhagic and ischemic strokes compared: stroke severity, mortality, and risk factors. Stroke 2009; 40(6):2068-2072.

(4) Viswanathan A, Rakich SM, Engel C, Snider R, Rosand J, Greenberg SM et al. Antiplatelet use after intracerebral hemorrhage. Neurology 2006; 66(2):206-209.

(5) Passero S, Burgalassi L, D'Andrea P, Battistini N. Recurrence of bleeding in patients with primary intracerebral hemorrhage. Stroke 1995; 26(7):1189-1192.

(6) O'Donnell HC, Rosand J, Knudsen KA, Furie KL, Segal AZ, Chiu RI et al. Apolipoprotein E genotype and the risk of recurrent lobar intracerebral hemorrhage. New England Journal of Medicine 2000; 342(4):240-245.

(7) Bailey RD, Hart RG, Benavente O, Pearce LA. Recurrent brain hemorrhage is more frequent than ischemic stroke after intracranial hemorrhage. Neurology 2001; 56(6):773-777.

(8) Feng W, Hendry RM, Adams RJ. Risk of recurrent stroke, myocardial infarction, or death in hospitalized stroke patients. Neurology 2010; 74(7):588-593.

(9) Hanger HC, Wilkinson TJ, Fayez-Iskander N, Sainsbury R. The risk of recurrent stroke after intracerebral haemorrhage. J Neurol Neurosurg Psychiatry 2007; 78(8):836-840.

(10) McGuire AJ, Raikou M, Whittle I, Christensen MC. Long-term mortality, morbidity and hospital care following intracerebral hemorrhage: an 11-year cohort study. Cerebrovasc Dis 2007; 23(2-3):221-228.

(11) Vermeer SE, Algra A, Franke CL, Koudstaal PJ, Rinkel GJ. Long-term prognosis after recovery from primary intracerebral hemorrhage. Neurology 2002; 59(2):205-209.

(12) Arakawa S, Saku Y, Ibayashi S, Nagao T, Fujishima M. Blood pressure control and recurrence of hypertensive brain hemorrhage. Stroke 1998; 29(9):1806-1809.

(13) Bae H, Jeong D, Doh J, Lee K, Yun I, Byun B. Recurrence of bleeding in patients with hypertensive intracerebral hemorrhage. Cerebrovascular Diseases 1999; 9(2):102-108.

(14) Hill MD, Silver FL, Austin PC, Tu JV. Rate of stroke recurrence in patients with primary intracerebral hemorrhage. Stroke 2000; 31(1):123-127.

(15) Saloheimo P, Lapp TM, Juvela S, Hillbom M. The impact of functional status at three months on long-term survival after spontaneous intracerebral hemorrhage. Stroke 2006; 37(2):487-491.

(16) Inagawa T. Recurrent primary intracerebral hemorrhage in Izumo City, Japan. Surgical Neurology 2005; 64(1):28-35.

(17) Zia E, Engstrom G, Svensson PJ, Norrving B, Pessah-Rasmussen H. Three-year survival and stroke recurrence rates in patients with primary intracerebral hemorrhage. Stroke 2009; 40(11):3567-3573.

(18) Cheung CM, Tsoi TH, Hon SF, Au-Yeung M, Shiu KL, Lee CN et al. Outcomes after first-ever stroke. Hong Kong Medical Journal 2007; 13(2):95-99.

(19) Azarpazhooh MR, Nicol MB, Donnan GA, Dewey HM, Sturm JW, Macdonell RA et al. Patterns of stroke recurrence according to subtype of first stroke event: the North East Melbourne Stroke Incidence Study (NEMESIS). International Journal of Stroke 2008; 3(3):158-164.

(20) Tanahashi N, Nakagawara J, Okada Y, Minematsu K, Challenge-Stroke Study Group. Candesartan cilexetil in the management of blood pressure for acute and recurrent stroke in Japan: the Challenge-Stroke study. Expert Review of Cardiovascular Therapy 2011; 9(9):1115-1126.

(21) Weimar C, Benemann J, Terborg C, Walter U, Weber R, Diener HC et al. Recurrent stroke after lobar and deep intracerebral hemorrhage: a hospital-based cohort study. Cerebrovascular Diseases 2011; 32(3):283-288.

(22) Yokota C, Minematsu K, Hasegawa Y, Yamaguchi T. Long-term prognosis, by stroke subtypes, after a first-ever stroke: a hospital-based study over a 20-year period. Cerebrovascular Diseases 2004; 18(2):111-116.

(23) Baigent C, Blackwell L, Collins R, Emberson J, Godwin J, Peto R et al. Aspirin in the primary and secondary prevention of vascular disease: collaborative meta-analysis of individual participant data from randomised trials. Lancet 2009; 373(9678):1849-1860.

(24) Aguilar M, Hart R. Antiplatelet therapy for preventing stroke in patients with non-valvular atrial fibrillation and no previous history of stroke or transient ischemic attacks. Cochrane Database Syst Rev 2005;(4):CD001925.

(25) Flynn RW, MacDonald TM, Murray GD, Doney AS. Systematic review of observational research studying the long-term use of antithrombotic medicines following intracerebral hemorrhage. Cardiovasc Ther 2010; 28(3):177-184.

(26) Wani M, Nga E, Navaratnasingham R. Should a patient with primary intracerebral haemorrhage receive antiplatelet or anticoagulant therapy? BMJ 2005; 331(7514):439-442.

(27) Biffi A, Halpin A, Towfighi A, Gilson A, Busl K, Rost N et al. Aspirin and recurrent intracerebral hemorrhage in cerebral amyloid angiopathy. Neurology 2010; 75(8):693-698.

(28) Chong BH, Chan KH, Pong V, Lau KK, Chan YH, Zuo ML et al. Use of aspirin in Chinese after recovery from primary intracranial haemorrhage. Thromb Haemost 2012; 107(2):241-247.

(29) Flynn RW, MacDonald TM, Murray GD, MacWalter RS, Doney AS. Prescribing antiplatelet medicine and subsequent events after intracerebral hemorrhage. Stroke 2010; 41(11):2606-2611.

(30) Cordonnier C, Al-Shahi Salman R, Wardlaw J. Spontaneous brain microbleeds: systematic review, subgroup analyses and standards for study design and reporting. Brain 2007; 130(Pt 8):1988-2003.

(31) Greenberg SM, Vernooij MW, Cordonnier C, Viswanathan A, Al-Shahi SR, Warach S et al. Cerebral microbleeds: a guide to detection and interpretation. Lancet Neurol 2009; 8(2):165-174.

(32) Greenberg SM, Eng JA, Ning M, Smith EE, Rosand J. Hemorrhage burden predicts recurrent intracerebral hemorrhage after lobar hemorrhage. Stroke 2004; 35(6):1415-1420.

(33) Morgenstern LB, Hemphill JC, III, Anderson C, Becker K, Broderick JP, Connolly ES, Jr. et al. Guidelines for the management of spontaneous intracerebral hemorrhage: a guideline for healthcare professionals from the American Heart Association/American Stroke Association. Stroke 2010; 41(9):2108-2129.

(34) Steiner T, Kaste M, Forsting M, Mendelow D, Kwiecinski H, Szikora I et al. Recommendations for the management of intracranial haemorrhage - part I: spontaneous intracerebral haemorrhage. The European Stroke Initiative Writing Committee and the Writing Committee for the EUSI Executive Committee. Cerebrovasc Dis 2006; 22(4):294-316.

(35) Scottish Intercollegiate Guidelines Network. Management of patients with stroke or TIA: assessment, investigation, immediate management and secondary prevention. Guideline No.108 . 2008.
Ref Type: Generic

(36) Glasziou P, Chalmers I, Rawlins M, McCulloch P. When are randomised trials unnecessary? Picking signal from noise. BMJ 2007; 334(7589):349-351.

(37) Sung JJ, Lau JY, Ching JY, Wu JC, Lee YT, Chiu PW et al. Continuation of low-dose aspirin therapy in peptic ulcer bleeding: a randomized trial. Ann Intern Med 2010; 152(1):1-9.

(38) Antithrombotic Trialists' Collaboration. Collaborative meta-analysis of randomised trials of antiplatelet therapy for prevention of death, myocardial infarction, and stroke in high risk patients. BMJ 2002; 324(7329):71-86.

(39) McQuaid KR, Laine L. Systematic review and meta-analysis of adverse events of low-dose aspirin and clopidogrel in randomized controlled trials. Am J Med 2006; 119(8):624-638.

(40) Cordonnier C, Klijn CJ, van Beijnum J, Al-Shahi Salman R. Radiological investigation of spontaneous intracerebral hemorrhage: systematic review and trinational survey. Stroke 2010; 41(4):685-690.

(41) Counsell C, Dennis M, McDowall M, Warlow C. Predicting outcome after acute and subacute stroke: development and validation of new prognostic models. Stroke 2002; 33(4):1041-1047.

(42) Josephson CB, Frantzias J, Samarasekera N, Al-Shahi Salman R. The persisting burden of intracerebral haemorrhage: can effective treatments be found? PLoS Med 2010; 7(10):e1000353.

(43) Thygesen K, Alpert JS, Jaffe AS, Simoons ML, Chaitman BR, White HD et al. Third Universal Definition of Myocardial Infarction. Circulation. In press 2012.

(44) Bruno A, Shah N, Lin C, Close B, Hess DC, Davis K et al. Improving modified Rankin Scale assessment with a simplified questionnaire. Stroke 2010; 41(5):1048-1050.

(45) Bruno A, Akinwuntan AE, Lin C, Close B, Davis K, Baute V et al. Simplified modified rankin scale questionnaire: reproducibility over the telephone and validation with quality of life. Stroke 2011; 42(8):2276-2279.

(46) Dennis M, Mead G, Doubal F, Graham C. Determining the modified Rankin score after stroke by postal and telephone questionnaires. Stroke 2012; 43(3):851-853.

(47) Cordonnier C, Potter GM, Jackson CA, Doubal F, Keir S, Sudlow CL et al. Improving interrater agreement about brain microbleeds: development of the Brain Observer MicroBleed Scale (BOMBS). Stroke 2009; 40(1):94-99.

(48) Balami JS, Buchan AM. Complications of intracerebral haemorrhage. Lancet Neurol 2012; 11(1):101-118.

(49) Lovelock CE, Cordonnier C, Naka H, Al-Shahi SR, Sudlow CL, Sorimachi T et al. Antithrombotic drug use, cerebral microbleeds, and intracerebral hemorrhage: a systematic review of published and unpublished studies. Stroke 2010; 41(6):1222-1228.

(50) DAMOCLES Study Group. A proposed charter for clinical trial data monitoring committees: helping them to do their job well. Lancet 2005; 365(9460):711-722.

Restart or Stop Antithrombotics Randomised Trial (RESTART)

Final Analysis (Main analysis including extended follow up) - Statistical Analysis Plan

CONFIDENTIAL

**Version No**	2.0
**Date Finalised**	28th APR 2021
**Author(s)**	Jacqueline Stephen / Aryelly Rodriguez
**CI Name**	Rustam Al-Shahi Salman
**CI Email address**	rustam.al-shahi@ed.ac.uk

Signatures	
**Trial Statistician:**	**Date:**<br>28 April 2021
**Chief Investigator:**<br>Rustam Al-Shahi Salman	**Date:**<br>28 April 2021

**Document Control**		
**Version No**	**Date**	**Summary of Revisions**
1.0	06th APR 2021	Initial Statistical Analysis Plan (SAP) creation
2.0	28st APR 2021	During statistical validation it was detected that the outcome “All serious vaso-occlusive events including transient ischaemic attack and retinal arterial occlusion” was not included in the SAP. It is now included under 4.7.2.4. Under 4.7 Analysis of outcomes, the absolute difference in event rates has been taken to seven years.

Table of Contents

List of Abbreviations ... 3
1. Introduction ... 4
2. Statistical Methods section from the protocol ... 4
3. Overall Statistical Principles ... 4
3.1 Handling of missing data ... 5
4. List of Analyses ... 5
4.1 Recruitment and retention ... 5
4.2 Baseline data: demographics, baseline/clinical characteristics ... 5
4.3 Protocol deviations/violations ... 6
4.4 Completeness of follow-up ... 6
4.5 Adherence to allocated treatment ... 6
4.6 Co-interventions ... 6
4.7 Analysis of outcomes ... 6
4.7.1 Primary outcome ... 7
4.7.2 Secondary outcomes ... 7
4.7.2.1 All serious haemorrhagic events ... 7
4.7.2.2 All serious vaso-occlusive events (including revascularisation procedures) ... 7
4.7.2.3 Composite of all serious haemorrhagic or vaso-occlusive events ... 8
4.7.2.4 All serious vaso-occlusive events including transient ischaemic attack and retinal arterial occlusion ... 8
4.7.2.5 Major vascular events (as defined by the Antithrombotic Trialists' Collaboration) ... 8
4.7.3 Sensitivity analysis ... 8
4.7.4 Annual assessment of modified Rankin Scale (mRS) ... 8
4.7.5 Sub-group analyses ... 9
4.7.6 Serious adverse events ... 9
5. Validation and QC ... 9
6. Data Sharing ... 9
7. References ... 10

List of Abbreviations

Abbreviation	Full name
**BP**	Blood pressure
**CI**	Confidence interval
**ECTU**	Edinburgh Clinical Trials Unit
**ICH**	Intracerebral haemorrhage
**ITT**	Intention-to-treat
**mRS**	Modified Rankin Scale
**RESTART**	Restart or Stop Antithrombotics Randomised Trial
**SAP**	Statistical analysis plan
**SD**	Standard deviation
**SOP**	Standard operating procedure

1. Introduction

The Restart or Stop Antithrombotics Randomised Trial (RESTART) is a multicentre, randomised, open, assessor-blind, parallel group, clinical trial with 537 participants randomised 1:1 to start antiplatelet drug(s) (one or more of aspirin, clopidogrel, or dipyridamole, chosen by each participant's physician pre-randomisation) vs. avoid antiplatelet drugs. The primary objective is to estimate the relative and absolute effects of antiplatelet drugs on the risk of recurrent symptomatic intracerebral haemorrhage (ICH) 24h or more after the onset of spontaneous ICH.

Recruitment to the RESTART study ended on 30 May 2018 and its follow-up for the main results ended on 30 November 2018, followed by its publication in May 2019 [1] [2].

Extended follow-up of the RESTART study participants was endorsed by the Trial Steering Committee and authorised by Shannon Amoils (Senior Research Adviser at the British Heart Foundation, the trial's funder) on 25 February 2019, by the sponsor on 18 July 2019, and by the research ethics committee on 12 Jul 2019. Extended follow-up began with resumption of annual postal questionnaires to surviving participants and their general practitioners on 26 July 2019, and ended on 30 November 2020. This document details the criteria to be used for the analysis of the RESTART final results based on extended follow-up.

This document has been compiled according to Edinburgh Clinical Trials Unit (ECTU) standard operating procedure (SOP) "Statistical and Analysis Plans". This document has been written based on information contained in the final study protocol dated 19th September 2017, Version 8.0 and the final Statistical Analysis Plan dated 25th January 2019, Version 1.7. [3]

2. Statistical Methods section from the protocol

Our provisional analysis plan is described below, but we intend to publish a final Statistical Analysis Plan before the database is locked for analysis and the results are known. In order to preserve fully the huge benefit of randomisation, we will include all randomised participants in the analysis (irrespective of whether they adhere to the allocated treatment), all retained in the group to which they were allocated (i.e. "as-randomised"). This will comprise a Kaplan Meier survival analysis of time to first outcome event after randomisation. Follow-up will be censored at death (unrelated to an outcome event), last available follow-up, or voluntary withdrawal from the trial. We will compare the survival function in the two trial arms using a Cox proportional hazards regression model, adjusting for all the covariates included in the minimisation algorithm, and presenting the result as an estimated adjusted hazard ratio with its corresponding 95% CI. We will also report the unadjusted estimate of the hazard ratio and its corresponding 95% CI, together with the result of the logrank test.

3. Overall Statistical Principles

The statistician at ECTU will perform the statistical programming and analysis to produce all summary tables and figures using SAS software [4] (version 9.4 or later).

In general terms, categorical data will be presented using counts and percentages, whilst continuous variables will be presented using the mean, median, standard deviation (SD), minimum, maximum, inter quartile points at 25% and 75% (Q1 and Q3) and number of patients with an observation (n).

All applicable statistical tests will be 2-sided and will be performed using a 5% significance level, leading to 95% (2-sided) confidence intervals (CIs), unless otherwise specified.

All analyses will follow the “intention-to-treat (ITT)” principle with patients analysed according to the allocated intervention, irrespective of whether the patient’s actual management complied with the allocated intervention. The ITT population will comprise all patients who have been randomised into the RESTART study and for whom outcome data are available, regardless of whether they were subsequently deemed ineligible after independent review of their diagnostic brain imaging.

All analyses will include data collected from central follow-up including GP and patient questionnaires, but will not include outcome events identified through data linkage with secondary care data only.

3.1 Handling of missing data

There will be no imputation for the data with regard to missing values or withdrawals for the statistical summaries and statistical analysis unless otherwise specified.

Some of the covariates specified for the statistical analyses are required to inform the minimisation algorithm, and so there should be no missing data for these baseline covariates. Strenuous efforts are being made to obtain virtually complete outcome data.

Any required/applicable imputation of data will be performed at the discretion of the ECTU statistician. All imputations (if any) will be detailed in the statistical result report.

4. List of Analyses

4.1 Recruitment and retention

No formal statistical testing will be performed. A CONSORT flow diagram will be provided. The statistical report will tabulate the number of patients consented, randomised, treated, adherent, and completed follow-up overall and by treatment group. The number of patients discontinued early will be summarised by reason for withdrawal and treatment.

Regarding the number of patients consented, randomised, treated, the counting should be identical between the main and the final results reports.

4.2 Baseline data: demographics, baseline/clinical characteristics

No formal statistical testing will be performed. The following will be presented and summarised by treatment group and overall:

- the covariates used in the minimisation algorithm (qualifying ICH location; time since ICH symptom onset; antiplatelet drug(s) that patient’s physician would use if allocated; age; predicted six month outcome)

• sex; ethnicity; diagnostic imaging; MRI sub-study imaging; indicated uncertainty about starting antiplatelet drugs; functional status; modified Rankin Scale score; co-morbidities; antithrombotic drugs taken before ICH; timing of key events – symptom onset to randomisation – symptom onset to earliest imaging study – earliest imaging study to randomisation – symptom onset to sub-study MRI (if applicable) – sub-study MRI to randomisation (if applicable)

All the summaries in this section should be identical between the main and the final results reports.

4.3 Protocol deviations/violations

Any change, divergence, or departure from the trial design or procedures defined in the protocol or Good Clinical Practice are identified and recorded as a deviation (if it does not significantly affect a participant's rights, safety, or well-being, or trial outcomes), or a violation (if the deviation may potentially significantly impact the completeness, accuracy, and/or reliability of the trial data or that may significantly affect a patient's rights, safety, or well-being). All protocol deviations/violations will be listed, but no formal statistical testing will be performed

4.4 Completeness of follow-up

Completeness of follow-up will be reported as the proportion of participants with a complete follow-up questionnaire at each planned interval after randomisation, and as the proportion of the planned duration of follow-up that was observed.

4.5 Adherence to allocated treatment

No formal statistical testing will be performed. Adherence will be reported descriptively per treatment group at discharge and at each annual follow-up prior to the first outcome event.

4.6 Co-interventions

Use of anticoagulant drugs will be reported descriptively per treatment group at discharge and at each annual follow-up. Because blood pressure (BP) control is the other main confounder of the frequency of the primary outcome, the use of BP-lowering drugs at discharge after randomisation and at each annual follow-up will be reported (along with a summary of the available BPs of participants by treatment group).

4.7 Analysis of outcomes

The survival function per treatment group will be estimated using a Kaplan-Meier survival analysis of time to first outcome event during follow-up from randomisation. Follow-up will be censored at death (unrelated to an outcome event) or last available follow-up. We will compare the survival function in the two trial arms using a Cox proportional hazards regression model, adjusting for all the covariates included in the minimisation algorithm (Qualifying ICH location of lobar versus non-lobar; Time since ICH symptom onset of 0-6 days versus 7-30 days versus over 30 days; Antiplatelet drug(s) that the patient's clinician would start if allocated to aspirin alone versus any other regimen; Participant's age at randomisation of less than 70 years versus 70 years or older; Predicted probability of a good six month outcome of less than 0.15 versus 0.15 or greater), and presenting the result as an estimated adjusted hazard ratio with its corresponding 95% CI. We will also report the unadjusted estimate of

the hazard ratio and its corresponding 95% CI, together with the result of the logrank test. The absolute difference in event rates at one, two, three, four, five, six and seven years will be estimated from the Kaplan-Meier analysis.

The proportional hazards assumption will be assessed graphically, and if there is strong evidence of violation of the assumption the impact on the analysis will be assessed by comparing the results of the pre-specified analysis with the results obtained using the restricted survival time approach [5].

4.7.1 Primary outcome

The primary outcome event is the first fatal or non-fatal radiographically- or pathologically-proven recurrent symptomatic ICH.(Named: Recurrent ICH)

4.7.1.1. Sensitivity analyses

We will conduct two exploratory sensitivity analyses of our primary analysis, by adding the following secondary outcomes to the primary outcome in the following order, to account for the possibility that some fatalities and non-fatal neurological events without adequate investigation may be recurrent ICH:

- Fatal or non-fatal symptomatic stroke consistent with the clinical manifestations of ICH, but without radiographic or pathological confirmation (or with brain imaging performed too late to distinguish ICH from ischaemic stroke). (Named: Recurrent ICH including stoke of unknown type)
- Deaths without a clear cause and without further investigation. (Named: Recurrent ICH including stoke of unknown type and deaths of unknown cause)

4.7.2 Secondary outcomes

4.7.2.1 All serious haemorrhagic events

This composite outcome (named: All serious haemorrhagic events) includes all fatal or non-fatal symptomatic events (that are ‘serious’ because of their usual need for hospitalisation and influence on outcome and antithrombotic treatment):

- Radiographically- or pathologically-proven recurrent symptomatic ICH (the primary outcome)
- Other forms of symptomatic spontaneous or traumatic intracranial haemorrhage
    - Radiographically- or pathologically-proven spontaneous or traumatic extradural haemorrhage, subdural haemorrhage, subarachnoid haemorrhage, or intraventricular haemorrhage (not accompanying spontaneous ICH).
- Extracranial haemorrhage at any site requiring transfusion / endoscopic treatment / surgery, or resulting in death

4.7.2.2 All serious vaso-occlusive events (including revascularisation procedures)

This composite outcome (named: All serious vaso-occlusive events) includes fatal or non-fatal symptomatic events (that are ‘serious’ because of their usual need for hospitalisation and influence on outcome and antithrombotic treatment) or revascularisation procedures (we will not include transient ischaemic attack or retinal artery occlusion):

- Fatal or non-fatal vaso-occlusive events
  - Ischaemic stroke, acute coronary syndrome (unstable angina or myocardial infarction), mesenteric ischaemia or peripheral arterial occlusion.
  - Symptomatic deep vein thrombosis, pulmonary embolism.
- Carotid, coronary, or peripheral arterial revascularisation procedures.

4.7.2.3 Composite of all serious haemorrhagic or vaso-occlusive events

A further secondary analysis will be performed on the composite event (Named: All Serious haemorrhagic/vaso-occclusive events) which combines the two composites defined above, i.e. the composite of any serious haemorrhagic or vaso-occlusive event.

4.7.2.4 All serious vaso-occlusive events including transient ischaemic attack and retinal arterial occlusion

This composite outcome (named: All serious vaso-occlusive events including transient ischaemic attack and retinal arterial occlusion) includes “all serious vaso-occlusive events” (as defined in 4.7.2.2 on this document) or transient ischaemic attack or retinal arterial occlusion events.

4.7.2.5 Major vascular events (as defined by the Antithrombotic Trialists' Collaboration)

For completeness, the corresponding analysis will also be performed for the composite outcome proposed in the trial protocol, namely all major vascular events defined by the Antithrombotic Trialists' Collaboration (non-fatal myocardial infarction, non-fatal stroke [ischaemic, haemorrhagic, or uncertain cause], or death from a vascular cause) [6] [7]. (Name: Serious vascular events)

The interpretation of the trial findings will respect this prespecified hierarchy of primary outcome, key secondary outcomes and other secondary outcomes, and no formal adjustment will be made to significance levels to allow for multiplicity.

4.7.3 Sensitivity analysis

A sensitivity analysis will be performed to reflect the cumulative incidence of serious haemorrhagic or vaso-occlusive events. This analysis will use re-randomisation tests, as described by Ford et al. [8]

4.7.4 Annual assessment of modified Rankin Scale (mRS)

A separate analysis will be performed for each annual assessment. The analysis at year ‘x’ will be restricted to those recruits who were randomised at least ‘x’ years prior to extended follow up end, to avoid including early deaths in the relevant follow-up year when the corresponding surviving recruits would not have had the potential to be assessed. The analysis will comprise a tabulation of mRS by randomised group, with the formal analysis being based on a Mann-Whitney test. Participants’ type of domicile will be described as categorised on the discharge form and each annual participant questionnaire.

4.7.5 Sub-group analyses

A priori sub-groups for the primary analysis will be explored as follows:

- The five covariates used in the minimisation algorithm
- History of atrial fibrillation at randomisation
- Pre-ICH antithrombotic drug regimen (antiplatelet alone versus anticoagulant with or without antiplatelet)

These sub-group analyses will be applied to the primary outcome and the key secondary outcomes: (i) all serious haemorrhagic events, (ii) all serious vaso-occlusive events (including revascularisation procedures), (iii) the composite of all serious haemorrhagic or vaso-occlusive events, (iv) All serious vaso-occlusive events including transient ischaemic attack and retinal arterial occlusion and (v) major vascular events defined by the Antithrombotic Trialists' Collaboration.[1]

These analyses will be performed by including an interaction term between treatment group and the relevant covariate in the Cox proportional hazards regression model described in Section 4.7 above. For the subgroup analysis relating to time since ICH symptom onset, two subgroups will be defined based on the time from onset being above or below the median time observed in the trial.

4.7.6 Serious adverse events

Serious adverse events (SAEs) are reported in RESTART if they not outcome events or expected complications of stroke. SAEs will be grouped by body system and for each event and each grouped set of events the number of events and the number of individuals experiencing at least one such event will be tabulated per randomised group

5. Validation and QC

QC/Validation of statistical analysis and report will be performed by statistical peer review of program code, log and output.

6. Data Sharing

A set of files, containing the final data for the RESTART study up will be prepared, along with their corresponding data dictionary. These will be made available to the Chief Investigator at the end of the analysis phase and located at the RESTART area set up on Datashare (https://datashare.ed.ac.uk/handle/10283/3265).

Ownership of the data arising from this study resides with the Trial Steering Committee. Access to the datasets generated and/or analysed during RESTART will be available on reasonable request after the publication of the final results. Access will be controlled by the chief investigator, with the approval of the Trial Steering Committee.

7. References

[1] RESTART Collaboration. Effects of antiplatelet therapy after stroke due to intracerebral haemorrhage (RESTART): a randomised, open-label trial. The Lancet 2019;393:2613-23

[2] Al-Shahi Salman R, Minks DP, Mitra D, Rodrigues MA, Bhatnagar P, du Plessis JC, Joshi Y, Dennis MS, Murray GD, Newby DE, Sandercock PAG, Sprigg N, Stephen J, Sudlow CLM, Werring DJ, Whiteley WN, Wardlaw JM, White PM on behalf of the RESTART Collaboration. Effects of antiplatelet therapy on stroke risk by brain imaging features of intracerebral haemorrhage and cerebral small vessel diseases: sub-group analyses of the RESTART randomised, open-label trial. The Lancet Neurology 2019;18:643-52

[3] Al-Shahi Salman R, Murray, G. D., Dennis, M. S., Newby, D. E., Sandercock, P. A., Sprigg, N., ... & Whiteley, W. N. (2019). The REstart or STop Antithrombotics Randomised Trial (RESTART) after stroke due to intracerebral haemorrhage: statistical analysis plan for a randomised controlled trial. Trials, 20(1), 1-8.

[4] SAS® Institute Inc. SAS for Windows. SAS Institute Inc.: Cary, NC, U.S.A

[5] Royston P, Parmar MK. The use of restricted mean survival time to estimate the treatment effect in randomized clinical trials when the proportional hazards assumption is in doubt. *Statistics in Medicine* 2011;30(19):2409-21

[6] Baigent C, Blackwell L et al. Antithrombotic Trialists' (ATT) Collaboration. Aspirin in the primary and secondary prevention of vascular disease: collaborative meta-analysis of individual participant data from randomised trials. Lancet 2009; 373: 1849-1860

[7] Al-Shahi Salman R, Dennis MS, Murray GD et al. The REstart or STop Antithrombotics Randomised Trial (RESTART) after stroke due to intracerebral haemorrhage: study protocol for a randomised controlled trial. Trials 2018; 19: 162

[8] Ford I, Murray H, McCowan C, Packard CJ. Long-Term Safety and Efficacy of Lowering Low-Density Lipoprotein Cholesterol With Statin Therapy: 20-Year Follow-Up of West of Scotland Coronary Prevention Study. *Circulation* 2016;133(11):1073-80
