# Supplementary material for: Effects of Antiplatelet Therapy After Stroke Caused by Intracerebral Hemorrhage: Extended Follow-up of the RESTART Randomized Clinical Trial
Source: JAMA Neurol. 2021 Sep 3;78(10):1–8. doi: 10.1001/jamaneurol.2021.2956 (PMC8417806; doi:10.1001/jamaneurol.2021.2956)
Supplement: Supplement 3. — Nonauthor Collaborators. RESTART Collaboration [file jamaneurol-e212956-s003.pdf]

Supplemental Online Content: Nonauthor Collaborators

*Indicates required information. Only first name, last name, and suffix will appear in PubMed.

Group Name:* Members of the RESTART Collaboration

First Name, Middle Initial*	Last Name*	Suffix*	Academic Degrees	Institution	Location (city, state/province, country)	Role or Contribution, eg, chair, principal investigator	Subgroup, eg, Steering Committee
Colin	Baigent			University of Oxford	Oxford, UK	Independent chairperson	Trial steering committee
Daniel	Lasserson			University of Warwick	Warwick, UK	Independent member	Trial steering committee
Frank	Sullivan			University of St Andrews	St Andrews, UK	Independent member	Trial steering committee
Johanna	Carrie					Independent patient representative	Trial steering committee
David	Newby			University of Edinburgh	Edinburgh, UK	Member	Trial steering committee
Nikola	Sprigg			University of Nottingham	Nottingham, UK	Member	Trial steering committee
Javier	Rojas			Academic and Clinical Central Office for Research and Development (ACCORD)	Edinburgh, UK	Sponsor representative	Trial steering committee
Bernadette	Gallagher			Academic and Clinical Central Office for Research and Development (ACCORD)	Edinburgh, UK	Sponsor representative	
Elizabeth	Brownsell			Academic and Clinical Central Office for Research and Development (ACCORD)	Edinburgh, UK	Sponsor representative	

Supplemental Online Content: Nonauthor Collaborators

*Indicates required information. Only first name, last name, and suffix will appear in PubMed.

First Name, Middle Initial*	Last Name*	Suffix*	Academic Degrees	Institution	Location (city, state/province, country)	Role or Contribution, eg, chair, principal investigator	Subgroup, eg, Steering Committee
Paul	Dearie			Academic and Clinical Central Office for Research and Development (ACCORD)	Edinburgh, UK	Clinical Research Facilitation Manager	
Alice	Graves			Academic and Clinical Central Office for Research and Development (ACCORD)	Edinburgh, UK		
Louisa	Wilson			Academic and Clinical Central Office for Research and Development (ACCORD)	Edinburgh, UK		
Gelnna	Hamer			Academic and Clinical Central Office for Research and Development (ACCORD)	Edinburgh, UK		
Elizabeth	Craig			Academic and Clinical Central Office for Research and Development (ACCORD)	Edinburgh, UK		
Priscilla	Sauramba			Academic and Clinical Central Office for Research and Development (ACCORD)	Edinburgh, UK		

Supplemental Online Content: Nonauthor Collaborators

*Indicates required information. Only first name, last name, and suffix will appear in PubMed.

First Name, Middle Initial*	Last Name*	Suffix*	Academic Degrees	Institution	Location (city, state/province, country)	Role or Contribution, eg, chair, principal investigator	Subgroup, eg, Steering Committee
Nicola	Lyttle			Academic and Clinical Central Office for Research and Development (ACCORD)	Edinburgh, UK		
Shannon	Amoils			British Heart Foundation	London, UK	Funder representative	Trial steering committee
John	Bamford			University of Leeds	Leeds, UK	Chairperson	Data monitoring committee
Jane	Armitage			University of Oxford	Oxford, UK	Member	Data monitoring committee
Gabriel	Rinkel			UMC Utrecht	Utrecht, The Netherlands	Member	Data monitoring committee
Gordon	Lowe			University of Glasgow	Glasgow, UK	Member	Data monitoring committee
Jonathan	Emberson			University of Oxford	Oxford, UK	Member	Data monitoring committee
Karen	Innes			University of Edinburgh	Edinburgh, UK	Senior Trial Manager	
Lynn	Dinsmore			University of Edinburgh	Edinburgh, UK	Imaging Manager	
Jonathan	Drever			University of Edinburgh	Edinburgh, UK	Data Manager	
Carol	Williams			University of Edinburgh	Edinburgh, UK	Centre Coordinator	
David	Perry			University of Edinburgh	Edinburgh, UK	Database programmer	
Connor	McGill			University of Edinburgh	Edinburgh, UK	Database programmer	
David	Buchanan			University of Edinburgh	Edinburgh, UK	Database programmer	

Supplemental Online Content: Nonauthor Collaborators

*Indicates required information. Only first name, last name, and suffix will appear in PubMed.

First Name, Middle Initial*	Last Name*	Suffix*	Academic Degrees	Institution	Location (city, state/province, country)	Role or Contribution, eg, chair, principal investigator	Subgroup, eg, Steering Committee
Allan	Walker			University of Edinburgh	Edinburgh, UK	Database programmer	
Aidan	Hutchison			University of Edinburgh	Edinburgh, UK	Database programmer	
Christopher	Matthews			University of Edinburgh	Edinburgh, UK	Database programmer	
Ruth	Fraser			University of Edinburgh	Edinburgh, UK	Trial Support Officer	
Aileen	McGrath			University of Edinburgh	Edinburgh, UK	Trial Support Officer	
Ann	Deary			University of Edinburgh	Edinburgh, UK	Trial Support Officer	
Rosemary	Anderson			University of Edinburgh	Edinburgh, UK	Trial Support Officer	
Pauli	Walker			University of Edinburgh	Edinburgh, UK	Trial Support Officer	
Kasia	Adamczuk			University of Edinburgh	Edinburgh, UK	SoSTART Trial Manager	
Christian	Hansen			University of Edinburgh	Edinburgh, UK	Unblinded independent statistician	
Richard	Parker			University of Edinburgh	Edinburgh, UK	Unblinded independent statistician	
Robert	Lee			University of Edinburgh	Edinburgh, UK	Unblinded independent statistician	
Malcolm	Macleod			University of Edinburgh	Edinburgh, UK	Outcome event internal adjudicator	
Thomas	Moullaali			University of Edinburgh	Edinburgh, UK	Outcome event internal adjudicator	

Supplemental Online Content: Nonauthor Collaborators

*Indicates required information. Only first name, last name, and suffix will appear in PubMed.

First Name, Middle Initial*	Last Name*	Suffix*	Academic Degrees	Institution	Location (city, state/province, country)	Role or Contribution, eg, chair, principal investigator	Subgroup, eg, Steering Committee
Jeb	Palmer			University of Edinburgh	Edinburgh, UK	Programmer	
Eleni	Sakka			University of Edinburgh	Edinburgh, UK	Imaging Manager	
Jennifer	Adil-Smith			University of Edinburgh	Edinburgh, UK	Imaging Manager	
David	Minks			Newcastle-upon-Tyne Hospitals NHS Trust	Newcastle-upon-Tyne, UK	Brain imaging assessor	
Dipayan	Mitra			Newcastle-upon-Tyne Hospitals NHS Trust	Newcastle-upon-Tyne, UK	Brain imaging assessor	
Priya	Bhatnagar			Newcastle-upon-Tyne Hospitals NHS Trust	Newcastle-upon-Tyne, UK	Brain imaging assessor	
Johannes	du Plessis			NHS Lothian	Edinburgh, UK	Brain imaging assessor	
Yogish	Joshi			Addenbrooke's Hospital	Cambridge, UK	Brain imaging assessor	
Kelly	Stewart			University of Edinburgh	Edinburgh, UK	Trial Support Officer	
Kate	Covil			University of Edinburgh	Edinburgh, UK	Research nurse	
Mark	Rodrigues			University of Edinburgh	Edinburgh, UK	Brain imaging assessor	
Christine	Lerpiniere						
Richard	O’Brien						
Seona	Burgess						
Gillian	Mead						
Ruth	Paulton						
Fergus	Doubal						
Katrina	McCormick						
Neil	Hunter						
Pat	Taylor						

*Indicates required information. Only first name, last name, and suffix will appear in PubMed.

First Name, Middle Initial*	Last Name*	Suffix*	Academic Degrees	Institution	Location (city, state/province, country)	Role or Contribution, eg, chair, principal investigator	Subgroup, eg, Steering Committee
Ruwan	Parakramawansha						
Jack	Perry						
Gordon	Blair						
Allan	MacRaild						
Adrian	Parry-Jones						
Mary	Johnes						
Stephanie	Lee						
Kelly Marie	Shaw						
Ilse	Burger						
Martin	Punter						
Andrea	Ingham						
Jane	Perez						
Zin	Naing						
Jordi	Morell						
Tracy	Marsden						
Andrea	Hall						
Sally	Marshall						
Louise	Harrison						
Rowilson	Jarapa						
Edith	Wood						
Victoria	O'Loughlin						
David	Cohen						
Silvie	Davies						
Kelechi	Njoku						
Mushiya	Mpelembue						
Laura	Burgess						
Radim	Licenik						
Mmua	Ngwako						
Nabeela	Nisar						

*Indicates required information. Only first name, last name, and suffix will appear in PubMed.

First Name, Middle Initial*	Last Name*	Suffix*	Academic Degrees	Institution	Location (city, state/province, country)	Role or Contribution, eg, chair, principal investigator	Subgroup, eg, Steering Committee
Rangah	Niranchanan						
Tatjana	Roganova						
Rajaram	Bathula						
Joseph	Devine						
Anette	David						
Anne	Oshodi						
Fenglin	Guo						
Emmanuelle	Owoyele						
Varthi	Sukdeo						
Robert	Ballantine						
Mudhar	Abbdul-Saheb						
Angela	Chamberlain						
Aberami	Chandrakumar						
Philip	Poku						
Kirsty	Harkness						
Emma	Richards						
Ali	Ali						
Catrin	Blank						
Faith	Kibutu						
Olesia	Balitska						
Kathryn	Birchall						
Pauline	Bayliss						
Kathy	Stocks						
Arshad	Majis						
Clare	Doyle						
Jo	Howe						
Christine	Kamara						
Luke	Barron						
Ahmad	Maatouk						

*Indicates required information. Only first name, last name, and suffix will appear in PubMed.

First Name, Middle Initial*	Last Name*	Suffix*	Academic Degrees	Institution	Location (city, state/province, country)	Role or Contribution, eg, chair, principal investigator	Subgroup, eg, Steering Committee
Ralf	Lindert						
Katy	Dakin						
Jessica	Redgrave						
John	France						
Dawn	Tomlin						
Helen	Bearne						
Isam	Salih						
Debs	Kelly						
Susan	Szabo						
Biju	Bhaskaran						
Jean	Buxton						
Pauline	Fitzell						
Georgina	Ayres						
Afaq	Saulat						
Kathleen	Horan						
Joanne	Garfield-Smith						
Harbens	Bhakri						
Stacey	Atkins						
Joan	Redome						
Paul	Guyler						
Thayalini	Loganathan						
Amber	Siddiqui						
Anwer	Siddiqui						
Lucy	Coward						
Devesh	Sinha						
Swapna	Kunhunny						
Sharon	Tysoe						
Rajalakshmi	Orath Prabakaran						
Shyam	Kelavkar						

*Indicates required information. Only first name, last name, and suffix will appear in PubMed.

First Name, Middle Initial*	Last Name*	Suffix*	Academic Degrees	Institution	Location (city, state/province, country)	Role or Contribution, eg, chair, principal investigator	Subgroup, eg, Steering Committee
Sindhu	Rashmi						
David	Ngo						
Kheng Xiong	Ng						
Nisha	Menon						
Sweni	Shah						
Mark	Barber						
Derek	Esson						
Fiona	Brodie						
Talat	Anjum						
Manju	Krishnan						
Leanne	Quinn						
Jayne	Spencer						
Terry	Jones						
Helen	Thompson Jones						
Srikanth	Chenna						
Sharon	Storton						
Lynne	Dacey						
Sarah	Thomas						
Teresa	Beaty						
Mushtaq	Wani						
Shelley	Treadwell						
Caroline	Davies						
Susan	Tucker						
Lynda	Connor						
Glyn	Gainard						
Peter	Slade						
Girish	Muddegowda						
Nenette	Abano						
Alda	Remegoso						

*Indicates required information. Only first name, last name, and suffix will appear in PubMed.

First Name, Middle Initial*	Last Name*	Suffix*	Academic Degrees	Institution	Location (city, state/province, country)	Role or Contribution, eg, chair, principal investigator	Subgroup, eg, Steering Committee
Racquel	Carpio						
Chelsea	Causley						
Stephanie	Stevens						
Adrian	Butler						
Resti	Varquez						
Andrew	Moores						
Francis	Alipio						
Hayley	Denic						
Ranjan	Sanyal						
Adrian	Barry						
Holly	Maguire						
Jeanette	Grocott						
Kay	Finney						
Sue	Lyjko						
Christine	Roffe						
Joanne	Hiden						
Phillip	Ferdinand						
Vera	Cvoro						
Khalil	Ullah						
Nicola	Chapman						
Mandy	Couser						
Susan	Pound						
Katrina	McCormick						
Sean	Mcauley						
Senthil	Raghunathan						
Faye	Shelton						
Amanda	Hedstrom						
Margi	Godfrey						
Diane	Havard						

Supplemental Online Content: Nonauthor Collaborators

*Indicates required information. Only first name, last name, and suffix will appear in PubMed.

First Name, Middle Initial*	Last Name*	Suffix*	Academic Degrees	Institution	Location (city, state/province, country)	Role or Contribution, eg, chair, principal investigator	Subgroup, eg, Steering Committee
Amanda	Buck						
Kailash	Krishnan						
Nicola	Gilzeane						
Jack	Roffe						
Judith	Clarke						
Saima	Sheikh						
Nikola	Sprigg						
Katherine	Whittamore						
Rekha	Keshvara						
Carla	Jordan						
Gwendoline	Wilkes						
Benjamin	Jackson						
Jason	Appleton						
Zhe	Law						
Oliver	Matias						
Evangelos	Vasileiadis						
Cathy	Mason						
Anthea	Parry						
Geraldine	Landers						
Melinda	Holden						
Basaam	Aweid						
Khalid	Rashed						
Linda	Balian						
Carinna	Vickers						
Elizabeth	Keeling						
Sarah	Board						
Joanna	Allison						
Clare	Buckley						
Barbara	Williams-Yesson						

*Indicates required information. Only first name, last name, and suffix will appear in PubMed.

First Name, Middle Initial*	Last Name*	Suffix*	Academic Degrees	Institution	Location (city, state/province, country)	Role or Contribution, eg, chair, principal investigator	Subgroup, eg, Steering Committee
Joanne	Board						
Tressy	Pitt-Kerby						
Alfonso	Tanate						
Diane	Wood						
Dinesh	Chadha						
Manohar	Kini						
Deborah	Walstow						
Rosanna	Fong						
Robert	Luder						
Tolu	Adesina						
Jill	Gallagher						
Hayley	Bridger						
Elodie	Murali						
Larissa	Cuenoud						
Maneesh	Bhargava						
Chloe	Van Someren						
Katja	Adie						
Gillian	Courtauld						
Christine	Schofield						
Abhijit	Mate						
Ali	James						
Frances	Harrington						
Linda	Lucas						
Kirsty	Bond						
Bev	Maund						
Sam	Ellis						
Emilie	Williams						
Sue	Webber						
Terri	Chant						

Supplemental Online Content: Nonauthor Collaborators

*Indicates required information. Only first name, last name, and suffix will appear in PubMed.

First Name, Middle Initial*	Last Name*	Suffix*	Academic Degrees	Institution	Location (city, state/province, country)	Role or Contribution, eg, chair, principal investigator	Subgroup, eg, Steering Committee
Elizabeth	Swanson						
Eleanor	King						
Abbie	Coutts						
Nicola	Yeo						
Paul	Mudd						
Martin	James						
Hayley	Kingwell						
Caroline	Roughan						
Anthony	Hemsley						
Jane	Sword						
Samantha	Keenan						
Angela	Bowring						
Julie	Cageao						
David	Strain						
Keniesha	Miller						
Anita	Goff						
Karin	Gupwell						
Kevin	Thorpe						
Hedley	Emsley						
Alison	McLoughlin						
Sulaiman	Sultan						
Shuja	Punekar						
Bindu	Gregory						
Sonia	Raj						
Donna	Doyle						
Keith	Muir						
Wilma	Smith						
Fiona	Moreton						
Bharath Kumar	Cheripelli						

Supplemental Online Content: Nonauthor Collaborators

*Indicates required information. Only first name, last name, and suffix will appear in PubMed.

First Name, Middle Initial*	Last Name*	Suffix*	Academic Degrees	Institution	Location (city, state/province, country)	Role or Contribution, eg, chair, principal investigator	Subgroup, eg, Steering Committee
Salwa	El Tawil						
Dheeraj	Kalladka						
Angela	Welch						
Xuya	Huang						
Nicola	Day						
Sankaranarayanan	Ramachandran						
Caroline	Crosbie						
Jennifer	Elliot						
Peter	Sommerville						
Meegan	Gibbons						
Rowshanara	Ahmed						
Sagal	Kullane						
Katherine	Marks						
Ajay	Bhalla						
Jonathan	Birns						
Tony	Rudd						
Nic	Weir						
Christopher	Allen						
Vanessa	Pressly						
Pam	Crawford						
Emma	Battersby-Wood						
Alex	Blades						
Shuna	Egerton						
Ashleigh	Walters						
James Richard	Marigold						
Sue	Evans						
Fiona	Smith						
Gabriella	Howard						
Imogen	Gartrell						

*Indicates required information. Only first name, last name, and suffix will appear in PubMed.

First Name, Middle Initial*	Last Name*	Suffix*	Academic Degrees	Institution	Location (city, state/province, country)	Role or Contribution, eg, chair, principal investigator	Subgroup, eg, Steering Committee
Simon	Smith						
Chloe	Cox						
Robyn	Creeden						
Cherish	Boxall						
Jonathan	Hewitt						
Claire	Nott						
Procter	Sarah						
Jessica	Whiteman						
Steve	Buckle						
Rina	Mardania						
Jane	Gray						
Rebecca	Wallace						
Claire	Triscott						
Anand	Nair						
Jill	Greig						
Pratap	Rana						
Matthew	Robinson						
Mohammad Irfan	Alam						
Ridha	Ramiz						
David	Werring						
Duncan	Wilson						
Caroline	Watchurst						
Maria	Brezitski						
Ifan	Jones						
Luci	Crook						
Azra	Banaras						
Krishna	Patel						
Renuka	Erande						
Caroline	Hogan						

*Indicates required information. Only first name, last name, and suffix will appear in PubMed.

First Name, Middle Initial*	Last Name*	Suffix*	Academic Degrees	Institution	Location (city, state/province, country)	Role or Contribution, eg, chair, principal investigator	Subgroup, eg, Steering Committee
Talal	Al-Mayhani						
Isabel	Hostettler						
Amy	Ashton						
Shez	Feerick						
Nina	Francia						
Nnebuife	Oji						
Emma	Elliott						
Martin	Dennis						
William	Whiteley						
Cathie	Sudlow						
Christine	Lerpiniere						
Dipankar	Dutta						
Pauline	Brown						
Deborah	Ward						
Fiona	Davis						
Jennifer	Turfrey						
Chloe	Hughes						
Kayleigh	Collins						
Rehana	Bakawala						
Susan	O'Connell						
Jon	Glass						
David	Broughton						
Lynn	Dixon						
Kath	Chapman						
Andrew	Young						
Adrian	Bergin						
Dinesh	Tryambake						
Andrew	Sigsworth						
Aravind	Manoj						

Supplemental Online Content: Nonauthor Collaborators

*Indicates required information. Only first name, last name, and suffix will appear in PubMed.

First Name, Middle Initial*	Last Name*	Suffix*	Academic Degrees	Institution	Location (city, state/province, country)	Role or Contribution, eg, chair, principal investigator	Subgroup, eg, Steering Committee
Paula	Lopez						
Penelope	Cox						
Glyn	Fletcher						
Mark	Wilkinson						
Paul	Fitzsimmons						
Nikhil	Sharma						
James	Choulerton						
Denise	Button						
Lindsey	Dow						
Lukuman	Gbadamoshi						
Joanne	Avis						
Barbara	Madigan						
Mccann	Stephanie						
Louise	Shaw						
Deborah	Howcroft						
Suzanne	Lucas						
Andrew	Stone						
Gillian	Cluckie						
Brian	Clarke						
Natasha	Clarke						
Neha	Chopra						
Bhavini	Patel						
Kate	Kennedy						
Rebecca	Williams						
Adrian	Blight						
Joanna	Oreilly						
Chukwuka	Orefo						
Nilofer	Dayal						
Rita	Ghatala						

*Indicates required information. Only first name, last name, and suffix will appear in PubMed.

First Name, Middle Initial*	Last Name*	Suffix*	Academic Degrees	Institution	Location (city, state/province, country)	Role or Contribution, eg, chair, principal investigator	Subgroup, eg, Steering Committee
Temi	Adedoyin						
Caroline	Lovelock						
Fran	Watson						
Sarah	Trippier						
Lillian	Choy						
Barry	Moynihan						
Usman	Khan						
Val	Jones						
Naomi	Jeyaraj						
Lourda	Kerin						
Kamy	Thavanesan						
Chantel	Cox						
Arshi	Iqbal						
Caroline	Bagnall						
Anja	Ljubez						
Marketa	Keltos						
Josh	Roberts						
Becky	Jupp						
Laura	Tucker						
Tanith	Changuion						
Catherine	Ovington						
Emily	Rogers						
Owen	David						
Jo	Bell						
Barbara	Longland						
Gail	Hann						
Divya	Tiwari						
Martin	Cooper						
Mohammad	Nasar						

*Indicates required information. Only first name, last name, and suffix will appear in PubMed.

First Name, Middle Initial*	Last Name*	Suffix*	Academic Degrees	Institution	Location (city, state/province, country)	Role or Contribution, eg, chair, principal investigator	Subgroup, eg, Steering Committee
Anoja	Rajapakse						
Inez	Wynter						
Ijaz	Anwar						
Helen	Skinner						
Tarn	Nozedar						
Damian	Mcardle						
Balakrishna	Kumar						
Susan	Crawford						
Arunkumar	Annamalai						
Alex	Ramshaw						
Clare	Holmes						
Mairead	Osborn						
Emily	Dodd						
Peter	Murphy						
Nicola	Devitt						
Pauline	Baker						
Amy	Steele						
Lucy Belle	Guthrie						
Sarah	Caine						
Samantha	Clarke						
Ahamad	Hassan						
Dean	Waugh						
Emelda	Veraque						
Linetty	Makawa						
Mary	Kambafwile						
Marc	Randall						
Vasileios	Papavasileiou						
Claire	Cullen						
Jenny	Peters						

*Indicates required information. Only first name, last name, and suffix will appear in PubMed.

First Name, Middle Initial*	Last Name*	Suffix*	Academic Degrees	Institution	Location (city, state/province, country)	Role or Contribution, eg, chair, principal investigator	Subgroup, eg, Steering Committee
Hlaing	Thant						
Tanya	Ingram						
Mellor	Zoe						
Ramesh	Durairaj						
Melanie	Harrison						
Daniela	Shackcloth						
Jordan	Ewing						
Sarah	Stevenson						
Victoria	Sutton						
Mark	McCarron						
Jacqueline	McKee						
Mandy	Doherty						
Ferghal	McVerry						
Caroline	Blair						
Donal	Concannon						
Mary	Macleod						
Janice	Irvine						
Heather	Gow						
Jacqueline	Furnace						
Anu	Joyson						
Baljit	Jagpal						
Sarah	Ross						
Katrina	Klaasen						
Beverly	Maclennan						
Sandra	Nelson						
Rebecca	Clarke						
Nichola	Crouch						
Vicky	Taylor						
Daniel	Epstein						

*Indicates required information. Only first name, last name, and suffix will appear in PubMed.

First Name, Middle Initial*	Last Name*	Suffix*	Academic Degrees	Institution	Location (city, state/province, country)	Role or Contribution, eg, chair, principal investigator	Subgroup, eg, Steering Committee
Avani	Shukla						
Ifan	Jones						
Vinodh	Krishnamurthy						
Paul	Nicholas						
Sammie	Qureshi						
Adam	Webber						
Justin	Penge						
Hawraman	Ramadan						
Stuart	Maguire						
Chris	Patterson						
Ruth	Bellfield						
Brigid	Hairsine						
Kelvin	Stewart						
Michaela	Hooley						
Outi	Quinn						
Bella	Richard						
Sally	Moseley						
Claire	Nott						
Steve	Buckle						
Procter	Sarah						
Jessica	Whiteman						
Mandy	Edwards						
Heidi	Lawson						
Rebecca	Wallace						
Claire	Triscott						
Michelle	Tayler						
Yogish	Pai						
Gemma Marie	Smith						
Mark	Garside						

*Indicates required information. Only first name, last name, and suffix will appear in PubMed.

First Name, Middle Initial*	Last Name*	Suffix*	Academic Degrees	Institution	Location (city, state/province, country)	Role or Contribution, eg, chair, principal investigator	Subgroup, eg, Steering Committee
Mahesh	Dhakal						
Muhammad	Naeem						
Vidya	Baliga						
Gill	Rogers						
Ellen	Brown						
David	Bruce						
Susan	Clayton						
Rachel	Hayman						
Sofia	Dima						
Ed	Gamble						
Rebecca	Grue						
Bethan	Charles						
Adam	Hague						
Sujata	Blane						
Caroline	Lambert						
Afnan	Chaudhry						
Thomas	Harrison						
Laura	Howaniec						
Dionne	Hove						
Gemma	Grimwood						
Kari	Saastamoinen						
Ozlem	Redjep						
Fiona	Humphries						
Lucia	Argandona						
Esther	Erumere						
Sageet	Amlani						
Grace	Auld						
Afraim	Salek-Haddadi						
Ursula	Schulz						

Supplemental Online Content: Nonauthor Collaborators

*Indicates required information. Only first name, last name, and suffix will appear in PubMed.

First Name, Middle Initial*	Last Name*	Suffix*	Academic Degrees	Institution	Location (city, state/province, country)	Role or Contribution, eg, chair, principal investigator	Subgroup, eg, Steering Committee
Philip	Mathieson						
Ian	Reckless						
James	Kennedy						
Gary	Ford						
Rachel	Teal						
Giulia	Lenti						
George	Harston						
Eoin	Obrien						
Sarah	Finlay						
Helen	Hayhoe						
Sarah	Crisp						
Juliana	Sesay						
George	Zachariah						
Jobbin	Francis						
Dominic	Handley						
Siobhan	Kelly						
Joanne	Mcgee						
Jennifer	Mitchell						
Elaine	Amis						
Niamh	Hannon						
Tom	Hughes						
Bethan	Morse						
Henry	De Berker						
Susan	White						
Emma	Tallantyre						
Ahmed	Osman						
Lucy	Knibbs						
Stefan	Schwarz						
Benjamin	Jelley						

*Indicates required information. Only first name, last name, and suffix will appear in PubMed.

First Name, Middle Initial*	Last Name*	Suffix*	Academic Degrees	Institution	Location (city, state/province, country)	Role or Contribution, eg, chair, principal investigator	Subgroup, eg, Steering Committee
Rajendra	Yadava						
Julie	Reddan						
Mirriam	Sangombe						
Khalid	Azhar						
Samantha	Stafford						
Nasar	Ahmad						
Ken	Fotherby						
Debbie	Morgan						
Farrukh	Baig						
Karla	Jennings-Preece						
Donna	Butler						
Baljinder	Rai						
Angela	Willberry						
Angela	Stevens						
Prasad	Siddegowda						
Afaq	Saulat						
Lisa	Hyatt						
Peter	Howard						
David	Jarrett						
Suheil	Ponnambath						
Tracey	Dobson						
Jane	Tandy						
Claire	James						
Stacey	Valentine						
Yasmin	Harrington-Davies						
Rebecca	Butler						
Anne	Suttling						
Fiona	Wright						
Gillian	Kerr						

Supplemental Online Content: Nonauthor Collaborators

*Indicates required information. Only first name, last name, and suffix will appear in PubMed.

First Name, Middle Initial*	Last Name*	Suffix*	Academic Degrees	Institution	Location (city, state/province, country)	Role or Contribution, eg, chair, principal investigator	Subgroup, eg, Steering Committee
Ruth	Graham						
Christine	McAlpine						
Peter	Langhorne						
Mohammad Shahz	Iqbal						
Louise	Humphreys						
Kath	Pasco						
Olga	Balazikova						
Ashraf	Nasim						
Cassilda	Peixoto						
Louise	Gallagher						
Shahrzad	Shahmehri						
Sandip	Ghosh						
Elizabeth	Barrie						
Danielle	Gilmour						
Margo	Henry						
Tom	Webb						
Linda	Cowie						
Hannah	Rudenko						
Natasha	Schumacher						
Shanni	Mcdonald						
Susannah	Walker						
Tracey	Cosier						
Anna	Verrion						
Eva	Beranova						
Audrey	Thomson						
Marius	Venter						
Sheila	Mashate						
Kirsten	Harvey						
Léjeune	Gardener						

Supplemental Online Content: Nonauthor Collaborators

*Indicates required information. Only first name, last name, and suffix will appear in PubMed.

First Name, Middle Initial*	Last Name*	Suffix*	Academic Degrees	Institution	Location (city, state/province, country)	Role or Contribution, eg, chair, principal investigator	Subgroup, eg, Steering Committee
Vinh	Nguyen						
Omid	Halse						
Olivia	Geraghty						
Beth	Hazel						
Peter	Wilding						
Victoria	Tilley						
Arindam	Kar						
Louise	Southern						
Tim	Cassidy						
Bernard	Esisi						
Beverley	McClelland						
Maria	Bokhari						
Timothy	England						
Amanda	Hedstrom						
Mohana	Maddula						
Richard	Donnelly						
Paul	Findlay						
Ian	Shread						
Ashish	Macaden						
Charlotte	Barr						
Azlisham	Mohd Nor						
Nicola	Persad						
Charlotte	Eglinton						
Marie	Weinling						
Claire	Brown						
Benjamin	Hyams						
Alex	Shah						
John	Baker						
Anthony	Byrne						

Supplemental Online Content: Nonauthor Collaborators

*Indicates required information. Only first name, last name, and suffix will appear in PubMed.

First Name, Middle Initial*	Last Name*	Suffix*	Academic Degrees	Institution	Location (city, state/province, country)	Role or Contribution, eg, chair, principal investigator	Subgroup, eg, Steering Committee
Caroline	Mcghee						
Amanda	Smart						
Claire	Copeland						
Michael	Carpenter						
Marion	Walker						
Richard	Davey						
Ann	Needle						
Razik	Fathima						
Gavin	Bateman						
Andrew	Stanners						
Prabal	Datta						
Linda	Jackson						
Julie	Ball						
Michelle	Davis						
Natalie	Atkinson						
Michelle	Fawcett						
Teresa	Thompson						
Helen	Guy						
Valerie	Hogg						
Carole	Hays						
Stephen	Woodward						
Mohammad	Haque						
Stuart	Symonds						
Mehran	Maanoosi						
Jane	Herman						
Toby	Black						
Caroline	Clarke						
Skelton	Miriam						
Alpha	Anthony						

*Indicates required information. Only first name, last name, and suffix will appear in PubMed.

First Name, Middle Initial*	Last Name*	Suffix*	Academic Degrees	Institution	Location (city, state/province, country)	Role or Contribution, eg, chair, principal investigator	Subgroup, eg, Steering Committee
Michele	Tribbeck						
Julie	Cronin						
Denise	Mead						
Ruth	Fennelly						
James	McIlmoyle						
Christina	Dickinson						
Carol	Jeffs						
Joanne	Howard						
Sajjad	Anwar						
Kirsty	Jones						
Saikat	Dhar						
Muhammad	Siddiq						
Caroline	Clay						
Simone	Ivatts						
Yolanda	Baird						
Moore	Sally						
Lisa	Clayton- Evans						
Sophie	Newton						
Isobel	Amey						
Indra	Chadbourn						
Rayessa	Rayessa						
Lisa	Wilson						
Alicia	Rodgers						
Charde	Naylor						
Sarah	Wilson						
Emma	Clarkson						
Ruth	Davies						
Paula	Owings						
Graeme	Sangster						

*Indicates required information. Only first name, last name, and suffix will appear in PubMed.

First Name, Middle Initial*	Last Name*	Suffix*	Academic Degrees	Institution	Location (city, state/province, country)	Role or Contribution, eg, chair, principal investigator	Subgroup, eg, Steering Committee
Valerie	Gott						
Victoria	Little						
Pauline	Weir						
Suja	Cherian						
Deepa	Jose						
Helen	Moroney						
Susan	Downham						
Angela	Dodd						
Venetia	Vettimootal Johnson						
Laura	Codd						
Naomi	Robinson						
Ashraf	Ahmed						
Sharon	Johnson						
Carol	Denniss						
Mo	Albazzaz						
Mishell	Cunningham						
Tajammal	Zahoor						
Timothy	Webster						
Sandra	Leason						
Syed	Haider						
Kausic	Chatterjee						
Arumugam	Nallasivan						
Charlotte	Perkins						
Samantha	Seagrave						
Colin	Jenkins						
Fiona	Price						
Claire	Hughes						
Lily	Mercer						
Malik	Hussain						

Supplemental Online Content: Nonauthor Collaborators

*Indicates required information. Only first name, last name, and suffix will appear in PubMed.

First Name, Middle Initial*	Last Name*	Suffix*	Academic Degrees	Institution	Location (city, state/province, country)	Role or Contribution, eg, chair, principal investigator	Subgroup, eg, Steering Committee
Sarah	Brown						
Miriam	Harvey						
Robert	Whiting						
Mohammad	Khan						
Jane	Homan						
Leanne	Foote						
Nicholas	Hunt						
Helen	Durman						
Lucy	Brotherton						
Jayne	Foot						
Corinne	Pawley						
Eliza	Foster						
Alison	Whitcher						
Kneale	Metcalf						
Jenny	Jagger						
Susan	McDonald						
Kelly	Waterfield						
Patrick	Sutton						
Naval	Shinh						
Ajmal	Anversha						
Garth	Ravenhill						
Richard	Greenwood						
Janak	Saada						
Alison	Wiltshire						
Rebekah	Perfitt						
Magdalini	Krommyda						
Evelyne	Burssens						
Naveen	Gadapa						
Karen	Dunne						

Supplemental Online Content: Nonauthor Collaborators

*Indicates required information. Only first name, last name, and suffix will appear in PubMed.

First Name, Middle Initial*	Last Name*	Suffix*	Academic Degrees	Institution	Location (city, state/province, country)	Role or Contribution, eg, chair, principal investigator	Subgroup, eg, Steering Committee
Sreeman	Andole						
Sam	King						
Catherine	Plewa						
Nigel	Smyth						
Jenny	Wilson						
Elio	Giallombardo						
Charlotte	Eglinton						
Lucy	Sykes						
Pradeep	Kumar						
James	Barker						
Linda	Dunn						
Isabel	Huggett						
Charlotte	Culmsee						
Philip	Thomas						
Min	Myint						
Helen	Brew						
Nikhil	Majmudar						
Janice	Oconnell						
George	Bunea						
Charlotte	Fox						
Diane	Gulliver						
Andrew	Smith						
Betty	Mokoena						
Naweed	Sattar						
Ramesh	Krishnamurthy						
Emily	Osborne						
David	Wilson						
Belinda	Wroath						
Kevin	Dynan						

Supplemental Online Content: Nonauthor Collaborators

*Indicates required information. Only first name, last name, and suffix will appear in PubMed.

First Name, Middle Initial*	Last Name*	Suffix*	Academic Degrees	Institution	Location (city, state/province, country)	Role or Contribution, eg, chair, principal investigator	Subgroup, eg, Steering Committee
Michael	Power						
Susan	Thompson						
Victoria	Adell						
Enoch	Orugun						
Una	Poultney						
Rachel	Glover						
Hannah	Crowther						
Sarah	Thornthwaite						
Ivan	Wiggam						
Aine	Wallace						
Enda	Kerr						
Ailsa	Fulton						
Annemarie	Hunter						
Suzanne	Tauro						
Sarah	Cuddy						
David	Mangion						
Skarlet	Markova						
Anne	Hardwick						
Tara	Lawrence						
Carmen	Constantin						
Jo	Fletecher						
Isobel	Thomas						
Kerry	Pettitt						
Lakshmanan	Sekaran						
Frances	Justin						
Margaret	Tate						
Kiranjit	Bharaj						
Rohan	Simon						
Niaz	Mohammed						

Supplemental Online Content: Nonauthor Collaborators

*Indicates required information. Only first name, last name, and suffix will appear in PubMed.

First Name, Middle Initial*	Last Name*	Suffix*	Academic Degrees	Institution	Location (city, state/province, country)	Role or Contribution, eg, chair, principal investigator	Subgroup, eg, Steering Committee
Sakthivel	Sethuraman						
Duke	Phiri						
Meena	Chauhan						
Khaled	Elfandi						
Samantha	Stafford						
Uzma	Khan						
Julie	Reddan						
David	Eveson						
Amit	Mistri						
Lisa	Manning						
Shagufta	Khan						
Champa	Patel						
Mohammed	Moqsith						
Saira	Sattar						
Man Yee	Lam						
Kashif	Musarrat						
Claire	Stephens						
Latheef	Kalathil						
Richard	Miller						
Maqsud	Salehin						
Nikki	Gautam						
Duncan	Bailey						
Amor	Kelly						
Julie	Meir						
Javed	Imam						
Lisa	Wood						
Anne	Nicolson						
Julie	White						
Mahmud	Sajid						

Supplemental Online Content: Nonauthor Collaborators

*Indicates required information. Only first name, last name, and suffix will appear in PubMed.

First Name, Middle Initial*	Last Name*	Suffix*	Academic Degrees	Institution	Location (city, state/province, country)	Role or Contribution, eg, chair, principal investigator	Subgroup, eg, Steering Committee
George	Ghaly						
Margaret	Ball						
Rachel	Gascoyne						
Harald	Proeschel						
Simon	Sharpe						
Sarah	Horton						
Emily	Beaves						
Stephanie	Jones						
Brigitte	Yip						
Murdina	Bell						
Linda	MacLiver						
Brian	MacInnes						
Derek	Esson						
Don	Sims						
Jennifer	Hurley						
Mark	Willmot						
Claire	Sutton						
Edward	Littleton						
Susan	Maiden						
Rachael	Jones						
James	Cunningham						
Carole	Green						
Michelle	Bates						
Raj	Shekhar						
Kelly	Waterfield						
Ellie	Gilham						
Iman	Ahmed						
Rachel	Crown						
Tracy	Fuller						

Supplemental Online Content: Nonauthor Collaborators

*Indicates required information. Only first name, last name, and suffix will appear in PubMed.

First Name, Middle Initial*	Last Name*	Suffix*	Academic Degrees	Institution	Location (city, state/province, country)	Role or Contribution, eg, chair, principal investigator	Subgroup, eg, Steering Committee
Neetish	Goorah						
Angela	Bell						
Christine	Kelly						
Arun	Singh						
Jamie	Walford						
Benjamin	Tomlinson						
Farzana	Patel						
Stephen	Duberley						
Ingrid	Kane						
Nicola	Gainsborough						
Chakravarthi	Rajkumar						
Jane	Gaylard						
Joanna	Breeds						
Alexandra	Pitt-Ford						
Emma	Barbon						
Laura	Latter						
Philip	Thompson						
Simon	Hervey						
Joseph	Vassallo						
Shrivakumar	Krishnamoorthy						
Deborah	Walter						
Helen	Cochrane						
Meena	Srinivasan						
Robert	Campbell						
Denise	Donaldson						
Nichola	Motherwell						
Frances	Hurford						
Indranil	Mukherjee						
Antony	Kenton						

Supplemental Online Content: Nonauthor Collaborators

*Indicates required information. Only first name, last name, and suffix will appear in PubMed.

First Name, Middle Initial*	Last Name*	Suffix*	Academic Degrees	Institution	Location (city, state/province, country)	Role or Contribution, eg, chair, principal investigator	Subgroup, eg, Steering Committee
Sheila	Nyabadza						
Irene	Martin						
Benjamin	Hunt						
Hardi	Hassan						
Bander	Dallol						
Sarah	O'toole						
Janet	Putterill						
Ratneshwari	Jha						
Rachel	Gallifent						
Puneet	Kakar						
Aparna	Pusalkar						
Kelly	Chan						
Puneet	Dangri						
Hannah	Beadle						
Angela	Cook						
Karen	Crabtree						
Santhosh	Subramonian						
Natalie	Temple						
Peter	Owusu-Agyei						
Nicola	Butterworth-Cowin						
Suzanne	Ragab						
Kerstin	Knops						
Emma	Jinks						
Judith	Dube						
Christine	Dickson						
Laura	Gleave						
Jacqui	Leggett						
Tatiana	Garcia						
Sissy	Ispoglou						

Supplemental Online Content: Nonauthor Collaborators

*Indicates required information. Only first name, last name, and suffix will appear in PubMed.

First Name, Middle Initial*	Last Name*	Suffix*	Academic Degrees	Institution	Location (city, state/province, country)	Role or Contribution, eg, chair, principal investigator	Subgroup, eg, Steering Committee
Rachel	Evans						
Sandeep	Ankolekar						
Anne	Hayes						
Hlaing	Ni						
Josette	Milligan						
Bithi	Rahman						
Carol	Graham						
Josin	Jose						
Breffni	Keegan						
Mandy	Doherty						
Jim	Kelly						
Caroline	Blair						
Donal	Concannon						
James	White						
Kelly	Thomas						
Lisa	Mellish						
Richard	Dewar						
