# Supplementary material for: Effects of Antiplatelet Therapy After Stroke Caused by Intracerebral Hemorrhage: Extended Follow-up of the RESTART Randomized Clinical Trial
Source: JAMA Neurol. 2021 Sep 3;78(10):1–8. doi: 10.1001/jamaneurol.2021.2956 (PMC8417806; doi:10.1001/jamaneurol.2021.2956)
Supplement: Supplement 4. — Data Sharing Statement [file jamaneurol-e212956-s004.pdf]

Data Sharing Statement

Al-Shahi Salman. Effects of Antiplatelet Therapy After Stroke Caused by Intracerebral Hemorrhage. *JAMA Neurol*. Published September 03, 2021. doi:10.1001/jamaneurol.2021.2956

Data
Data available: Yes
Data types: Deidentified participant data, Data dictionary
How to access data: University of Edinburgh Datashare
When available: beginning date: September 3, 2022

Supporting Documents
Document types: None

Additional Information
Who can access the data: Researchers whose proposed use of the data has been approved
Types of analyses: Specified purposes
Mechanisms of data availability: After approval of a proposal
